# AMPK is necessary for Treg functional adaptation to microenvironmental stress during malignancy and viral pneumonia

**DOI:** 10.1172/JCI179572

**Published:** 2025-03-18

**Authors:** Manuel A. Torres Acosta, Jonathan K. Gurkan, Qianli Liu, Nurbek Mambetsariev, Carla Reyes Flores, Kathryn A. Helmin, Anthony M. Joudi, Luisa Morales-Nebreda, Kathleen Cheng, Hiam Abdala-Valencia, Samuel E. Weinberg, Benjamin D. Singer

**Affiliations:** 1 Division of Pulmonary and Critical Care Medicine,; 2 Medical Scientist Training Program,; 3 Driskill Graduate Program,; 4 Division of Allergy and Immunology,; 5 Department of Dermatology,; 6 Department of Pathology,; 7 Department of Biochemistry and Molecular Genetics,; 8 Simpson Querrey Institute for Epigenetics, and; 9 Simpson Querrey Lung Institute for Translational Science (SQ LIFTS), Northwestern University Feinberg School of Medicine, Chicago, Illinois, USA.

**Keywords:** Immunology, Oncology, Pulmonology, Intermediary metabolism, Mitochondria, T cells

## Abstract

CD4^+^FOXP3^+^ Treg cells maintain self tolerance, suppress the immune response to cancer, and protect against tissue injury during acute inflammation. Treg cells require mitochondrial metabolism to function, but how Treg cells adapt their metabolic programs to optimize their function during an immune response occurring in a metabolically stressed microenvironment remains unclear. Here, we tested whether Treg cells require the energy homeostasis–maintaining enzyme AMPK to adapt to metabolically aberrant microenvironments caused by malignancy or lung injury, finding that AMPK is dispensable for Treg cell immune-homeostatic function but is necessary for full Treg cell function in B16 melanoma tumors and during influenza virus pneumonia. AMPK-deficient Treg cells had lower mitochondrial mass and exhibited an impaired ability to maximize aerobic respiration. Mechanistically, we found that AMPK regulates DNA methyltransferase 1 to promote transcriptional programs associated with mitochondrial function in the tumor microenvironment. During viral pneumonia, we found that AMPK sustains metabolic homeostasis and mitochondrial activity. Induction of DNA hypomethylation was sufficient to rescue mitochondrial mass in AMPK-deficient Treg cells, linking AMPK function to mitochondrial metabolism via DNA methylation. These results define AMPK as a determinant of Treg cell adaptation to metabolic stress and offer potential therapeutic targets in cancer and tissue injury.

## Abstract 2

Regulatory T cells require AMPK-mediated regulation of DNMT1 to promote mitochondrial capacity and exert their function in metabolically aberrant microenvironments caused by malignancy and pneumonia.

## Graphical abstract

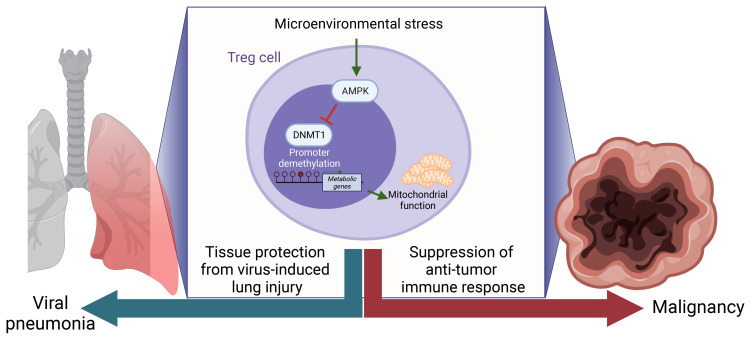

## Introduction

Regulatory T (Treg) cells are a subset of CD4^+^ T cells defined by the expression of the Forkhead box P3 (FOXP3) transcription factor that maintain self tolerance via the suppression of self-reactive effector immune cells (1, 2). Treg cells also regulate immune responses to cancer and acute inflammatory processes such as infections and tissue injury (3). In the tumor microenvironment (TME), Treg cell–mediated immune suppression becomes maladaptive and dampens the antitumor immune response to promote tumor progression (4, 5). In contrast, during acute inflammation such as in viral pneumonia, Treg cells promote tissue protection and recovery by restraining inflammation and coordinating the repair of the injured lung parenchyma (6, 7). Treg cell suppressive function is regulated by cellular metabolism, and, while Treg cells upregulate glucose consumption when proliferating, their suppressive function requires oxidative phosphorylation and is dependent on mitochondrial metabolism (8–11). The central role of cellular metabolism in determining Treg cell function has been well described in the TME, where Treg cells rewire their nutrient uptake to adapt to the metabolic aberrations of the microenvironment and thereby sustain their suppressive function (12, 13). Despite the known causal association between cellular metabolism and Treg cell function, how Treg cells sense microenvironmental changes and undergo metabolic adaptation during microenvironmental stress to optimize their suppressive function is unclear.

Adenosine monophosphate–activated (AMP-activated) protein kinase (AMPK) is a heterotrimeric protein complex that serves as a master regulator of cellular metabolism (14). In settings of energetic stress, AMP binds to AMPK and promotes its activation, priming the complex to phosphorylate downstream targets that mediate the restoration of energy homeostasis via one of two α-catalytic subunits (AMPKα1, encoded by the *Prkaa1* gene or AMPKα2, encoded by the *Prkaa2* gene) (15, 16). Canonically, AMPK effects its energy-replenishing function through the phosphorylation of cytoplasmic factors; however, in vitro studies support an emerging role for AMPK as a regulator of epigenetic modifiers, including DNA methyltransferase 1 (DNMT1) (17, 18). Whether AMPK activates metabolic transcriptional programs via epigenetic mechanisms in immune cells in vivo remains unknown.

The 2 isoforms of the catalytic subunit of AMPK (AMPKα1/α2) are dispensable for in vivo Treg cell–mediated immune self tolerance, but it is unclear whether Treg cells require AMPKα1/α2 to regulate acute immune responses in metabolically stressed microenvironments (19–22). Considering AMPK’s role in sustaining energy homeostasis via the potentiation of mitochondrial metabolism and the necessity of oxidative phosphorylation (OXPHOS) for Treg cell suppressive function, we hypothesized that Treg cells require AMPK during states of metabolic stress to potentiate mitochondrial metabolism and thereby optimize Treg cell suppressive function. To test our hypothesis, we generated Treg cell–specific AMPKα1- and AMPKα2-deficient mice (*Prkaa1^fl/fl^Prkaa2^fl/fl^Foxp3^YFP–Cre^*, referred to here as *Prkaa1/2^fl/fl^Foxp3^YFP–Cre^* mice) and challenged them with either subcutaneous B16 melanoma tumors or intratracheal inoculations of influenza A/WSN/33 H1N1 virus, disease models whose outcomes are dependent on Treg cell function and whose microenvironments are burdened with metabolic derangements that challenge cellular metabolism (4, 23). We confirmed that AMPKα1/α2 are dispensable for Treg cell–mediated immune self tolerance but found that *Prkaa1/2^fl/fl^Foxp3^YFP–Cre^* mice grew smaller tumors and experienced greater mortality and hypoxemia during influenza, with evidence of greater intratumoral and lung immune activation. Mechanistically, loss of AMPKα1/α2 in Treg cells resulted in promoter DNA hypermethylation at specific loci encoding metabolic genes, which were transcriptionally repressed. Consistent with this downregulation of metabolic gene expression, AMPKα1/α2–deficient Treg cells displayed impaired mitochondrial metabolism at homeostasis, in the TME, and in influenza virus–infected lungs. Pharmacological induction of DNA hypomethylation rescued mitochondrial mass in AMPKα1/α2–deficient Treg cells, demonstrating that DNA methylation regulates Treg cell mitochondrial mass in an AMPK-dependent manner. In summary, our data indicate that AMPK is necessary to maintain epigenetic and metabolic programs that support optimal Treg cell suppressive function in metabolically stressed microenvironments, such as the TME and the lung during viral pneumonia.

## Results

### AMPKα is dispensable for Treg cell suppressive function under homeostatic conditions.

**Figure 1:**
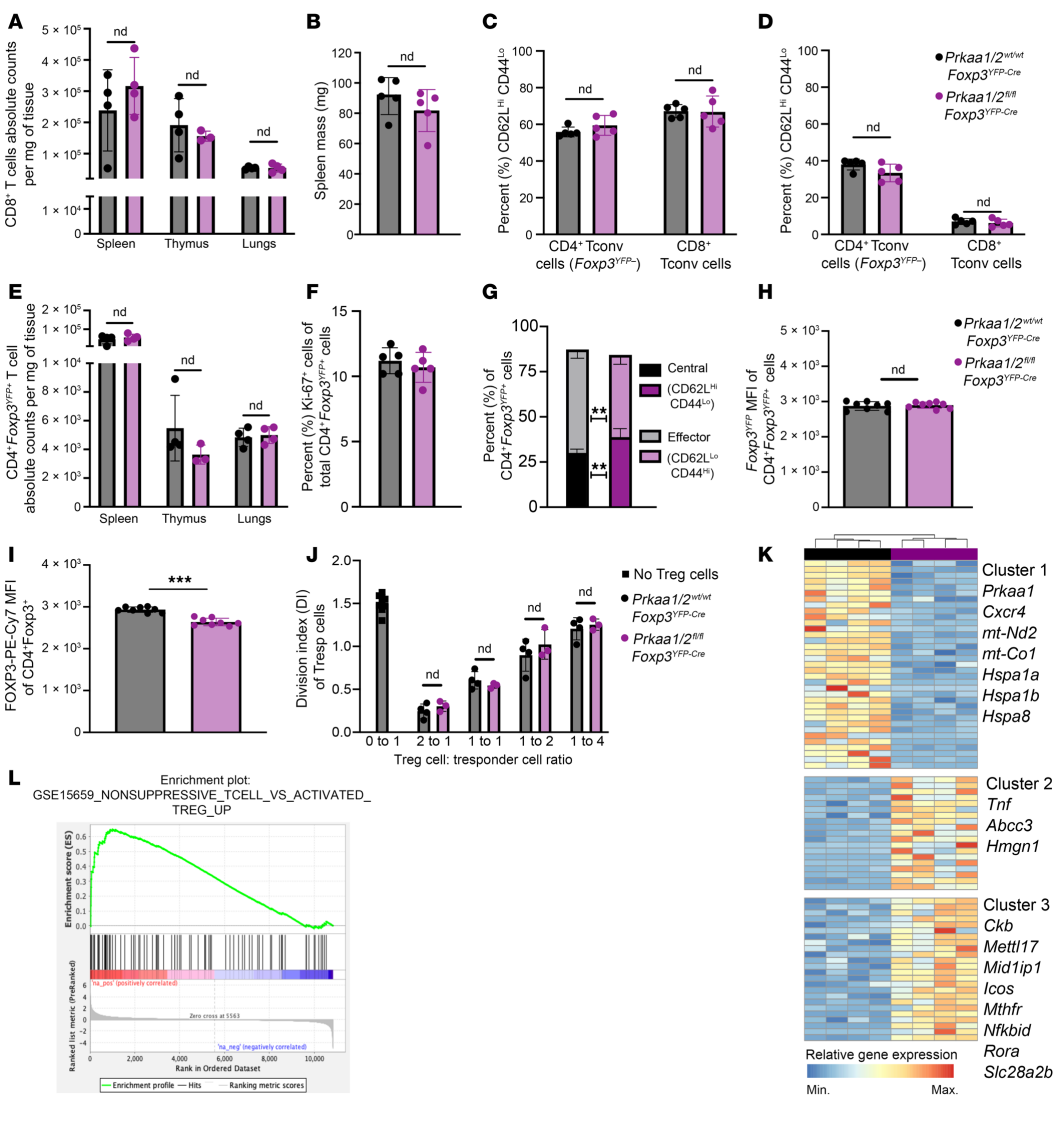
AMPKα1/α2 are dispensable for Treg cell–mediated immune self-tolerance and Treg cell suppressive function at homeostasis. (**A**) CD8^+^ conventional T (Tconv) cell absolute counts per milligram (mg) of *Prkaa1/2^wt/wt^Foxp3^YFP–Cre^* (control) and *Prkaa1/2^fl/fl^Foxp3^YFP–Cre^* mouse spleen (*n* = 4 control, *n* = 4 *Prkaa1/2^fl/fl^Foxp3^YFP–Cre^*), thymus (*n* = 4 control, *n* = 3 *Prkaa1/2^fl/fl^Foxp3^YFP–Cre^*), and lung (*n* = 4 control, *n* = 4 *Prkaa1/2^fl/fl^Foxp3^YFP–Cre^*). (**B**) Spleen mass of 8–12 week-old control (*n* = 5) and *Prkaa1/2^fl/fl^Foxp3^YFP–Cre^* (*n* = 5) mice. (**C** and **D**) Frequency of naive (CD62L^Hi^CD44^Lo^; **C**) and effector (CD62^Lo^CD44^Hi^; **D**) splenic CD8^+^ and CD4^+^ Tconv cells out of total CD8^+^ and CD4^+^ cells, respectively (*n* = 5 control, *n* = 5 *Prkaa1/2^fl/fl^Foxp3^YFP–Cre^*). (**E**) CD4^+^*Foxp3*^YFP+^ cell absolute counts per mg of control and *Prkaa1/2^fl/fl^Foxp3^YFP–Cre^* mouse spleen (*n* = 4 control, *n* = 4 *Prkaa1/2^fl/fl^Foxp3^YFP–Cre^*), thymus (*n* = 4 control, *n* = 3 *Prkaa1/2^fl/fl^Foxp3^YFP–Cre^*), and lung (*n* = 4 control, *n* = 4 *Prkaa1/2^fl/fl^Foxp3^YFP–Cre^*). (**F**) Frequency of Ki-67^+^CD4^+^*Foxp3*^YFP+^ cells out of total CD4^+^*Foxp3*^YFP+^ splenocytes (*n* = 5 control, *n* = 5 *Prkaa1/2^fl/fl^Foxp3^YFP–Cre^*). (**G**) Frequency of central (CD62L^Hi^CD44^Lo^) and effector (CD62^Lo^CD44^Hi^) CD4^+^*Foxp3*^YFP+^ cells of total CD4^+^*Foxp3*^YFP+^ splenocytes (*n* = 5 control, *n* = 5 *Prkaa1/2^fl/fl^Foxp3^YFP–Cre^*). (**H** and **I**) *Foxp3*^YFP^ (**H**) and FOXP3-PE-Cy7 (**I**) mean fluorescence intensity (MFI) of CD4^+^*Foxp3*^YFP+^ splenocytes (*n* = 8 control, *n* = 8 *Prkaa1/2^fl/fl^Foxp3^YFP–Cre^*). (**J**) Division index of CD4^+^*Foxp3*^YFP–^ splenic responder T (Tresp) cells cocultured with CD4^+^*Foxp3*^YFP+^ splenocytes (*n* = 4 control, *n*=3 *Prkaa1/2^fl/fl^Foxp3^YFP–Cre^*) for 72 hours. (**K**) *K*-means clustering of 78 significant differentially expressed genes (FDR *q* < 0.05) identified between splenic CD4^+^*Foxp3*^YFP+^ cells sorted from control (*n* = 4) and *Prkaa1/2^fl/fl^Foxp3^YFP–Cre^* (*n* = 4) mice with *k* = 3 and scaled as *Z*-scores across rows. (**L**) Enrichment plot of the GSE15659_NONSUPPRESSIVE_TCELL_Versus_ACTIVATED_TREG_UP gene set generated through gene set enrichment analysis (GSEA) preranked testing of the expressed genes of *Prkaa1/2^fl/fl^Foxp3^YFP–Cre^* and control splenic Treg cells identified by RNA-seq. **P or *q* < 0.01; ***P or *q* < 0.001; nd, no discovery, NS, not significant according to Mann-Whitney *U* test (**B**, **F**, **H**, and **I**) with 2-stage linear step-up procedure of Benjamini, Krieger, and Yekutieli with Q = 5% (**A**, **C**–**E**, **G**, and **J**).

We confirmed loss of AMPKα1/α2 in CD4^+^*Foxp3*^YFP+^ Treg cells (see Supplemental Figure 1A for gating strategy; supplemental material available online with this article; https://doi.org/10.1172/JCI179572DS1) isolated from *Prkaa1/2^fl/fl^Foxp3^YFP–Cre^* mice, which bred in approximately mendelian sex ratios (Supplemental Figure 1, B–E). Consistent with previous reports (19–22), a tissue survey of spleen, thymus, and lungs did not reveal significant differences in CD8^+^ T cell infiltration between *Prkaa1/2^fl/fl^Foxp3^YFP–Cre^* and *Prkaa1/2^wt/wt^Foxp3^YFP–Cre^* (control) mice (Figure 1A). There were no significant differences between *Prkaa1/2^fl/fl^Foxp3^YFP–Cre^* and control mice in their spleen mass or the relative proportion of naive (CD62L^Hi^CD44^Lo^) and effector (CD62L^Lo^CD44^Hi^) splenic conventional T (Tconv) cells (Figure 1, B–D), supporting a lack of spontaneous inflammation resulting from Treg cell–specific loss of AMPKα1/α2. Although the total number and proliferation rate of Treg cells was not significantly different between groups (Figure 1, E–F), the splenic Treg cell compartment in *Prkaa1/2^fl/fl^Foxp3^YFP–Cre^* mice displayed a nominal yet statistically significant shift toward a central (CD62L^Hi^CD44^Lo^) Treg cell phenotype relative to control mice (Figure 1G). The *Foxp3^YFP–Cre^* allele used to drive *Foxp3*-dependent expression of Cre recombinase also drives expression of yellow fluorescent protein (YFP), which serves as a transcriptional reporter for the *Foxp3* locus. Treg cells from *Prkaa1/2^fl/fl^Foxp3^YFP–Cre^* mice showed similar expression of *Foxp3*-YFP to control mice, although we detected slightly lower FOXP3 protein in AMPKα1/α2–deficient splenic Treg cells measured by direct conjugated antibody staining (Figure 1, H and I). AMPKα1/α2–deficient Treg cells displayed no significant differences in their ability to suppress responder CD4^+^ Tconv cell proliferation in vitro relative to controls (Figure 1J) and showed no significant differences in their surface membrane levels of markers traditionally correlated with Treg cell suppressive function (CD25, CTLA-4, PD-1, TIGIT, and ICOS, Supplemental Figure 1, F–J) or their proliferation rate in vitro (Supplemental Figure 1K). Pharmacologic activation of AMPK promotes *Foxp3* expression in vitro (24, 25). To test whether AMPKα1/α2 are necessary for the stability of induced (i)Treg cells in vitro, we subjected CD4^+^*Foxp3*^YFP–^ Tconv cells sorted from *Prkaa1/2^fl/fl^Foxp3^YFP–Cre^* and control mice to Treg cell–polarizing conditions for 5 days. We detected no significant difference in *Foxp3* expression between groups after 5 days in culture (Supplemental Figure 1L).

We also performed unsupervised assessment of the metabolome of AMPKα1/α2–deficient and control splenic Treg cells using liquid chromatography tandem mass spectrometry (LC-MS) but only found 15 differentially represented metabolites (log_2_ fold change > 0.5, –log_10_ *P* value > 1) between groups across the measured metabolites (258 annotated metabolites, Supplemental Figure 1, M and N, and Supplemental Data Set 1). Finally, we assessed the transcriptional state of AMPKα1/α2–deficient and control splenic Treg cells at homeostasis via RNA-seq and identified 78 differentially expressed genes (DEGs) (Figure 1K and Supplemental Data Set 2). Among the genes downregulated in AMPKα1/α2–deficient splenic Treg cells were components of the electron transport chain (*mt-Nd2* and *mt-Co1*) and heat shock proteins (*Hspa1a*, *Hspa1b*, and *Hspa8*), consistent with AMPK’s positive regulation of mitochondrial metabolism and the cellular stress response. Genes upregulated in AMPKα1/α2–deficient splenic Treg cells included cytokines and transcription factors associated with effector T cell function (*Tnf*, *Nfkbid*, and *Rora*) and regulators of 1-carbon metabolism (*Mthfr*). Gene set enrichment analysis (GSEA) demonstrated downregulation of genes associated with Treg cell identity and function (26) (Figure 1L), suggesting that, although AMPKα1/α2 are dispensable for Treg cell–mediated immune self tolerance during development and homeostasis, AMPKα1/α2–deficient Treg cells may suffer functional impairment in settings that drive enhanced suppressive function, such as the TME.

### AMPKα promotes Treg cell suppressive function in the TME.

**Figure 2:**
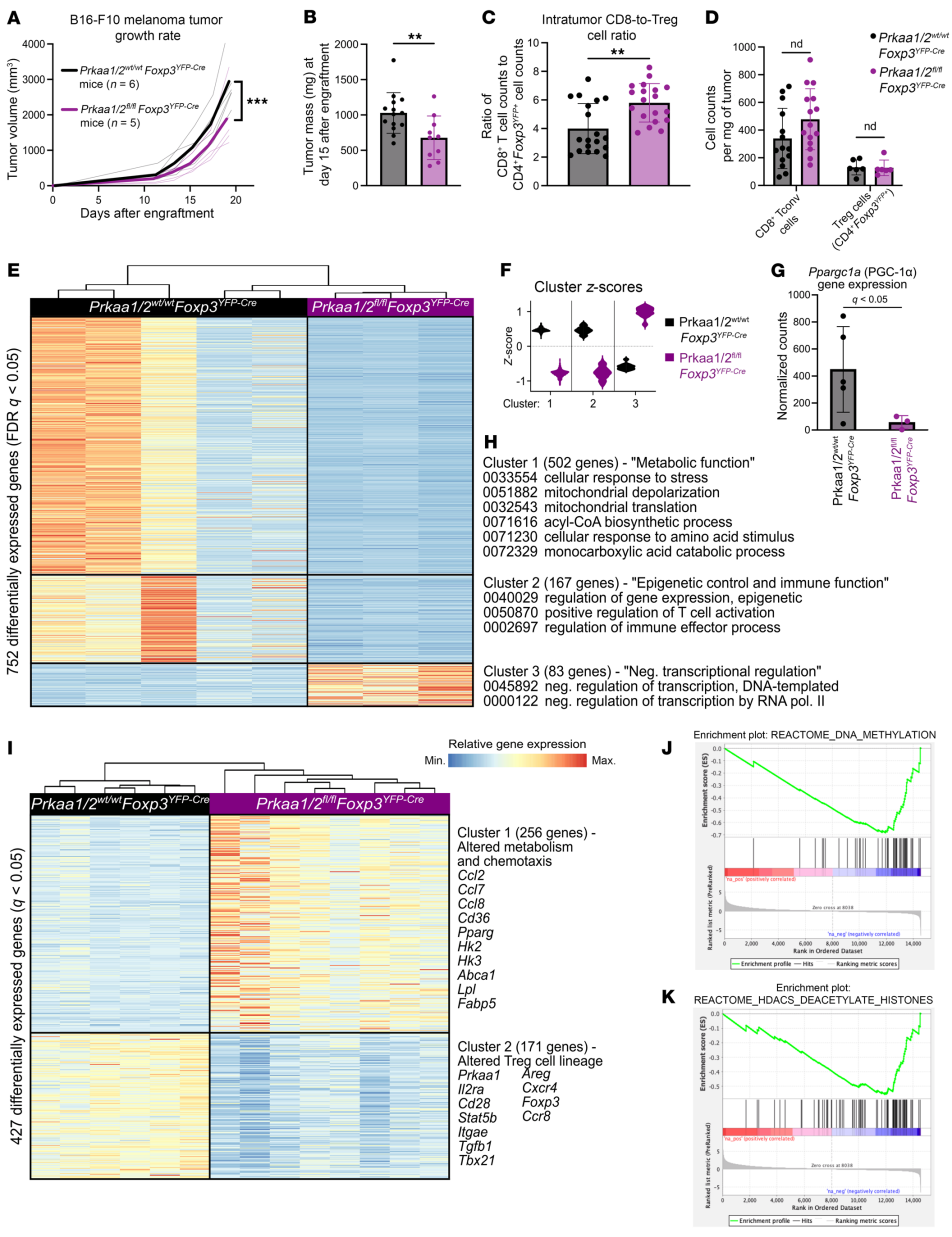
AMPKα1/α2 loss is sufficient to impair Treg cell suppressive function in the TME. (**A**) Growth of B16 melanoma tumors in *Prkaa1/2^wt/wt^Foxp3^YFP–Cre^* (control, *n* = 6) and *Prkaa1/2^fl/fl^Foxp3^YFP–Cre^* (*n* = 5) mice. (**B**) Tumor mass of control (*n* = 14) and *Prkaa1/2^fl/fl^Foxp3^YFP–Cre^* (*n* = 10) mice at day 15 after engraftment. (**C**) Ratio of live CD8^+^ cell counts to live CD4^+^*Foxp3*^YFP+^ (Treg) cell counts in B16 melanoma tumors of control (*n* = 19) and *Prkaa1/2^fl/fl^Foxp3^YFP–Cre^* (*n* = 19) mice at day 15 after engraftment. (**D**) Absolute counts of CD8^+^ Tconv cells and Treg cells per mg of tumor from control (*n* = 14, CD8^+^ Tconv cells; *n* = 6, Treg cells) and *Prkaa1/2^fl/fl^Foxp3^YFP–Cre^* mice (*n* = 15, CD8^+^ Tconv cells; *n* = 6, Treg cells). (**E**) *K*-means clustering of differentially expressed genes (FDR *q* < 0.05) identified between Treg cells sorted from B16 melanoma tumors of control (*n* = 5) and *Prkaa1/2^fl/fl^Foxp3^YFP–Cre^* (*n* = 3) mice at day 15 after engraftment with *k* = 3 and scaled as *z*-scores across rows. (**F**) Average *z*-scores for the 3 clusters shown in (**E**). (**G**) *Ppargc1a* expression (*n* = 5 control, *n* = 3 *Prkaa1/2^fl/fl^Foxp3^YFP–Cre^*). (**H**) Selection of top gene ontology (GO) processes (FDR *q* < 0.05). (**I**) *K*-means clustering of differentially expressed genes (FDR *q* < 0.05) identified between Treg cells sorted from B16 melanoma tumors of control (*n* = 6) and *Prkaa1/2^fl/fl^Foxp3^YFP–Cre^* (*n* = 6) mice at day 12 after engraftment with *k* = 2 and scaled as *z*-scores across rows. (**J** and **K**) GSEA preranked test enrichment plots (*P* < 0.05, FDR *q* < 0.25) of the REACTOME_DNA_METHYLATION (**J**) and REACTOME_HDACS_DEACETYLATE_HISTONES (**K**) from tumor-infiltrating *Prkaa1/2^fl/fl^Foxp3^YFP–Cre^* and control Treg cells on day 12 after engraftment. ***P < 0.001 according to 2-way ANOVA with 2-stage linear step-up procedure of Benjamini, Krieger, and Yekutieli with Q = 5% (**A**). **P < 0.01. according to Mann Whitney U test (**B** and **C**). 1 outlier was identified and excluded from (**B**) and 2 from (**C**) using the ROUT method (Q = 0.5%).

While we did not detect *Prkaa2* expression in splenic and lymph node Treg cells of mice bearing subcutaneous B16 melanoma tumor grafts, we found that control Treg cells upregulated the expression of *Prkaa1* and *Prkaa2* in the TME (Supplemental Figure 2, A and B). Hence, to determine whether AMPKα1/α2–deficient Treg cells are functionally impaired in the TME, we challenged *Prkaa1/2^fl/fl^Foxp3^YFP–Cre^* and control mice with B16 melanoma tumors, finding that *Prkaa1/2^fl/fl^Foxp3^YFP–Cre^* mice experienced lower tumor volume over time and lower tumor weights at day 15 after engraftment (Figure 2, A and B). Tumors of *Prkaa1/2^fl/fl^Foxp3^YFP–Cre^* mice had significantly higher CD8-to-Treg cell ratios relative to controls at day 15 after engraftment, with a trend toward higher absolute counts of CD8^+^ Tconv cells in the setting of comparable absolute Treg cell counts, consistent with a loss of Treg cell suppressive function in the TME (Figure 2, C and D). We did not find significant differences in the intratumor proportion of naive, central memory, or effector Tconv cell subsets between groups at day 15 after engraftment (Supplemental Figure 2, C–G). There were also no significant differences between the Treg cells of *Prkaa1/2^fl/fl^Foxp3^YFP–Cre^* and control mice in their abundance out of all CD4^+^ cells or their proportion of central versus effector subsets (Supplemental Figure 2, H–J). We assessed the production of IFN-γ and TNF-α by tumor-infiltrating CD8^+^ T cells and found a significantly greater proportion of IFN-γ^+^CD8^+^ T cells in tumors of *Prkaa1/2^fl/fl^Foxp3^YFP–Cre^* mice (Supplemental Figure 2, K and L). The proliferation, *Foxp3* gene expression, and FOXP3 protein expression of tumor-infiltrating Treg cells was not significantly different between groups, which was also true for most traditional surface markers of Treg cell suppressive function (Supplemental Figure 2, M–T). We leveraged RNA-seq to profile the transcriptional state of Treg cells sorted from the tumors of *Prkaa1/2^fl/fl^Foxp3^YFP–Cre^* and control mice at day 15 after engraftment and identified 752 DEGs (Figure 2E). Unsupervised clustering revealed that the 2 largest groups of DEGs (Clusters 1 and 2) were downregulated in AMPKα1/α2–deficient cells (Figure 2F and Supplemental Data Set 3). The *Ppargc1a* gene, encoding the master transcriptional regulator of mitochondrial biogenesis and function, PGC-1α, was significantly downregulated in Cluster 1 in *Prkaa1/2^fl/fl^Foxp3^YFP–Cre^* mice (Figure 2G). Accordingly, functional enrichment analysis demonstrated that Cluster 1 genes are involved in cellular metabolism and include Gene Ontology (GO) terms relating to cellular response to stress and mitochondrial metabolism; Cluster 2 genes are involved in immune effector cell programs and in epigenetic regulation of transcription (Figure 2H). Analysis of Cluster 3 genes, which were upregulated in AMPKα1/α2–deficient Treg cells, linked this cluster to a broad set of cellular functions including negative regulation of transcription. GSEA revealed a positive enrichment of genes associated with allograft rejection and IFN-γ signaling as well as a negative enrichment of genes associated with angiogenesis in tumor-infiltrating AMPKα1/α2–deficient Treg cells (Supplemental Figure 3, A–C), consistent with loss of Treg cell function in the TME. In addition, tumor-infiltrating AMPKα1/α2–deficient Treg cells also showed transcriptional signatures associated with downregulated response to hypoxia, glycolysis, and cholesterol homeostasis (Supplemental Figure 3, D–F), suggestive of failed metabolic adaptation in the TME. Because *Prkaa1/2^fl/fl^Foxp3^YFP–Cre^* mice had significantly lower tumor burden relative to control mice at day 15 after engraftment, we also sorted tumor-infiltrating Treg cells for RNA-seq profiling when tumor burden was comparable (day 12 after engraftment) and identified 427 DEGs (Figure 2I and Supplemental Data Set 4); *k*-means clustering yielded 2 clusters of genes. Cluster 1 contained genes significantly upregulated in AMPKα1/α2–deficient tumor-infiltrating Treg cells and included genes encoding chemokines (*Ccl2*, *Ccl7*, *Ccl8*), modulators of lipid metabolism (*Cd36*, *Pparg*, *Lpl*, *Abca1*), and glycolytic enzymes (*Hk2*, *Hk3*). Cluster 2 represented DEGs downregulated in AMPKα1/α2–deficient tumor-infiltrating Treg cells at day 12 after engraftment and included *Prkaa1*, along with a variety of Treg cell lineage markers (*Foxp3, Il2ra, Stat5b*) and mediators of Treg cell suppressive function (*Tgfb1*, *Cxcr4*, *Itgae*, *Ccr8*); notably, *Cxcr4* and *Ccr8* are known to be necessary for Treg cell suppressive function in the TME (27, 28). Moreover, GSEA revealed a negative enrichment of genes involved in DNA methylation and histone deacetylation in AMPKα1/α2–deficient tumor-infiltrating Treg cells at day 12 after engraftment (Figure 2, J and K). Collectively, these data indicate that tumor-infiltrating AMPKα1/α2–deficient Treg cells have impaired suppressive function, as evidenced by lower tumor burden over time, higher intratumoral CD8-to-Treg cell ratios, and higher frequency of IFN-γ^+^CD8^+^ cells in *Prkaa1/2^fl/fl^Foxp3^YFP–Cre^* mice relative to controls. Our data also suggest that the impaired suppressive function of AMPKα1/α2–deficient Treg cells in the TME is associated with a failure to upregulate metabolic and effector transcriptional programs and that this failed transcriptional adaptation is preceded by an attenuated Treg cell lineage transcriptional program as well as a differential enrichment of expressed genes that regulate epigenetic changes, including DNA methylation.

### AMPKα2 contributes to the regulation of Treg cell suppressive function in the TME.

Previous studies that evaluated the requirement of AMPK for Treg cell–mediated suppression of the antitumor immune response leveraged mouse models of Treg cell–specific AMPKα1-conditional knockout mice (*Prkaa1^fl/fl^Foxp3^YFP–Cre^*), and the results have conflicted on whether loss of AMPKα1 potentiates or compromises Treg cell function in the TME (29, 30). To test the relevance of our finding that Treg cells upregulate *Prkaa2* in the TME (see Supplemental Figure 2B), we bred Treg cell–specific AMPKα1-deficient (*Prkaa1^fl/fl^Foxp3^YFP–Cre^*) and AMPKα2-deficient (*Prkaa2^fl/fl^Foxp3^YFP–Cre^*) mice and evaluated their response to B16 melanoma tumor grafts. We observed significantly smaller tumors in Treg cell–specific AMPKα1-deficient mice relative to controls, while those with AMPKα2-deficient Treg cells exhibited significantly greater tumor volume over time through day 15 after tumor engraftment, relative to mice bearing control, AMPKα1-, and AMPKα1/α2–deficient Treg cells (Supplemental Figure 4, A and B). Consistent with this finding, *Prkaa1^fl/fl^Foxp3^YFP–Cre^* mice had higher CD8-to-Treg cell ratios compared with *Prkaa2^fl/fl^Foxp3^YFP–Cre^* and control mice (Supplemental Figure 4C). Tumors of *Prkaa2^fl/fl^Foxp3^YFP–Cre^* mice also exhibited a shift in their CD8^+^ Tconv cell compartment toward a central memory (CD62L^Hi^CD44^Lo^) phenotype (Supplemental Figure 4, D–F) and a nominally lower proportion of effector CD4^+^ Tconv cells relative to *Prkaa1^fl/fl^Foxp3^YFP–Cre^* and control mice (Supplemental Figure 4, G and H). *Prkaa1^fl/fl^Foxp3^YFP–Cre^* mice had a significantly higher proportion of effector CD8^+^ T cells (CD62L^Lo^CD44^Hi^) relative to *Prkaa2^fl/fl^Foxp3^YFP–Cre^* mice and had the lowest proportion of Treg cells out of the CD4^+^ T cell pool in their tumors (Supplemental Figure 4I). Nevertheless, we found no significant differences between groups in the proliferation rate of tumor-infiltrating Treg cells (Supplemental Figure 4J), their FOXP3 and CD25 protein levels measured by flow cytometry (Supplemental Figure 4, K and L), and the relative proportion of central versus effector Treg cell subsets (Supplemental Figure 4M). When assessing the status of markers of Treg cell suppressive function, we found that loss of AMPKα1 or AMPKα2 had opposing effects on tumor-infiltrating Treg cell PD-1 expression, with loss of AMPKα1 leading to lower levels and loss of AMPKα2 leading to higher levels (Supplemental Figure 4N). To determine the contribution of each AMPKα subunit to the transcriptional signature of AMPKα1/2–deficient Treg cells, we also sorted tumor-infiltrating Treg cells from *Prkaa1^fl/fl^Foxp3^YFP–Cre^* and *Prkaa2^fl/fl^Foxp3^YFP–Cre^* mice for RNA-seq at day 12 after engraftment. Treg cells sorted from tumors of *Prkaa1^fl/fl^Foxp3^YFP–Cre^* and *Prkaa2^fl/fl^Foxp3^YFP–Cre^* mice only had 29 and 35 DEGs relative to controls, respectively (Supplemental Figure 3, G and H, and Supplemental Data Sets 5 and 6). When compared with the 429 DEGs identified in Treg cells sorted from tumors of *Prkaa1/2^fl/fl^Foxp3^YFP–Cre^* mice, these data suggest that there is overlap but not complete redundancy in the downstream targets of AMPK complexes occupied by the different AMPKα subunits. GSEA once again revealed differential enrichment of genes associated with DNA methylation relative to controls in Treg cells sorted from tumors of *Prkaa1^fl/fl^Foxp3^YFP–Cre^* and *Prkaa2^fl/fl^Foxp3^YFP–Cre^* mice (Supplemental Figure 3, I and J), suggesting that both AMPKα subunits contribute to the epigenetic regulation of Treg cells mediated by AMPK complexes.

### The metabolic landscape of the virus-injured lung resembles the TME in its metabolite abundance; however, they differ in the abundance of key carbon sources.

**Figure 3:**
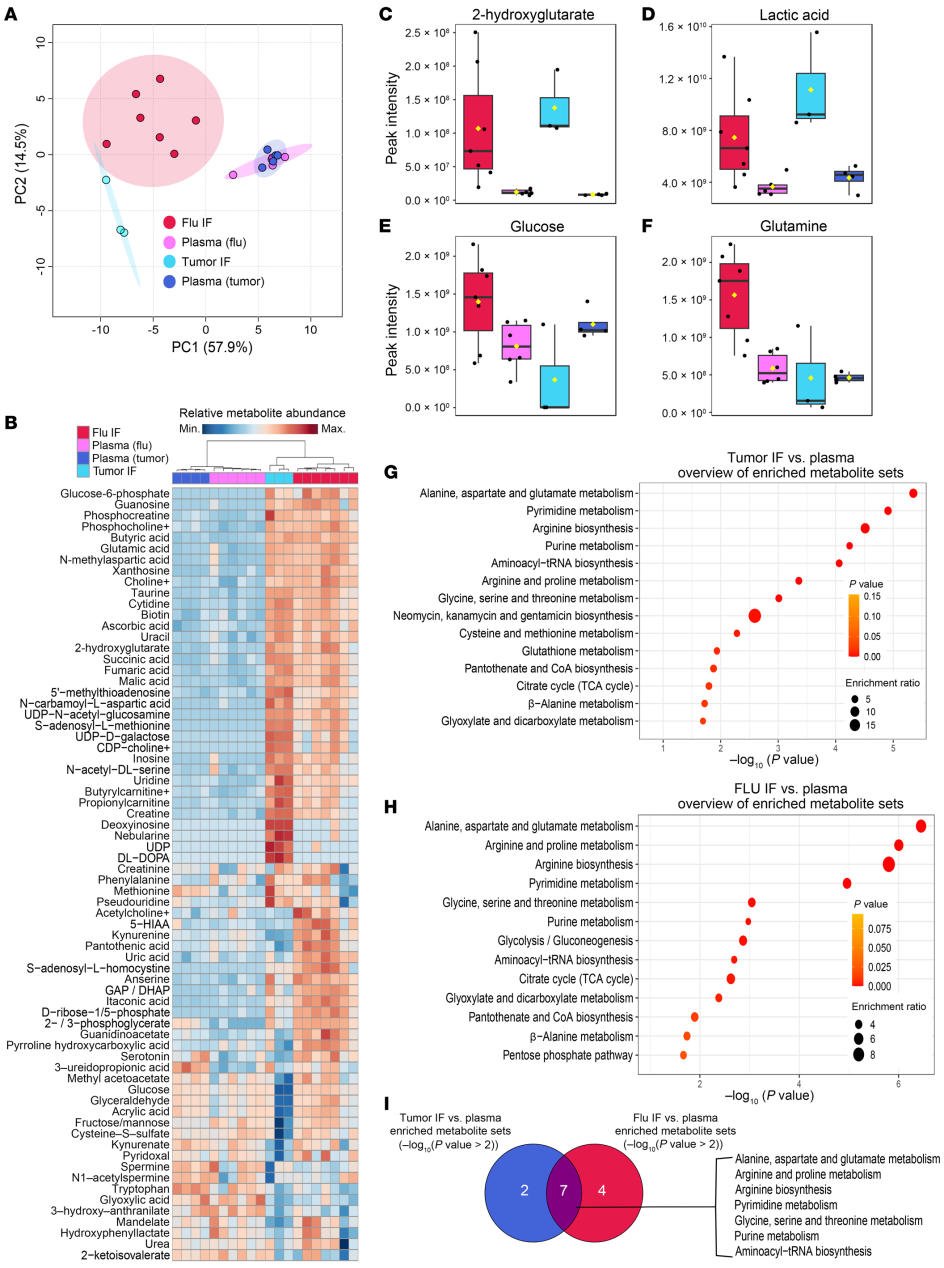
The metabolic landscape of the influenza virus–injured lung resembles the TME in its metabolite abundance; however, they differ in the abundance of key carbon sources. (**A**) Principal component (PC) analysis of the peak intensities of metabolites identified via liquid chromatography tandem mass spectrometry (LC-MS) from B16 melanoma tumor (*n* = 3) and influenza virus–infected lung (flu, *n* = 7) interstitial fluid (IF) and paired plasma (*n* = 4 tumor, *n* = 6 flu) from the same animals. (**B**) Heatmap of the 70 most differentially represented metabolites in plasma, tumor IF, and flu IF according to 1-way ANOVA (*P* < 0.1). (**C**–**F**) Abundance of key significant differentially represented metabolites: 2-hydroxyglutarate (**C**), lactic acid (**D**), glucose (**E**), and glutamine (**F**). (**G**) Results from overrepresentation analysis of the significant (*P* < 0.1) differentially represented metabolites between tumor IF and plasma. (**H**) Results from overrepresentation analysis of the significant (*P* < 0.1) differentially represented metabolites between flu IF and plasma. (**I**) Overlap in significantly (*P* < 0.1) enriched metabolite sets between tumor IF versus plasma comparison and flu IF versus plasma comparison according to overrepresentation analysis of flu IF versus plasma and tumor IF versus plasma.

Treg cells must adapt their metabolism to function in the metabolically deranged microenvironment of the TME (31), but it remains undetermined whether other inflammatory microenvironments where Treg cells have critical functions, such as the virally infected lung (6), exhibit similar metabolic aberrations, and thereby present similar environmental stress to Treg cells. To compare the metabolic changes that occur in the TME and the virus-injured lung, we collected interstitial fluid (IF) from mouse lungs 10 days after infection with influenza virus (flu), B16 melanoma tumors 15 days after engraftment, and paired plasma samples. We then measured hydrophilic metabolite abundance via LC-MS. Principal component (PC) analysis of the LC-MS data (303 annotated metabolites) revealed that the first principal component (PC1), which represents 57.9% of the variance in the dataset, captured the variance due to differences in metabolite abundance between flu and tumor IF relative to plasma (Figure 3, A and B, and Supplemental Data Set 7). Key metabolites that contributed to PC1 include 2-hydroxyglutarate and lactic acid, which are overrepresented in tumor and flu IF relative to plasma (Figure 3, C and D) and suggest a state of reduced mitochondrial electron transport chain activity in these disease microenvironments (31). The second principal component (PC2; 14.5% of the variance) was due to differences in metabolite abundance between flu and tumor IF. Interestingly, key carbon sources such as glucose and glutamine were more abundant in flu IF and less abundant in tumor IF relative to plasma (Figure 3, E and F). Overrepresentation analysis of the significant differentially represented metabolites in tumor IF relative to plasma (Figure 3G and Supplemental Figure 5, A and B) and flu IF relative to plasma (Figure 3H and Supplemental Figure 5, C and D) revealed that the TME and the lung during viral pneumonia undergo significant changes in similar metabolic pathways related to amino acid metabolism (Figure 3I). Nevertheless, direct comparison of tumor IF and flu IF metabolites revealed disease state–specific metabolite signatures, including an enrichment of metabolites related to tryptophan, cysteine, and methionine metabolism in flu IF (Supplemental Figure 6, A–D). These data suggest that Treg cells and other immune cells experience shared metabolic challenges in the TME and the injured lung during viral pneumonia but may use different carbon sources in these microenvironments.

### AMPKα promotes Treg cell tissue-protective function during lung injury from viral pneumonia.

**Figure 4:**
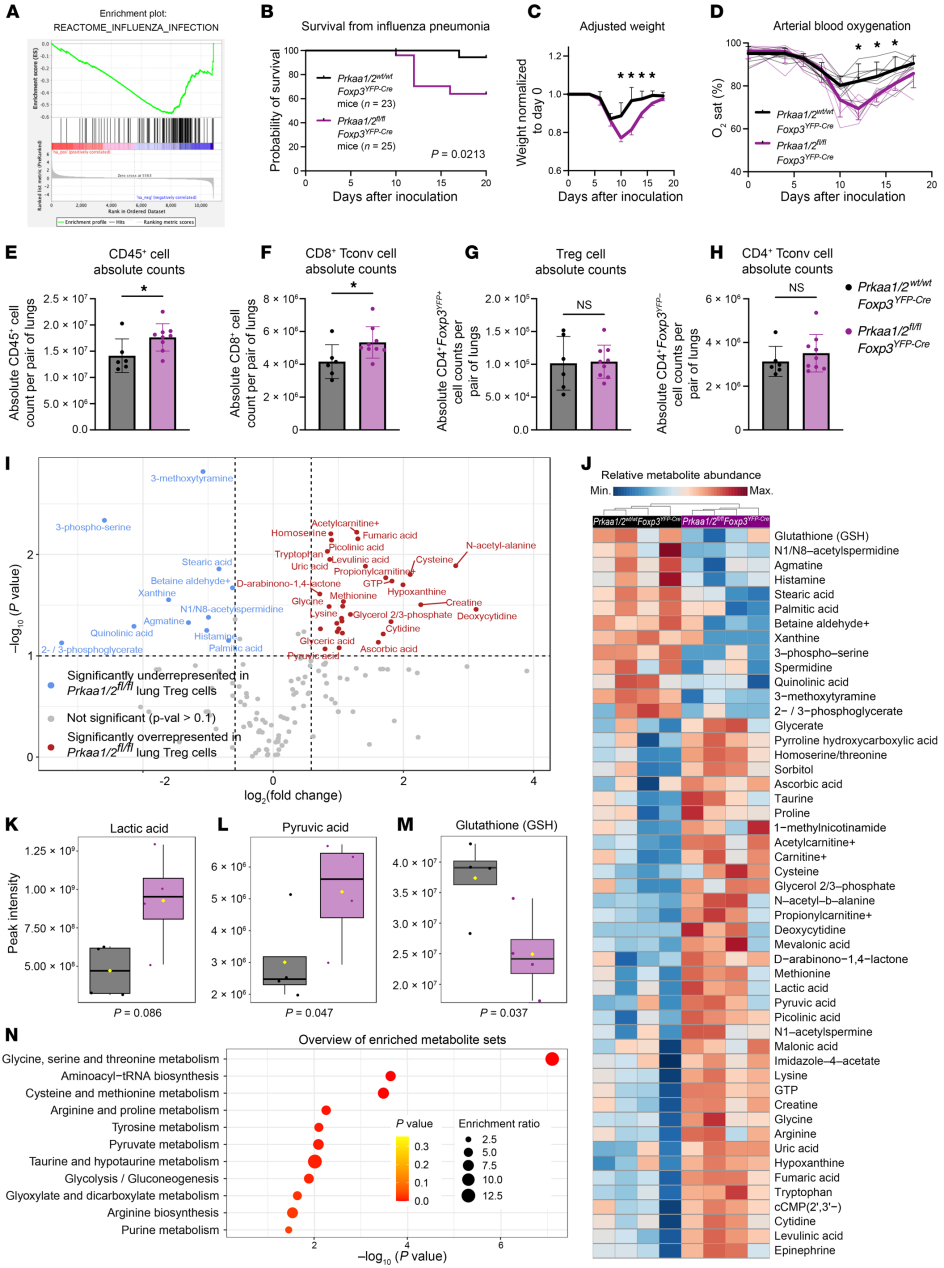
AMPKα1/α2 are necessary for optimal Treg cell function in the lung during influenza pneumonia. (**A**) Enrichment plot of the REACTOME_INFLUENZA_INFECTION gene set (*P* < 0.05, FDR *q* < 0.25) generated through GSEA preranked testing of the expressed genes of *Prkaa1/2^wt/wt^Foxp3^YFP–Cre^* (control) and *Prkaa1/2^fl/fl^Foxp3^YFP–Cre^* CD4^+^*Foxp3*^YFP+^ splenocytes identified by RNA-seq shown in Figure 1K. (**B**) Survival of control (*n* = 23) and *Prkaa1/2^fl/fl^Foxp3^YFP–Cre^* (*n* = 25) mice following intratracheal inoculation of 12.5 plaque forming units (PFUs) of influenza A/WSN/33 H1N1 (influenza) virus. (**C** and **D**) Weight (**C**), and arterial oxyhemoglobin saturation (**D**) over time of control (*n* = 6) and *Prkaa1/2^fl/fl^Foxp3^YFP–Cre^* (*n* = 8) mice following intratracheal inoculation of 12.5 PFUs of influenza virus. (**E**–**H**) Absolute counts of CD45^+^ cells (**E**), CD8^+^ cells (**F**), CD4^+^*Foxp3*^YFP+^ cells (**G**), and CD4^+^ cells (**H**) per pair of lungs in control (*n* = 6) and *Prkaa1/2^fl/fl^Foxp3^YFP–Cre^* (*n* = 9) mice at day 10 after influenza virus inoculation. (**I**) Volcano plot of abundance of metabolites detected in control (*n* = 4) and *Prkaa1/2^fl/fl^Foxp3^YFP–Cre^* (*n* = 4) Treg cells sorted from lungs at day 10 after influenza virus inoculation. (**J**) Heatmap of top 50 differentially represented metabolites between control (*n* = 4) and *Prkaa1/2^fl/fl^Foxp3^YFP–Cre^* (*n* = 4) Treg cells sorted from lungs at day 10 after influenza virus inoculation. (**K**–**M**) Peak intensities measured for lactic acid (**K**), pyruvic acid (**L**), and glutathione GSH (**M**) in Treg cells from the lungs of control (*n* = 4) and *Prkaa1/2^fl/fl^Foxp3^YFP–Cre^* (*n* = 4) mice at day 10 after influenza virus inoculation. (**N**) Results of overrepresentation analysis from the significant (*P* < 0.1, log_2_(FC) ≥ 1.5 or ≤ –1.5) differentially represented metabolites identified in **I**. Survival curve (**B**) *P* was determined using log-rank (Mantel-Cox) test. *q < 0.05 according to 2-way ANOVA with 2-stage linear step-up procedure of Benjamini, Krieger, and Yekutieli with Q = 5% (**C**–**D**). *P < 0.05, NS not significant according to Mann-Whitney *U* test (**E**–**H**).

Treg cells provide tissue protection following acute lung injury due to influenza virus infection and other causes of lung pathology and are necessary for resolution of inflammation and repair of lung injury during recovery (6, 32–35). AMPKα1 is required for bulk CD4^+^ T cell expansion in the lung during viral pneumonia (36), but whether AMPKα1/α2 are necessary for Treg cell function in this context is unknown. We found that AMPKα1/α2–sufficient Treg cells express both *Prkaa1* and *Prkaa2* in lymph nodes and the lung during viral pneumonia (Supplemental Figure 7, A and B) and that steady-state splenic AMPKα1/α2–deficient Treg cells have downregulated expression of genes activated during influenza A virus infection (37) (Figure 4A). Considering this transcriptional signature, our findings in the B16 melanoma model, and the similarity in interstitial fluid metabolite abundance between flu IF and tumor IF relative to plasma, we hypothesized that Treg cell–specific loss of AMPKα1/α2 would compromise protection from severe viral pneumonia. To test our hypothesis, we challenged *Prkaa1/2^fl/fl^Foxp3^YFP–Cre^* and control mice with intratracheal inoculations of influenza virus. *Prkaa1/2^fl/fl^Foxp3^YFP–Cre^* mice experienced higher mortality, greater weight loss throughout the disease course, and worsened hypoxemia (Figure 4, B–D), consistent with a loss of Treg cell tissue-protective function. Accordingly, we detected a significantly greater absolute number of lung CD45^+^ and CD8^+^ Tconv cells in *Prkaa1/2^fl/fl^Foxp3^YFP–Cre^* mice relative to controls at day 10 after influenza virus inoculation (Figure 4, E and F); lung Treg and CD4^+^ Tconv cell absolute counts were not significantly different between groups (Figure 4, G and H). The CD8^+^ Tconv cell compartment displayed a nominal shift away from an effector phenotype in the lungs of *Prkaa1/2^fl/fl^Foxp3^YFP–Cre^* mice, but no significant differences were detected in the proportion of other lung CD8^+^ and CD4^+^ T cell subsets, including Treg cells (Supplemental Figure 7, C–J). AMPKα1/α2–deficient Treg cells were less proliferative in the lung at day 10 after influenza virus inoculation according to Ki-67 expression relative to control Treg cells (Supplemental Figure 7K). Nevertheless, like in our malignancy model, AMPKα1/α2 deficiency did not alter Treg cell *Foxp3*/FOXP3 gene/protein expression or the levels of markers associated with Treg cell suppressive function (Supplemental Figure 7, L–R).

As AMPK is a known regulator of cellular metabolism, we assessed the metabolic state of AMPKα1/α2–deficient lung Treg cells during viral pneumonia by performing LC-MS on AMPKα1/α2–sufficient and –deficient Treg cells sorted from lungs at day 10 after influenza virus inoculation. A total of 159 annotated metabolites were identified (Figure 4, I and J, and Supplemental Data Set 8), revealing an enrichment of pyruvic acid and lactic acid in AMPKα1/α2–deficient lung Treg cells (Figure 4, K and L), metabolites that are upstream of the tricarboxylic acid (TCA) cycle, suggestive of altered mitochondrial metabolism. We also detected depletion of glutathione (GSH), a key antioxidant, in AMPKα1/α2–deficient lung Treg cells (Figure 4M). Overrepresentation analysis of the significantly differentially represented features revealed an overrepresentation of metabolites relating to glycine, serine, and threonine metabolism, glutathione metabolism, and pyruvate metabolism in AMPKα1/α2–deficient Treg cells (Figure 4N). Considering the small number of differentially represented metabolites at homeostasis (15 in total; see Supplemental Figure 1, M and N), these data suggest AMPK is necessary for Treg cell metabolic adaptation and function during influenza virus pneumonia-induced lung injury.

### Individual loss of AMPKα2 or AMPKα1 does not compromise Treg cell tissue-protective function during lung injury from viral pneumonia.

Considering the dichotomous consequence that individual loss of AMPKα1 versus loss of AMPKα2 had for tumor-infiltrating Treg cell–suppressive function, we also evaluated how each AMPKα subunit contributed to Treg cell function during viral pneumonia by challenging *Prkaa1^fl/fl^Foxp3^YFP–Cre^*, *Prkaa2^fl/fl^Foxp3^YFP–Cre^*, and control mice with intratracheal inoculations of influenza virus. Survival, weight change, and arterial blood oxygenation over time were similar across all 3 groups (Supplemental Figure 8, A–C). At day 10 after inoculation, loss of AMPKα2 resulted in significantly greater proportions of central memory CD8^+^ T cells and effector Treg cells but not the frequency of naive and effector CD8^+^ T cells, the frequency of naive and effector CD4^+^ Tconv cells, the frequency of Treg cells out of total CD4^+^ cells, or the frequency of central or proliferating Treg cells (Supplemental Figure 8, D–L). While lung Treg cells in *Prkaa2^fl/fl^Foxp3^YFP–Cre^* mice displayed significantly lower proliferation, TIGIT protein expression, and frequency of ICOS^Hi^ Treg cells, the Treg cell protein expression of FOXP3, CD25, PD-1, and CTLA-4 were similar across groups (Supplemental Figure 8, M–R). Considering the expression of *Prkaa1* and *Prkaa2* in control Treg cells of influenza-infected lungs (Supplemental Figure 7, A and B), as well as the compromised tissue-protective function of AMPKα1/α2–deficient Treg cells during viral pneumonia, these data suggest that AMPKα1 and AMPKα2 share redundant functions in this context.

### AMPKα is necessary for maximal mitochondrial function in Treg cells.

**Figure 5:**
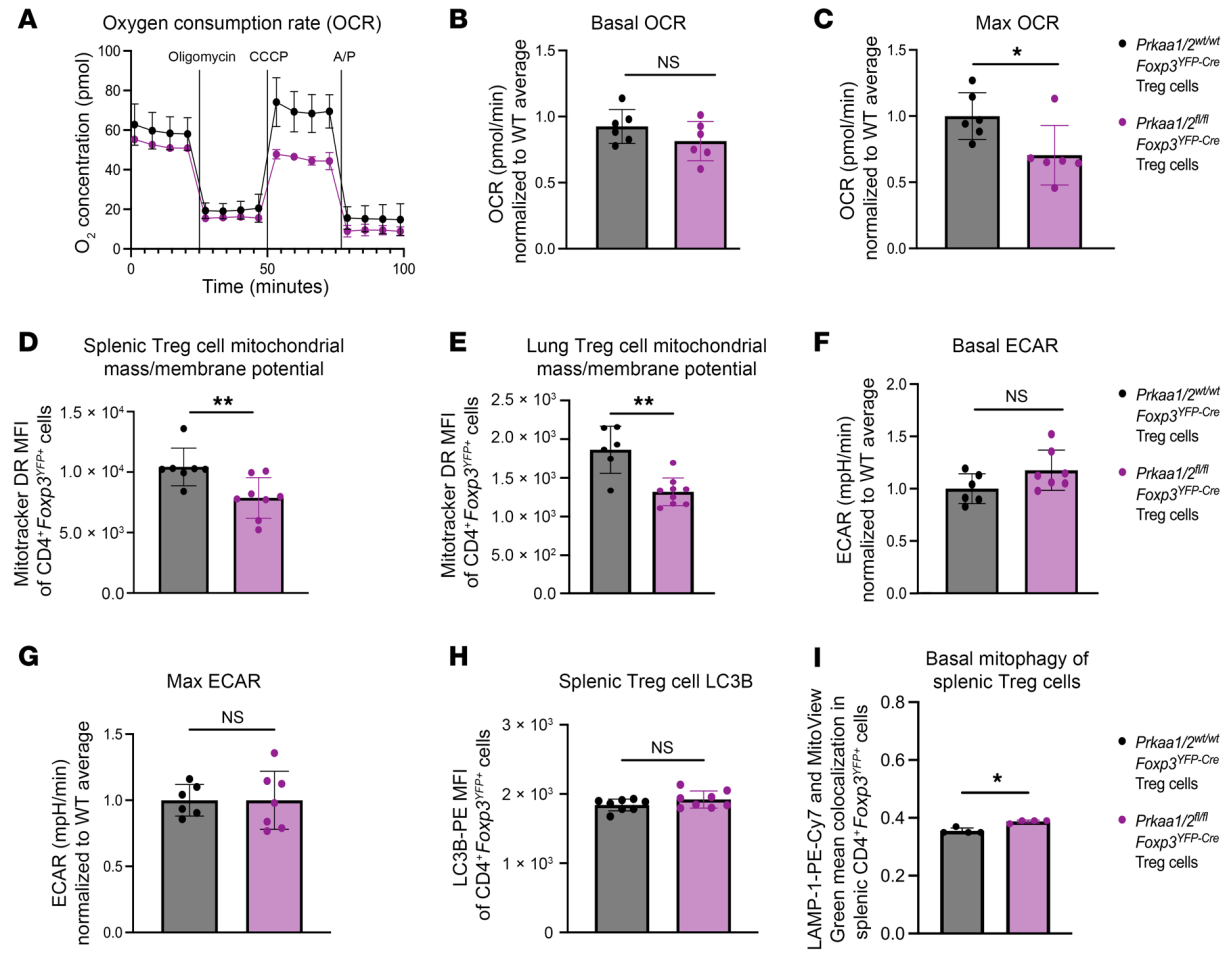
AMPKα is necessary for maximal Treg cell mitochondrial function. (**A**) Representative oxygen consumption rate (OCR) over time of CD4^+^*Foxp3*^YFP+^ splenocytes from *Prkaa1/2^wt/wt^Foxp3^YFP–Cre^* (control, *n* = 3) and *Prkaa1/2^fl/fl^Foxp3^YFP–Cre^* (*n* = 2) mice following treatment of oligomycin (2.5 μM), carbonyl cyanide m-chlorophenylhydrazone (CCCP; 10 μM), and antimycin A/piercidin (A/P; 2 μM each), as measured by a metabolic flux assay. (**B**–**C**) Basal (**B**) and maximal (**C**) OCR of CD4^+^*Foxp3*^YFP+^ splenocytes from control (*n* = 6) and *Prkaa1/2^fl/fl^Foxp3^YFP–Cre^* (*n* = 6) mice, some of which are shown in **A**. (**D** and **E**) MitoTracker Deep Red (MitoTracker DR) mean fluorescence intensity (MFI) of CD4^+^*Foxp3*^YFP+^ splenocytes at homeostasis (**D**; *n* = 7 control, *n* = 8 *Prkaa1/2^fl/fl^Foxp3^YFP–Cre^* mice) and lung CD4^+^*Foxp3*^YFP+^ cells at day 10 after influenza virus inoculation (**E**; same cohort as in Figure 4, E–H, and Supplemental Figure 7, *n* = 6 control, *n* = 9 *Prkaa1/2^fl/fl^Foxp3^YFP–Cre^* mice). (**F** and **G**) Basal (**F**) and maximal (**G**) extracellular acidification rate (ECAR) of CD4^+^*Foxp3*^YFP+^ splenocytes from control (*n* = 6) and *Prkaa1/2^fl/fl^Foxp3^YFP–Cre^* (*n* = 7) mice. (**H**) LC3B-PE MFI of CD4^+^*Foxp3*^YFP+^ splenocytes from control (*n* = 8) and *Prkaa1/2^fl/fl^Foxp3^YFP–Cre^* (*n* = 8) mice. (**I**) Mean LAMP-1-PE-Cy7 and MitoView Green colocalization in CD4^+^*Foxp3*^YFP+^ splenocytes from control (*n* = 4) and *Prkaa1/2^fl/fl^Foxp3^YFP–Cre^* (*n* = 4) mice. *P < 0.05, **P < 0.01, NS, not significant according to Mann-Whitney *U* test.

We demonstrated that mitochondrial metabolism, specifically activity of the electron transport chain, is a key determinant of Treg cell suppressive function (10). To test whether loss of AMPK compromises Treg cell mitochondrial function, we assessed the metabolic status of AMPKα1/α2–deficient and control Treg cells with a metabolic flux assay, finding that AMPKα1/α2–deficient Treg cells have comparable basal oxygen consumption rates (OCR) but significantly lower maximum OCR relative to control Treg cells (Figure 5, A–C). In fact, AMPKα1/α2–deficient Treg cells were unable to augment their OCR above baseline when challenged with the mitochondrial uncoupling agent carbonyl cyanide m-chlorophenylhydrazone (CCCP). Staining with MitoTracker Deep Red (MitoTracker DR, a dye used to measure mitochondrial mass that is sensitive to mitochondrial membrane potential) revealed lower mitochondrial mass/membrane potential in AMPKα1/α2–deficient Treg cells relative to controls at homeostasis (Figure 5D) and in the lung during influenza pneumonia (Figure 5E), consistent with their impaired maximal OCR at homeostasis. AMPK also promotes glycolysis (38); however, the baseline and maximal extracellular acidification rate (ECAR, a measure of glycolytic rate) of AMPKα1/α2–deficient and control Treg cells was comparable between genotypes, suggesting that AMPK is not required to sustain glycolysis in Treg cells at homeostasis (Figure 5, F and G). When assessing the individual contribution of the two AMPKα isoforms to mitochondrial mass and metabolism, we found that AMPKα1-deficient Treg cells and AMPKα2-deficient Treg cells had a trend toward lower basal OCR but significantly lower mitochondrial mass in the absence of a basal glycolytic rate defect (Supplemental Figure 9, A–C). AMPK also promotes autophagy through inhibition of mammalian target of rapamycin complex 1 (mTORC1) (39, 40), yet we found by flow cytometry that AMPKα1/α2–deficient Treg cells had no significant differences in protein expression of the autophagy marker LC3B (Figure 5H). We further assessed autophagy in splenic AMPKα1/α2–deficient and control Treg cells by measuring colocalization of lysosomal-associated membrane protein 1 (LAMP1) and mitochondria using a LAMP1 fluorochrome and MitoView Green, respectively, as a readout of mitophagy using imaging flow cytometry. We found that AMPKα1/α2–deficient Treg cells had a minimal but significant increase in the colocalization of LAMP-1 and mitochondria, consistent with a nominally significantly greater mitophagy in AMPKα1/α2–deficient Treg cells (Figure 5I). To validate this method for measuring mitophagy, we treated AMPK-sufficient splenic Treg cells with CCCP, the mitochondrial decoupling agent frequently used to induce mitophagy (one of many forms of autophagy) in mammalian cells (41) and measured the change in anti-LAMP-1 fluorochrome mean fluorescence intensity (MFI), MitoView Green MFI, and the mean colocalization between the anti-LAMP-1 fluorochrome and MitoView Green. CCCP treatment decreased the MitoView Green MFI (Supplemental Figure 9D) while increasing the colocalization between anti-LAMP-1 fluorochrome and MitoView Green over time (Supplemental Figure 9E), consistent with an upregulation of mitophagy in the setting of prolonged mitochondrial decoupling. These results collectively suggest that both AMPKα subunits contribute to, and are required for, maximal mitochondrial mass and electron transport chain function in Treg cells.

### AMPKα regulates DNMT1 to promote demethylation of metabolic genes.

**Figure 6:**
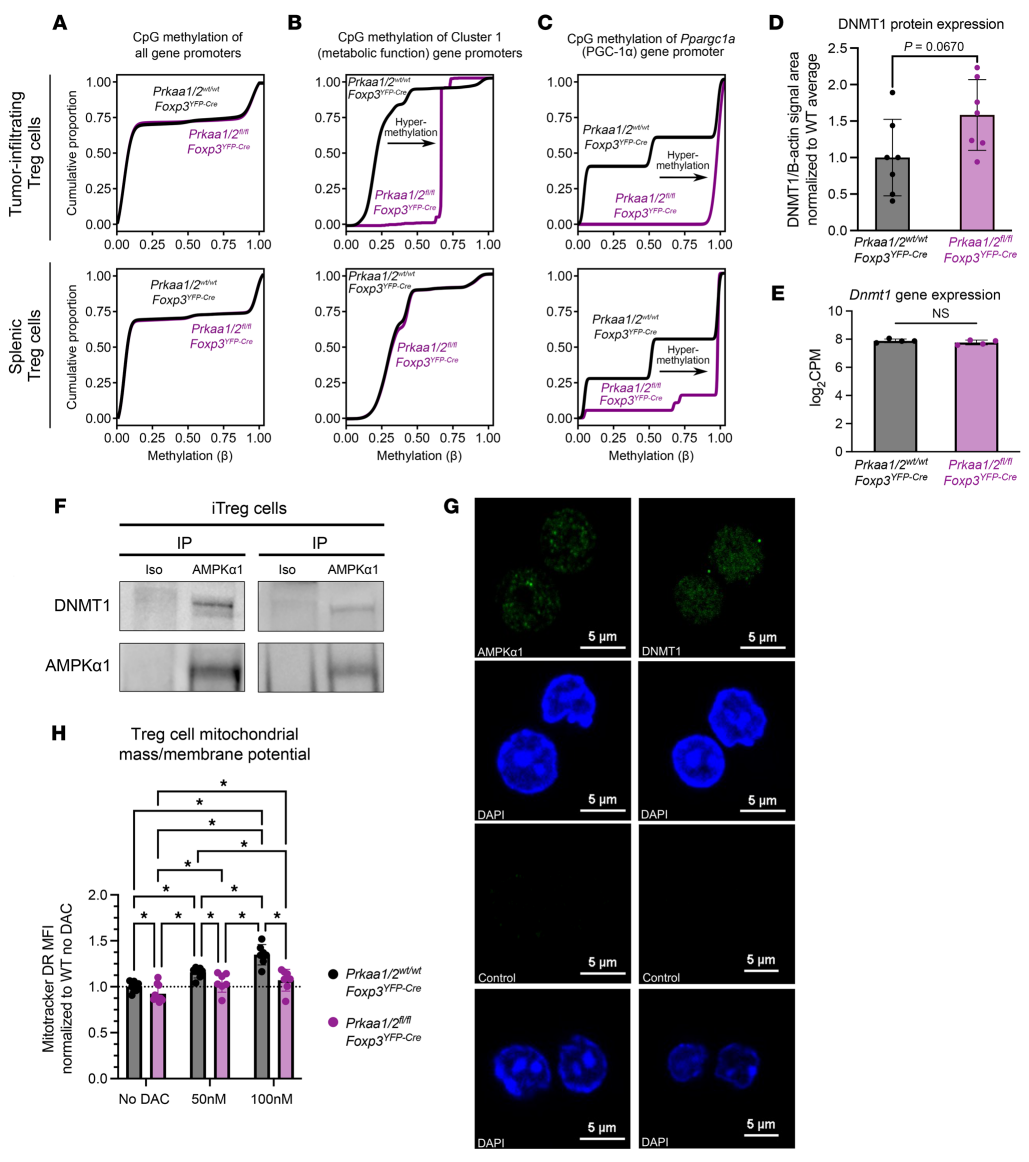
AMPKα1 interacts with DNMT1 to demethylate the promoter of mitochondrial genes in tumor-infiltrating Treg cells. (**A**–**C**) CpG methylation of all gene promoters (**A**), gene promoters of cluster 1 genes identified by *k*-means clustering of the RNA-seq shown in Figure 2E (**B**), and the *Ppargc1a* promoter (**C**) in tumor-infiltrating CD4^+^*Foxp3*^YFP+^ cells (*n* = 4 *Prkaa1/2^wt/wt^Foxp3^YFP–Cre^* or control, *n* = 2 *Prkaa1/2^fl/fl^Foxp3^YFP–Cre^*) and splenic CD4^+^*Foxp3*^YFP+^ cells at homeostasis (*n* = 3 control, *n* = 3 *Prkaa1/2^fl/fl^Foxp3^YFP–Cre^*) (**D**) DNMT1 protein expression of splenic CD4^+^*Foxp3*^YFP+^ (Treg) cells at homeostasis (*n* = 7 control, *n* = 7 *Prkaa1/2^fl/fl^Foxp3^YFP–Cre^*). 3 independent experiments are shown. DNMT1 peak intensity area was normalized to the corresponding sample’s β-actin peak intensity area. (**E**) *Dnmt1* gene expression of splenic CD4^+^*Foxp3*^YFP+^ cells at homeostasis (*n* = 4 control, *n* = 4 *Prkaa1/2^fl/fl^Foxp3^YFP–Cre^*) as measured by RNA-seq shown in Figure 1. (**F**) Anti-AMPKα1 and isotype control immunoprecipitates from ex vivo induced (i)Treg cell lysates blotted for DNMT1 protein. Independent biological replicates are shown. (**G**) Representative microscopy images of AMPKα-sufficient iTreg cells showing AMPKα1 and DNMT1 subcellular localization. Scale bars: 5 μm. (**H**) MitoTracker Deep Red (MitoTracker DR) mean fluorescence intensity (MFI) of AMPKα-sufficient (control) and -deficient splenic CD4^+^*Foxp3*^YFP+^ cells treated with either vehicle (*n* = 8 control, *n* = 10 *Prkaa1/2^fl/fl^Foxp3^YFP–Cre^*), 50 nM decitabine (DAC, *n* = 7 control, *n* = 7 *Prkaa1/2^fl/fl^Foxp3^YFP–Cre^*), or 100 nM DAC (*n* = 7 control, *n* = 7 *Prkaa1/2^fl/fl^Foxp3^YFP–Cre^*). *P or *q* < 0.05, NS, not significant according to Mann-Whitney *U* test (**D** and **E**) with 2-stage linear step-up procedure of Benjamini, Krieger, and Yekutieli with Q = 5% (**H**).

In human umbilical vein endothelial cells and mesenchymal stem cells cultured in vitro, AMPK phosphorylates DNMT1 to promote transcription of metabolic genes, including *Ppargc1a* (17, 18). Hence, we hypothesized that the lower expression of metabolic genes by tumor-infiltrating AMPKα1/α2–deficient Treg cells (see Cluster 1 in Figure 2, E–H) was a consequence of DNA hypermethylation at their gene promoters. We tested this hypothesis by performing genome-wide DNA methylation profiling of *Prkaa1/2^fl/fl^Foxp3^YFP–Cre^* and control Treg cells sorted from B16 melanoma tumors and from spleens at homeostasis. While there was no difference in genome-wide promoter methylation, we observed hypermethylation of Cluster 1 (Figure 2E) gene promoters in AMPKα1/α2–deficient tumor-infiltrating Treg cells, as well as hypermethylation of *Ppargc1a* (PGC-1α) in tumor-infiltrating and splenic AMPKα1/α2–deficient Treg cells (Figure 6, A–C). While we found that AMPKα1/α2–deficient splenic Treg cells exhibited a trend (*P* = 0.067) toward higher DNMT1 protein levels relative to controls, they had no differences in *Dnmt1* gene expression (Figure 6, D and E). AMPKα1-deficient Treg cells exhibited similar, and AMPKα2-deficient Treg cells a trend toward lower, DNMT1 protein expression relative to controls, respectively (Supplemental Figure 10A). Coimmunoprecipitation assays in primary mouse iTreg cells, Jurkat cells, and the Treg cell–like MT-2 cell line (42, 43) identified a physical interaction between AMPKα1 and DNMT1 (Figure 6F and Supplemental Figure 10, B and C). To determine whether AMPK is present in the nucleus where it can interact with DNMT1, we performed immunofluorescence imaging in iTreg cells as well as Jurkat and FOXP3^+^ MT-2 cells. Consistent with their physical interaction, our imaging studies identified AMPKα1 in the nucleus (Figure 6G and Supplemental Figure 10, D and E). The subcellular compartmentalization of AMPKα1 was unaffected by activation with 5-aminoimidazole-4-carboxamide ribonucleoside (AICAR) in MT-2 cells (Supplemental Figure 10F). Finally, we established the functional relevance of these findings by demonstrating that inhibition of DNMT activity with decitabine (DAC) — a clinically used agent we showed in published work promotes Treg cell function and is sufficient to induce DNA hypomethylation in Treg cells (33) — increased MitoTracker DR staining (mitochondrial mass/membrane potential) in AMPKα1/α2-sufficient splenic Treg cells in a dose-dependent manner (Figure 6H). Treatment of AMPKα1/α2–deficient Treg cells with DAC also rescued MitoTracker DR signal to that of untreated control Treg cells, confirming that DNA methylation regulates mitochondrial mass in AMPKα1/α2–deficient Treg cells. Altogether, these experimental data reveal AMPK as a nuclear factor that regulates DNMT1 in Treg cells to promote expression of metabolic factors that potentiate mitochondrial metabolism.

## Discussion

Treg cells exhibit metabolic plasticity in the TME, which, in turn, supports Treg cell suppressive function (12, 13). Nevertheless, mechanisms that orchestrate the metabolic adaptation of tumor-infiltrating Treg cells remain undetermined. Here, our experimental data revealed that AMPK-deficient Treg cells failed to exert optimal suppressive function in metabolically stressed microenvironments. We found that AMPK-deficient Treg cells were unable to augment their oxygen consumption under the stress of a mitochondrial uncoupling agent ex vivo, failed to upregulate genes supporting mitochondrial metabolism in the TME, and did not sustain proper mitochondrial mass/membrane potential or metabolic homeostasis during viral pneumonia. These results credit AMPK as a key mediator of Treg cell metabolic adaptation to settings of microenvironmental stress, likely through potentiation of mitochondrial metabolism, and are consistent with in vitro experiments suggesting that AMPK potentiates Treg cell suppressive function (44).

While comparison of the metabolomic profiles of the TME and virus-injured lung interstitial fluid showed similar alterations when compared with plasma, we observed differences in the abundance of a small set of metabolites between these two interstitial fluid compartments, including glucose and glutamine, which were lower in the TME compared with the lung during viral pneumonia. In most cell types, increases in the AMP-to-ATP ratio from glucose deprivation and other states of energy stress lead to phosphorylation of AMPK by liver kinase B1 (LKB1) (45). Notably, Treg cells require LKB1 to sustain immune self tolerance at homeostasis, albeit in an AMPK signaling–independent manner (19, 21). Cell signaling events such as T cell receptor (TCR) engagement also activate AMPK via calcium/calmodulin-dependent protein kinase kinase (CaMKK) (16, 46). These AMPK-activating events likely contribute to the metabolic adaptation mediated by Treg cell AMPK in disease microenvironments and serve as independent inputs through which AMPK can sense and respond to the extracellular milieu. Therefore, it is plausible that the loss-of-function we observed in tumor-infiltrating AMPK-deficient Treg cells is driven by an inability to adapt to glucose or other nutrient deprivation, whereas lung Treg cells require AMPK during influenza to adapt to different metabolic and signaling challenges. It remains unclear what dimensions of Treg cell function are lost in AMPK-deficient Treg cells in these microenvironments, as our measurements of classical surface molecules via which Treg cells exert their suppressive function did not reveal broad changes in Treg cell suppressive phenotype. Nevertheless, we detected greater IFN-γ production in intratumoral CD8^+^ T cells in mice with Treg cell–specific AMPK deficiency, indicating a loss of classical suppressive function.

Our experimental data suggest that AMPK regulates DNMT1 to activate the expression of metabolic genes that support mitochondrial function, including *Ppargc1a*/PGC-1α. In some cell types cultured in vitro, an AMPK-DNMT1-mitochondrial metabolism axis regulates metabolic function (17, 18). In vivo, we found in tumor-infiltrating Treg cells that AMPK serves as an epigenetic regulator of transcriptional programs that support metabolic function and the Treg cell lineage. Loss of AMPK in Treg cells led to DNA hypermethylation at the promoters of key metabolic genes in the TME. Our coimmunoprecipitation studies confirmed that AMPKα1 directly interacts with DNMT1, likely regulating DNMT1 activity via phosphorylation events suggested by in vitro studies (17, 18). Critically, treatment with the DNMT inhibitor decitabine rescued mitochondrial mass in AMPK-deficient Treg cells with a dose-response correlation that was steeper in AMPK-sufficient compared with -deficient cells, mechanistically connecting AMPK, DNMT1, and mitochondria. These findings are consistent with the higher degree of DNA methylation present in AMPK-deficient cells, making them relatively more resistant to the effect of DNA methyltransferase inhibition. Additional mechanisms may link AMPK to DNA methylation writer complexes. For example, UHRF1, the nonredundant DNMT1 adapter protein we previously showed to be necessary for Treg cell identity and function (23), has been reported to inhibit AMPK function in the nucleus of hepatocytes (47). Hence, AMPK may regulate DNA methylation in Treg cells via interaction with other DNMT complex members such as UHRF1. Finally, multifactorial mechanisms induce and regulate FOXP3 expression in harsh settings such as the TME (48–50). While we found a nominally lower level of FOXP3 protein expression in AMPK-deficient compared with AMPK-sufficient Treg cells at baseline, this difference was not evident in the tumor and infected lung microenvironments, indicating that the mechanisms controlling FOXP3 level in these settings are not dependent on AMPK. Interestingly, we observed lower *Foxp3* gene expression by RNA-seq in AMPK-deficient Treg cells isolated from the TME at day 12 but not at day 15, suggesting important regulatory events occurring over the course of tumor growth.

The context-specific upregulation of *Prkaa2* in tumor-infiltrating Treg cells may explain the discrepant consequences for antitumor immunity reported in Treg cell–specific AMPKα1-deficient mice challenged with B16 melanoma tumors (29, 30). A study assessing the contribution of each AMPK catalytic subunit isoform to the potentiation of mitochondrial gene expression found that AMPKα2, but not AMPKα1, is required for the upregulation of *Ppargc1a* expression during myotube differentiation (51). While we showed that AMPKα1 also interacts with DNMT1 in T cell lines and primary FOXP3^+^ T cells, it is plausible that the two AMPKα isoforms exert differential regulation over epigenetic modifiers in the TME. Therefore, *Prkaa2* upregulation by tumor-infiltrating, AMPKα1-deficient Treg cells may impact Treg cell suppressive function and thereby lead to conflicting results, especially if *Prkaa2* upregulation is modified by variables that are difficult to control across studies, such as the mouse colony microbiome (52). Indeed, our data suggest that, while AMPKα1 and AMPKα2 may have a shared set of downstream targets that are necessary for Treg cell function in the TME and the lung during viral pneumonia, isoform-specific activities may have divergent influences on Treg cell function, as evidenced by the dichotomous tumor burden relative to control mice observed in *Prkaa1^fl/fl^Foxp3^YFP–Cre^* and *Prkaa2^fl/fl^Foxp3^YFP–Cre^* mice.

Clinical trial data suggest that metformin, an indirect AMPK activator, significantly reduces the risk of developing long COVID (53–55). This finding is consistent with the observed effects of metformin on mouse models of lung injury (56, 57). Our data support that AMPK is dispensable for Treg cell-mediated immune self tolerance yet promotes Treg cell suppressive function in disease microenvironments. This context-specific requirement of AMPK for Treg cell function makes it an attractive drug target for attempts to potentiate the function of Treg cells ex vivo before their use in cell-based therapies, such as those being leveraged in early phase clinical trials to improve outcomes in patients with COVID-19 (58, 59).

Our study has limitations. First, AMPK phosphorylates specific residues of DNMT1 in human umbilical vein endothelial cells to decrease DNMT1 activity (17). Unfortunately, antibodies specific for the homologous residues of mouse DNMT1 are not available. Regardless, our coimmunoprecipitation, immunofluorescence, immunoassay, and sequencing data support that AMPK regulates DNMT1 in Treg cells. Second, we detected 159 metabolites via LC-MS in approximately 5 × 10^4^ Treg cells sorted from the influenza virus–injured lung at peak injury. While we were able to detect an accumulation of pyruvic acid and lactic acid in AMPK-deficient Treg cells suggestive of an impaired TCA cycle, a more comprehensive assessment of the Treg cell metabolome during viral pneumonia may have provided insight into whether the loss-of-function in this context is due to energy stress in the absence of AMPK-mediated metabolic adaptation. Finally, the loss of AMPK-dependent regulation of transcriptomic and epigenetic signatures may be too complex to cause the resulting Treg cell loss-of-function via a single factor, such as dampened *Ppargc1a* expression; the combined dysregulation of more than a single downstream target of AMPK is likely to mediate the loss of function.

In summary, our findings support a model in which AMPK coordinates the metabolic adaptation of Treg cells in settings of microenvironmental stress by potentiating mitochondrial metabolism, consistent with AMPK’s canonical function as a sensor of energetic stress and the central role mitochondrial metabolism plays in programming Treg cell functional state. We show that this AMPK-mediated metabolic adaptation is executed in part through the regulation of DNA methylation at key metabolic loci, offering potential pharmacologic targets to modulate Treg cell function in disease, including in severe lung injury and cancer.

## Methods

### Sex as a biological variable.

Sex was not considered as a biological variable in all experiments. See Supplemental Methods for further details.

### Mice.

*Prkaa1^fl/fl^* (cat. no. 014141), *Prkaa2^fl/fl^* (cat. no. 014142), and *Foxp3^YFP–Cre^* (cat. no. 016959) mice from the C57BL/6J genetic background were purchased from The Jackson Laboratory. All animals were genotyped using services provided by Transnetyx Inc., with primers provided by The Jackson Laboratory and shown in Supplemental Table 1. Animals received water ad libitum, were housed at a temperature range of 20°C–23°C under 14-hour light/10-hour dark cycles and received standard rodent chow. See Supplemental Methods for further details.

### Flow cytometry and cell sorting.

Single-cell suspensions of organ tissues, blood, tumors, or cultured cells were prepared and stained for flow cytometry analysis and sorting, as previously described (23, 34) using the reagents shown in Supplemental Table 2. See Supplemental Methods for further details.

### Imaging flow cytometry measurement of mitophagy.

Splenic single cell suspensions were stained with surface markers and MitoView Green (20 nM) and treated with 10 μM CCCP for 30, 60, 120, and 180 minutes to induce mitophagy. Samples were then fixed as above. Fixed single cell suspensions were then stained with anti-LAMP1 and anti-FOXP3 antibodies at 4°C for 30 minutes. Imaging flow cytometry was performed using a BD FACS Discover S8 cell sorter and analyzer and the subcellular colocalization of LAMP1 and MitoView Green signal was assessed using BD CellView Image Technology in the BD FACSChorus software.

### iTreg cell induction and culture.

iTreg cells were induced and cultured as previously described (23). See Supplemental Methods for further details.

### B16 melanoma tumor model.

B16-F10 cells (ATCC CRL-6475) were cultured as previously described (23). 250,000 B16-F10 cells were resuspended in 0.1 mL of PBS and 40% Matrigel (Corning cat. no. 356237) and injected subcutaneously in the hair-trimmed flanks of 12–15 week-old mice. See Supplemental Methods for further details.

### Influenza A virus administration.

Mice were anesthetized with isoflurane and intubated using a 20-gauge angiocatheter cut to a length that placed the tip of the catheter above the carina. Mice were instilled with mouse-adapted influenza A/WSN/33 [H1N1] virus (12.5 plaque-forming units in 50 μL of sterile PBS) as previously described (34).

### Measurement of physiologic readouts of influenza pneumonia progression and resolution.

Arterial blood oxygen saturation (SpO_2_) was measured in control and influenza virus–infected mice using a MouseOx Plus pulse oximeter (Starr Life Sciences). Beginning on the fifth day after inoculation and continuing every other day, SpO_2_ was measured with oximeter collar clips secured to the hairless neck of conscious, immobilized animals. Mouse weights were recorded the day of influenza virus inoculation and every other day after inoculation starting on day 5. Mouse weights were normalized to those recorded on the day of inoculation.

### Lung tissue harvesting and processing.

These procedures have been previously reported (34). See Supplemental Methods for further details.

### Immunoblotting.

Cultured cells were lysed for 1 hour at 4°C in lysis buffer (Cell Signaling cat. no. 9803) supplemented with phosphatase (Cell Signaling cat. no. 5870S) and protease inhibitors (Roche, cat. no. 65726900) after which their concentration was measured with a BCA assay according to manufacturer instructions (Pierce cat. no. 23225). Cell lysates were subjected to gel electrophoresis and transferred to membranes that were incubated with an antibody against AMPKα1 (Abcam cat. no. ab32047), DNMT1 (Cell Signaling cat. no. 5032), and β-actin (Abcam cat. no. ab8227) overnight at 4°C with constant agitation.

### Wes protein immunoassay.

Flow cytometry–sorted cells were lysed, and the resulting lysate protein concentrations were measured as described above. For protein measurements using the Simple Wes immunoassay system, 0.5 μg of protein in 3 μL were loaded per well and processed according to the manufacturer’s instructions. The following concentrations were used for primary antibodies: 1:50 anti-DNMT1 (Invitrogen cat. no. MA5-16169), 1:50 anti-AMPKα (Cell Signaling cat. no. 2532S), and 1:50 anti-β-actin (Abcam cat. no. ab8227).

### Coimmunoprecipitation assay.

1 × 10^6^ cells were lysed in cell lysis buffer for 1 hour at 4°C as described above. Lysates were incubated with an antibody against AMPKα1 (Abcam cat. no. ab32047) or isotype control (Cell Signaling cat. no. 7074) overnight at 4°C with constant agitation. The immune complex was precipitated with Dyna Protein G beads (Life Technologies cat. no. 10003D), washed, and resuspended in SDS/PAGE loading buffer, and heated to 95°C for 5 minutes. Processed samples were then blotted with antibodies against DNMT1 (Cell Signaling cat. no. 5032), AMPKα1 (Abcam cat. no. ab32047), and β-actin (Abcam cat. no. ab8227). Jurkat cells were obtained from ATCC. MT-2 cells were a gift from Jason R. Mock (University of North Carolina, Chapel Hill, North Carolina, USA).

### Immunofluorescence for microscopy.

1 × 10^6^ cells were fixed with ice-cold 100% methanol for 5 minutes. Subsequently, samples were processed with Immunofluorescence Application Solutions Kit (Cell Signaling cat. no. 12727) following the manufacturer’s protocol. Cells were stained overnight at 4°C with anti-DNMT1 (Abcam cat. no. ab21799 1), anti-AMPKα1 (Abcam cat. no. ab32047), Alexa fluor 488-conjugated isotype control (Abcam cat. no. ab199091), or unconjugated isotype control (Abcam cat. no. ab172730). The following day, cells that were stained with anti-AMPKα1 antibody and unconjugated isotype control antibody were incubated in the dark at room temperature for 2 hours with anti-rabbit Alexa Fluor 488 secondary antibody (Abcam cat. no. ab150113). Following antibody incubation, cells were mounted on a slide with VECTASHIELD Vibrance mounting medium containing DAPI (Vector Labs cat. no. H-1800). Fluorescent images were acquired at room temperature using a confocal microscope (Nikon) with 40× magnification at the Northwestern Center for Advanced Microscopy.

### Nuclear-cytoplasmic fractionation assay.

5 × 10^6^ iTREG cells were treated and subsequently underwent lysis using NE-PER Nuclear and Cytoplasmic Extraction kit (Thermo Fisher Scientific cat. no. 78833) according to the manufacturer’s protocol. Nuclear and cytoplasmic fractions were collected and further analyzed for the expression of proteins of interest with immunoblotting as described above.

### Metabolic flux (Seahorse) assay.

2.5 × 10^5^ flow cytometry-sorted Treg cells were seeded on a 96-well Seahorse cell culture plate and analyzed on a Seahorse XF24 Analyzer (10). The following drugs and corresponding doses were loaded onto ports A, B, C, and D in the same order: oligomycin (2.5 μM, Sigma-Aldrich cat no. 75351), CCCP (10 μM, Sigma-Aldrich cat no. C2759), antimycin A/piericidin A (2 μM each, Sigma-Aldrich cat no. A8674 and 15379, respectively), and 2-deoxyglucose (25 mM, Sigma-Aldrich cat no. D8375).

### RNA-seq, modified reduced representation bisulfite sequencing (mRRBS) and analysis.

Nucleic acid isolation and next-generation sequencing library preparation was performed using custom procedures previously described by our group (23, 34, 60). RNA-seq and mRRBS analysis was performed using previously published procedures (61). See Supplemental Methods for further details.

### Collection of lung Treg cells for metabolomics.

Lung single-cell suspensions were subjected to CD4^+^ cell positive enrichment according to kit manufacturer’s instructions (Miltenyi Biotec cat. no. 130-097-048) before fluorochrome staining. Using a MACSQuant Tyto, 5–10 × 10^5^ lung Treg cells were sorted from each pair of lungs. Sorted cells were centrifuged at 500*g* for 6 minutes at 4°C. Pelleted cells were resuspended in 15 μL of 80% acetonitrile and vortexed for 30 seconds. Following centrifugation for 30 minutes at 20,000*g* at 4°C, the supernatant was collected for LC-MS. See Supplemental Methods for further details.

### Collection of interstitial fluid and plasma for metabolomics.

Blood was centrifuged at 800*g* for 10 minutes at 4°C in EDTA tubes. The plasma phase was pipetted, frozen with liquid nitrogen, and stored at –80°C. Intact tumors and lungs were centrifuged at 100*g* for 10 minutes at 4°C in centrifuge tubes containing a 0.22 μm filter (Costar cat. no. 8160). The extracted interstitial fluid was then diluted 1-to-5 in 80% acetonitrile and vortexed for 30 seconds. The diluted interstitial fluid was centrifuged for 30 minutes at 20,000*g* at 4°C and the supernatant was collected for LC-MS analysis. See Supplemental Methods for further details.

### HPLC and high-resolution mass spectrometry and LC-MS for metabolomics.

The system consisted of a Thermo Q-Exactive in line with an electrospray source and an Ultimate3000 (Thermo Fisher Scientific) series HPLC consisting of a binary pump, degasser, and auto-sampler outfitted with a Xbridge Amide column (Waters; dimensions of 3.0 mm × 100 mm and a 3.5 μm particle size). Data acquisition and analysis were carried out by Xcalibur 4.1 software and Tracefinder 4.1 software, respectively (both from Thermo Fisher Scientific). See Supplemental Methods for further details.

### LC-MS data analysis.

Raw peak intensity data of the metabolites detected by LC-MS were uploaded to Metaboanalyst 5.0’s statistical analysis [1 factor] module. For comparisons with more than 2 groups, 1-way ANOVA with *q* < 0.05 was employed to identify significant differentially enriched metabolites. Comparisons with only 2 groups were analyzed with multiple parametric 2-tailed *t* tests and fold-change analysis using Metabonalyst 5.0’s standard settings (*P* < 0.1). Fold change threshold was set to 1.5 in resulting volcano plots to increase the power of the downstream overrepresentation analysis. See Supplemental Methods for further details.

### Statistics.

*P* values and FDR *q* values resulting from 2-tailed tests were calculated using statistical tests stated in the figure legends, including Mann-Whitney U test with or without the 2-stage linear step-up procedure of Benjamini, Krieger, and Yekutieli as specified in the figure legends, 2-way ANOVA with the 2-stage linear step-up procedure of Benjamini, Krieger, and Yekutieli, and log-rank (Mantel-Cox) test. using GraphPad Prism v10.1.0. Differences between groups with *P* or *q* values < 0.05 were considered statistically significant; see LC-MS data analysis for the statistical approach to metabolomic profiling data and RNA-seq, modified reduced representation bisulfite sequencing (mRRBS) and analysis for the statistical approach to transcriptomic and epigenomic profiling data. Using the ROUT method, the following number of outliers were excluded from the following figures: 1 from Figure 2B (Q = 0.5%), 2 from Figure 2C (Q = 0.5%), 4 from Supplemental Figure 2L (Q = 1.0%), and 3 from Supplemental Figure 4B (Q = 1.0%). Central tendency and error are displayed as mean ± SD except as noted. Box plots show median and quartiles. Numbers of biological replicates are stated in the figures or accompanying legends.

### Study approval.

All mouse procedures were approved by the Northwestern University IACUC under protocols IS00012519 and IS00017837.

### Data availability.

The raw and processed next-generation sequencing data sets were deposited in the NCBI’s Gene Expression Omnibus database (GEO GSE249019). Raw peak intensity data of annotated metabolites detected by LC-MS are available in the data supplement. All raw data is included in the Supporting Data Values file.

## Author contributions

MATA contributed to the conceptualization and methodology of this work; the data generation and visualization; the funding acquisition for this work; and to the writing and editing of the manuscript. JKG contributed to the generation of data and the editing of the manuscript. QL contributed to the methodology, generation and visualization of data, and the editing of the manuscript. NM contributed to the methodology, generation and visualization of data, and the editing of the manuscript. CRF contributed to the methodology, generation of data, and the editing of the manuscript. KAH contributed to the methodology, generation of data, and the editing of the manuscript. AMJ contributed to the generation of data and the editing of the manuscript. LMN contributed to the methodology, generation of data, and the editing of the manuscript. KC contributed to the generation of data and the editing of the manuscript. HAV contributed to the methodology, generation of data, and the editing of the manuscript. SEW contributed to the conceptualization and methodology of this work; the data generation and visualization; the supervision of this work; and to the writing and editing of the manuscript. BDS contributed to the conceptualization and methodology of this work; the data generation and visualization; the supervision, administration, and funding acquisition of this work; and to the writing and editing of the manuscript.

## Supplementary Material

Supplemental data

Supplemental data set 1

Supplemental data set 2

Supplemental data set 3

Supplemental data set 4

Supplemental data set 5

Supplemental data set 6

Supplemental data set 7

Supplemental data set 8

Unedited blot and gel images

Supporting data values

## Version 1 03/18/2025

In-Press Preview

## Version 2 05/01/2025

Electronic publication

## Version 3 09/18/2025

corrected copyright statement
