# Supplementary material for: AMPK is necessary for Treg functional adaptation to microenvironmental stress during malignancy and viral pneumonia
Source: J Clin Invest. 2025 Mar 18;135(9):e179572. doi: 10.1172/JCI179572 (PMC12043082; doi:10.1172/JCI179572)
Supplement: Supplemental data [file jci-135-179572-s007.pdf]

Supplement

AMPK is necessary for Treg functional adaptation to microenvironmental stress during malignancy and viral pneumonia

Manuel A. Torres Acosta[1,2,3], Jonathan K. Gurkan[1,2,3], Qianli Liu[1,3], Nurbek Mambetsariev[4], Carla P. Reyes Flores[1,3], Kathryn A. Helmin[1], Anthony M. Joudi[1], Luisa Morales-Nebreda[1], Kathleen Cheng[2,3,5], Hiam Abdala-Valencia[1], Samuel E. Weinberg[6,†], and Benjamin D. Singer[1,7,8,9,†,*]

[1]Division of Pulmonary and Critical Care Medicine, Northwestern University Feinberg School of Medicine, Chicago, IL USA

[2]Medical Scientist Training Program, Northwestern University Feinberg School of Medicine, Chicago, IL USA

[3]Driskill Graduate Program, Northwestern University Feinberg School of Medicine, Chicago, IL USA

[4]Division of Allergy and Immunology, Northwestern University Feinberg School of Medicine, Chicago, IL USA

[5]Department of Dermatology, Northwestern University Feinberg School of Medicine, Chicago, IL USA

[6]Department of Pathology, Northwestern University Feinberg School of Medicine, Chicago IL USA

[7]Department of Biochemistry and Molecular Genetics, Northwestern University Feinberg School of Medicine, Chicago, IL USA

[8]Simpson Querrey Institute for Epigenetics, Northwestern University Feinberg School of Medicine, Chicago, IL USA

[9]Simpson Querrey Lung Institute for Translational Science (SQ LIFTS), Northwestern University Feinberg School of Medicine, Chicago, IL USA.

[†] Senior authors contributed equally to this work.

*To whom correspondence should be addressed:

Benjamin D. Singer, MD

Division of Pulmonary and Critical Care Medicine, Department of Medicine

Department of Biochemistry and Molecular Genetics

Northwestern University Feinberg School of Medicine

303 E. Superior St.

Simpson Querrey 5th Floor

Chicago, IL 60611 USA

benjamin-singer@northwestern.edu

Tel: (312) 503-4494

Fax: (312) 503-0411

Declaration of interests: NM is currently an employee and owns stock in Vertex Pharmaceuticals. BDS holds United States Patent No. US 10,905,706 B2, Compositions and Methods to Accelerate Resolution of Acute Lung Inflammation, and serves on the Scientific Advisory Board of Zoe Biosciences. The other authors have no competing interests to declare.

Supplemental (Complete) Methods

Sex as a biological variable. We present data in **Supplemental Figure 1** demonstrating that Treg cell-specific AMPK deficient mice breed in approximately mendelian sex ratios. Neither sex developed spontaneous developmental pathology. In preliminary experiments using the B16 melanoma model in wild-type mice, we found that female mice grow smaller tumors than male mice. As the phenotype due to Treg cell-specific AMPK deficiency was elicited at high tumor volumes, male mice were used for these experiments. In preliminary experiments using the influenza model in wild-type mice, we found that female mice experience higher mortality than males. As mice with Treg cell-specific AMPK deficiency experienced higher mortality than controls, male mice were used for these experiments. Hence, sex was not considered as a biological variable in all experiments.

Mice. *Prkaa1*$^{fl/fl}$ (cat. no. 014141), *Prkaa2*$^{fl/fl}$ (cat. no. 014142), and *Foxp3*$^{YFP\text{-}Cre}$ (cat. no. 016959) mice from the C57BL/6J genetic background were purchased from The Jackson Laboratory and bred to generate Treg cell-specific AMPKα1 and AMPKα2 double KO mice (*Prkaa1*$^{fl/fl}$*xPrkaa2*$^{fl/fl}$*xFoxp3*$^{YFP\text{-}Cre}$) and Treg cell-specific AMPKα1 and AMPKα2 single KO mice (*Prkaa1*$^{fl/fl}$*xFoxp3*$^{YFP\text{-}Cre}$ and *Prkaa2*$^{fl/fl}$*xFoxp3*$^{YFP\text{-}Cre}$, respectively). All animals were genotyped using services provided by Transnetyx Inc., with primers provided by The Jackson Laboratory and shown in **Supplemental Table 1**; RT-PCR primers are also shown here. Animals received water *ad libitum*, were housed at a temperature range of 20 °C–23 °C under 14-hour light/10-hour dark cycles and received standard rodent chow.

Flow cytometry and cell sorting. Single-cell suspensions of organ tissues, blood, tumors, or cultured cells were prepared and stained for flow cytometry analysis and sorting as previously described (1, 2) using the reagents shown in **Supplemental Table 2**. Cell counts of single-cell suspensions were

obtained using a Cellometer with AO/PI staining (Nexcelom Bioscience cat. # SD014-0106) before preparation for flow cytometry. Data acquisition for analysis was performed using a BD LSRFortessa or Symphony A5 instrument with FACSDiva software (BD). Cell sorting was performed using the 4-way purity setting on BD FACSAria SORP instruments with FACSDiva software or with a microfluidics MACSQuant Tyto sorter (Miltenyi). Analysis was performed with FlowJo, version 10.9.0 software. Dead cells were excluded using a viability dye for analysis and sorting (3).

Imaging flow cytometry measurement of mitophagy. Splenic single cell suspensions were stained with surface markers and MitoView Green (20 nM) and treated with 10 μM CCCP for 30, 60, 120, and 180 minutes to induce mitophagy. Samples were then fixed as above. Fixed single cell suspensions were then stained with anti-LAMP1 and anti-FOXP3 antibodies at 4 °C for 30 minutes. Imaging flow cytometry was performed using a BD FACS Discover S8 cell sorter and analyzer and the subcellular co-localization of LAMP1 and MitoView Green signal was assessed using BD CellView Image Technology in the BD FACSChorus software.

iTreg cell induction and culture. CD4+CD25- T cells were enriched from splenocyte single cell suspensions using the negative fractions from the Miltenyi mouse CD4+CD25+ Regulatory T Cell Isolation Kit (cat. no. 130-091-041) according to manufacturer instructions or sorted via flow cytometry. CD4+CD25- T cells were seeded in a 24-well plate at a concentration of $3x10^5$ cells/mL and cultured for 3–5 days in complete RPMI medium (10% FBS) containing recombinant IL-2 (50 IU/mL) and TGF-β (10 ng/mL) (1). *Foxp3*-YFP+ cells were sorted using flow cytometry for downstream use.

B16 melanoma tumor model. B16-F10 cells (ATCC CRL-6475) were cultured in RPMI (10% FBS, 5% L-glutamine, 5% sodium pyruvate, 5% penicillin/streptomycin, 2.5% HEPES buffer), harvested at 60–70% confluence, and counted using a Cellometer with AO/PI staining (Nexcelom Bioscience cat. #

SD014-0106) as previously described (1). 250,000 B16-F10 cells were resuspended in 0.1 mL of PBS and 40% Matrigel (Corning cat. # 356237) and injected subcutaneously in the hair-trimmed flanks of 12–15-week-old mice. Tumor length and width were measured every other day starting day 7 post-engraftment up to day 21 post-engraftment. A subset of tumors was resected post-mortem for flow cytometry analysis and sorting as follows: subcutaneous tumors were harvested from euthanized mice and minced with surgical scissors in 3 mL of HBSS containing 2 mg of collagenase (Sigma Aldrich cat. no. 11088866001) and 0.25 mg of DNAse I (Sigma Aldrich cat. no. 10104159001) per mL. Tumor homogenates were incubated at 37 °C for 45 minutes, transferred to gentleMACS™ C Tubes (Miltenyi Biotec cat. # 130-093-237) and processed with the mouse implanted tumor protocol (m_impTumor_01). Tumor homogenates were then subjected to debris removal (Miltenyi cat. # 130-109-398) and dead cell removal (Miltenyi cat. # 130-090-101) according to the kit manufacturer's instructions. The debris and dead cell-depleted tumor homogenates were then stained for flow cytometry and cell sorting.

Influenza A virus administration. Mice were anesthetized with isoflurane and intubated using a 20-gauge angiocatheter cut to a length that placed the tip of the catheter above the carina. Mice were instilled with mouse-adapted influenza A/WSN/33 [H1N1] virus (12.5 plaque-forming units in 50 µL of sterile PBS) as previously described (2).

Measurement of physiologic readouts of influenza pneumonia progression and resolution. Arterial blood oxygen saturation (SpO_2) was measured in control and influenza virus-infected mice using a MouseOx Plus pulse oximeter (Starr Life Sciences). Beginning on the fifth day post-inoculation and continuing every other, SpO_2 was measured with oximeter collar clips secured to the hairless neck of conscious, immobilized animals. Mouse weights were recorded the day of influenza virus inoculation and every other day post-inoculation starting day 5. Mouse weights were normalized to those recorded on the day of inoculation.

Lung tissue harvesting and processing. Influenza virus-infected mice were euthanized and slowly infused with HBSS through the right atrium of the heart, clearing the pulmonary circulation of blood. The lungs were harvested and grossly homogenized with scissors in HBSS containing 2 mg of collagenase D (Sigma Aldrich cat. no. 11088866001) and 0.25 mg of DNAse I (Sigma Aldrich cat. no. 10104159001) per mL, incubated for 45 minutes at room temperature and then further homogenized using the mouse lung protocol of the Miltenyi OctoMACS tissue dissociator (m_lung_02). These procedures have been previously reported (2).

Immunoblotting. Cultured cells were lysed for one hour at 4 °C in lysis buffer (Cell Signaling cat. no. 9803) supplemented with phosphatase (Cell Signaling cat. no. 5870S) and protease inhibitors (Roche, cat. no. 65726900) after which their concentration was measured with a BCA assay according to manufacturer instructions (Pierce cat. no. 23225). Cell lysates were subjected to gel electrophoresis and transferred to membranes that were incubated with an antibody against AMPKα1 (Abcam cat. no. ab32047), DNMT1 (Cell Signaling cat. no. 5032) and β-actin (Abcam cat. no. ab8227) overnight at 4 °C with constant agitation.

Wes protein immunoassay. Flow cytometry-sorted cells were lysed, and the resulting lysate protein concentrations were measured as described above. For protein measurements using the Simple Wes immunoassay system, 0.5 μg of protein in 3 μL were loaded per well and processed according to manufacturing instructions. The following concentrations were used for primary antibodies: 1:50 anti-DNMT1 (Invitrogen cat. no. MA5-16169), 1:50 anti-AMPKα (Cell Signaling cat. no. 2532S), and 1:50 anti-β-actin (Abcam cat. no. ab8227).

Co-immunoprecipitation assay. 10^6 cells were lysed in cell lysis buffer for one hour at 4 °C as described above. Lysates were incubated with an antibody against AMPKα1 (Abcam cat. no. ab32047) or isotype control (Cell Signaling cat. no. 7074) overnight at 4 °C with constant agitation. The immune complex was precipitated with Dyna Protein G beads (Life Technologies cat. no. 10003D), washed and resuspended in SDS/PAGE loading buffer, and heated to 95 °C for 5 minutes. Processed samples were then blotted with antibodies against DNMT1 (Cell Signaling cat. no. 5032), AMPKα1 (Abcam cat. no. ab32047), and β-actin (Abcam cat. no. ab8227). Jurkat cells were obtained from ATCC. MT-2 cells were a gift from Jason R. Mock, MD, PhD (University of North Carolina, Chapel Hill, NC).

Immunofluorescence for microscopy. 10^6 cells were fixed with ice-cold 100% methanol for 5 minutes. Subsequently, samples were processed with Immunofluorescence Application Solutions Kit (Cell Signaling cat. no. 12727) following the manufacturer's protocol. Cells were stained overnight at 4 °C with anti-DNMT1 (Abcam cat. no. ab21799 1), anti-AMPKα1 (Abcam cat. no. ab32047), Alexa fluor 488-conjugated isotype control (Abcam cat. no. ab199091), or unconjugated isotype control (Abcam cat. no. ab172730). The following day, cells that were stained with anti-AMPKα1 antibody and unconjugated isotype control antibody were incubated in the dark at room temperature for 2 hours with anti-rabbit Alexa Fluor 488 secondary antibody (Abcam cat. no. ab150113). Following antibody incubation, cells were mounted on a slide with VECTASHIELD Vibrance mounting medium containing DAPI (Vector Labs cat. no. H-1800). Fluorescent images were acquired at room temperature using a confocal microscope (Nikon) with 40× magnification at the Northwestern Center for Advanced Microscopy.

Nuclear-cytoplasmic fractionation assay. 5×10^6 MT-2 cells were treated and subsequently underwent lysis using NE-PER™ Nuclear and Cytoplasmic Extraction kit (ThermoFisher cat. no. 78833)

according to manufacturer's protocol. Nuclear and cytoplasmic fractions were collected and further analyzed for the expression of proteins of interest with immunoblotting as described above.

Metabolic flux (Seahorse) assay. 2.5x10^5 flow cytometry-sorted Treg cells were seeded on a 96-well Seahorse cell culture plate and analyzed on a Seahorse XF24 Analyzer (4). The following drugs and corresponding doses were loaded onto ports A, B, C, and D in the same order: oligomycin (2.5 μM, Sigma-Aldrich cat no. 75351), CCCP (10 μM, Sigma-Aldrich cat no. C2759), antimycin A/piercidin (2 μM each, Sigma-Aldrich cat no. A8674 and 15379, respectively), and 2-deoxyglucose (25 mM, Sigma-Aldrich cat no. D8375).

RNA-sequencing, modified reduced representation bisulfite sequencing (mRRBS) and analysis. 10^3–10^5 flow cytometry sorted cells were lysed immediately after sorting with QIAGEN RLT Plus containing 1% β-mercaptoethanol and subjected to RNA and DNA isolation using the QIAGEN AllPrep Micro Kit as previously described (1, 2, 5). RNA-seq library preparation was performed using the SMARTer Stranded Total RNA-Seq Kit, version 2 (Takara cat. no. 634411) and mRRBS library preparation was performed using custom procedures previously described by our group (1, 2, 5). After sequencing, raw binary base call (BCL) files were converted to FASTQ files using bcl2fastq (Illumina). All FASTQ files were processed using the nf-core/RNA-seq pipeline (6) version 3.9 implemented in Nextflow (7, 8) 23.04.2 with Northwestern University Quest HPC (Genomic Nodes) configuration (nextflow run nf-core/rnaseq -profile nu_genomics --genome GRCm38). In short, lane-level reads were trimmed using trimGalore! 0.6.5, aligned to the GRCm38 reference genome using STAR 2.7.11, and quantified using Salmon. All samples showed satisfactory alignment rate (>60%). After quantification, differential expression analysis was performed in R version 4.2.0 using DEseq2 v1.38.3 (9). Sample genotype was used as an explanatory factor. Within-group homogeneity was first confirmed by principal component analysis (PCA) and no outliers were found. *K*-means clustering of differentially expressed

genes ($q < 0.05$) was performed using a previously published custom R function (10). In brief, *k* was first determined using the elbow plot and the kmeans function in R stats 3.6.2 (Hartigan–Wong method with 25 random sets and a maximum of 1,000 iterations) was used for *k*-means clustering. Samples were clustered using Ward's method and a heatmap was generated using pheatmap version 1.0.12. Subsequent GO term enrichment was performed using topGO version 2.50.0 with Fisher's exact test. org.Mm.eg.db version 3.16.0 and GO.db version 3.16.0 were included in GO terms enrichment analysis as references. Gene Set Enrichment Analysis was performed using the GSEA version 4.0.3 GSEAPreranked tool with genes ordered by $\log_2$(fold-change) in average expression (11). mRRBS analysis was performed using previously published procedures (12).

Collection of lung Treg cells for metabolomics. Lung single-cell suspensions were subjected to CD4+ cell positive enrichment according to kit manufacturer's instructions (Miltenyi Biotec, cat. no. 130-097-048) before fluorochrome staining. Fluorochrome stained cells were then resuspended in sorting buffer (Miltenyi Biotec cat. no. 130-107-207) to achieve a concentration of $2x10^6$ cells / mL of buffer. Using a MACSQuant Tyto, $5–10x10^5$ lung Treg cells were sorted from each pair of lungs. Sorted cells were centrifuged at 500 rcf for 6 minutes, 4 °C. After the sorting media was removed via pipetting, the pelleted cells were resuspended in 15 µl of 80% acetonitrile and vortexed for 30 seconds. Following centrifugation for 30 minutes at 20,000 rcf, 4 °C, the supernatant was collected for high-performance liquid chromatography and high-resolution mass spectrometry and tandem mass spectrometry (LC-MS) analysis.

Collection of interstitial fluid and plasma for metabolomics. Blood was collected from both influenza-infected and B16-melanoma tumor-engrafted mice via insertion of glass capillaries through the retro-orbital sinus. This blood was centrifuged at 800 rcf for 10 minutes at 4 °C in EDTA tubes. The plasma phase was pipetted, frozen with liquid nitrogen, and stored at -80 °C. Subcutaneous B16-F10

melanoma tumors were harvested on day 12 or 15 post-engraftment. Lungs of mice that received 12.5 plaque-forming units of influenza A virus intra-tracheally were harvested on day 10 post-inoculation. The tumors were rinsed in phosphate buffered saline and dried with task wipers. The lungs were perfused via injection of 10 mL of Hanks Balanced Salt Solution (HBSS) through the heart's right ventricle before harvesting all soft organs of the mediastinum. The mediastinal structures surrounding the lungs were removed via gross dissection. The intact tumors and lungs were then centrifuged at 100 rcf for 10 minutes, 4 °C in centrifuge tubes containing a 0.22 µm filter (Costar cat. no. 8160). The extracted interstitial fluid was then diluted 1 to 5 in 80% acetonitrile and vortexed for 30 seconds. The diluted interstitial fluid was centrifuged for 30 min at 20,000 rcf at 4 °C and the supernatant was collected for LC-MS analysis.

High-performance liquid chromatography and high-resolution mass spectrometry and tandem mass spectrometry (LC-MS) for metabolomics. The system consisted of a Thermo Q-Exactive in line with an electrospray source and an Ultimate3000 (Thermo) series HPLC consisting of a binary pump, degasser, and auto-sampler outfitted with a Xbridge Amide column (Waters; dimensions of 3.0 mm × 100 mm and a 3.5 µm particle size). The mobile phase A contained 95% (vol/vol) water, 5% (vol/vol) acetonitrile, 10 mM ammonium hydroxide, 10 mM ammonium acetate, pH = 9.0; B was 100% acetonitrile. The gradient was as follows: 0 minutes, 15% A; 2.5 minutes, 30% A; 7 minutes, 43% A; 16 minutes, 62% A; 16.1-18 minutes, 75% A; 18-25 minutes, 15% A with a flow rate of 150 µL/minute. The capillary of the ESI source was set to 275 °C, with sheath gas at 35 arbitrary units, auxiliary gas at 5 arbitrary units, and the spray voltage at 4.0 kV. In positive/negative polarity switching mode, an *m*/*z* scan range from 60 to 900 was chosen and MS1 data was collected at a resolution of 70,000. The automatic gain control (AGC) target was set at 1×10^6 and the maximum injection time was 200 ms. The top 5 precursor ions were subsequently fragmented, in a data-dependent manner, using the higher energy collisional dissociation (HCD) cell set to 30% normalized collision energy in MS2 at a resolution

power of 17,500. Besides matching m/z, metabolites are identified by matching either retention time with analytical standards and/or MS2 fragmentation pattern. Data acquisition and analysis were carried out by Xcalibur 4.1 software and Tracefinder 4.1 software, respectively (both from Thermo Fisher Scientific).

LC-MS data analysis. Raw peak intensity data of the metabolites detected by LC-MS were uploaded to Metaboanalyst 5.0's statistical analysis [one factor] module. For comparisons with more than two groups, one-way ANOVA with $q < 0.05$ was employed to identify significant differentially enriched metabolites. Comparisons with only two groups were analyzed with multiple parametric t-tests and fold-change analysis using Metabonalyst 5.0's standard settings ($p < 0.1$). Fold change threshold was set to 1.5 in resulting volcano plots to increase the power of the downstream overrepresentation analysis. The names of significant differentially enriched metabolites were used as inputs for Metaboanalyst 5.0's enrichment analysis module. The KEGG human metabolic pathway metabolite set library was selected for enrichment analysis.

Statistical analysis. *p*-values and FDR *q*-values resulting from two-tailed tests were calculated using statistical tests stated in the figure legends using GraphPad Prism v10.1.0. Differences between groups with *p*- or *q*-values < 0.05 were considered statistically significant; see LC-MS data analysis for the statistical approach to metabolomic profiling data and RNA-sequencing, modified reduced representation bisulfite sequencing (mRRBS) and analysis for the statistical approach to transcriptomic and epigenomic profiling data. Using the ROUT method, the following number of outliers were excluded from the following figures: one from Figure 2B (Q = 0.5%), two from Figure 2C (Q = 0.5%), four from Supplemental Figure 2L (Q = 1.0%), and three from Supplemental Figure 4B (Q = 1.0%). Central tendency and error are displayed as mean ± standard deviation (SD) except as noted. Box plots show median and quartiles. Numbers of biological replicates are stated in the figures or accompanying

legends. Computational analysis was performed using Genomics Nodes and Analytics Nodes on Quest, Northwestern University's High-Performance Computing Cluster.

Study approval. All mouse procedures were approved by the Northwestern University IACUC under protocols IS00012519 and IS00017837.

Data availability. The raw and processed next-generation sequencing data sets were deposited in the NCBI's Gene Expression Omnibus database (GEO GSE249019). Raw peak intensity data of annotated metabolites detected by LC-MS are available in the data supplement. All raw data is included in the Supporting Data Values file.

Supplemental References

1. Helmin KA, et al. Maintenance DNA methylation is essential for regulatory T cell development and stability of suppressive function. *J Clin Invest.* 2020;130(12):6571-6587.
2. Morales-Nebreda L, et al. Aging imparts cell-autonomous dysfunction to regulatory T cells during recovery from influenza pneumonia. *JCI Insight.* 2021;6(6).
3. Tighe RM, et al. Improving the quality and reproducibility of flow cytometry in the lung. An official American Thoracic Society workshop report. *Am J Respir Cell Mol Biol.* 2019;61(2):150-161.
4. Weinberg SE, et al. Mitochondrial complex III is essential for suppressive function of regulatory T cells. *Nature.* 2019;565(7740):495-499.
5. McGrath-Morrow SA, et al. DNA methylation regulates the neonatal CD4(+) T-cell response to pneumonia in mice. *J Biol Chem.* 2018;293(30):11772-11783.
6. Harshil Patel PE, Alexander Peltzer, Olga Botvinnik, Gregor Sturm, Denis Moreno, Pranathi Vemuri, Maxime U Garcia, silviamorins, Lorena Pantano, Mahesh Binzer-Panchal, nf-core bot, Robert Syme, Matthias Zepper, Gavin Kelly, Friederike Hanssen, James A. Fellows Yates, Chris Cheshire, rfenouil, … Paolo Di Tommaso. Nf-core/rnaseq: Nf-core/rnaseq v3.12.0 - osmium octopus (3.12.0). Zenodo. 2023. https://doi.org/10.5281/zenodo.7998767.
7. Ewels PA, et al. The nf-core framework for community-curated bioinformatics pipelines. *Nat Biotechnol.* 2020;38(3):276-278.
8. Di Tommaso P, Chatzou M, Floden EW, Barja PP, Palumbo E, Notredame C. Nextflow enables reproducible computational workflows. *Nat Biotechnol.* 2017;35(4):316-319.
9. Love MI, Huber W, Anders S. Moderated estimation of fold change and dispersion for RNA-seq data with DESeq2. *Genome Biol.* 2014;15(12):550.
10. Grant RA, et al. Circuits between infected macrophages and T cells in SARS-CoV-2 pneumonia. *Nature.* 2021;590(7847):635-641.

11. Subramanian A, et al. Gene set enrichment analysis: A knowledge-based approach for interpreting genome-wide expression profiles. *Proc Natl Acad Sci U S A.* 2005;102(43):15545-15550.

12. Singer BD. A practical guide to the measurement and analysis of DNA methylation. *Am J Respir Cell Mol Biol.* 2019;61(4):417-428.

	Allele	Primer direction	Primer sequence
Genotyping primers	*Prkaa1*^{fl}	Forward	GCAGCCCAATTCCGATCATATTCA
		Reverse	GCCTGCTTTGCACACTTATGG
	Prkaa1^{wt}	Forward	CGACTAGCTTCATGTCCTGTTTTCT
		Reverse	CCTGCTTGGCACACTTATGGTAA
	Prkaa2^{fl}	Forward	GCAGCCCAATTCCGATCATATTCA
		Reverse	TGGAACATCTTTTAGACAGAATAATCTTTTAGACAG
	Prkaa2^{wt}	Forward	CGAAAAACTCAAAAAAATTATTATTCTGTAATAGATAGTGATGTTAAA
		Reverse	CCTTCATTAGAATACTATGGAACATCTTTTAGACA

RT-PCR primers	*Prkaa1*^{wt}	Forward	GCTGTGGCTCACCCAATTAT
		Reverse	TGTTGTACAGGCAGCTGAGG
	Prkaa2^{wt}	Forward	CGGCTCTTTCAGCAGATTCTGT
		Reverse	ATCGGCTATCTTGGCATTCATG

Supplemental Table 1. Primer sequences used to genotype and profile the expression of the *Prkaa1* and *Prkaa2* loci.

Antigen/Reagent	Conjugate	Clone	Manufacturer	Catalog no.
CD45	BUV563	30-F11	BD Horizon	612924
CD4	BUV395	GK1.5	BD Horizon	563790
CD8	BUV805	53-6.7	BD Horizon	612898
CD8	BV711	53-6.7	Biolegend	100747
CD25	BV605	PC61	Biolegend	102036
CD25	APC	PC61.5	Invitrogen	2154040
CD62L	APC-e780	MEL-14	Invitrogen	2011195
CD44	BUV737	IM7	BD Horizon	564392
PD-1	PE/Dazzle594	RMP1-30	Biolegend	109116
PD-1	PerCP-eFluor710	J43	Invitrogen	46-9985-82
CTLA-4	BV421	UC10-4B9	Biolegend	106312
TIGIT	BV711	1G9	BD Biosciences	744214
ICOS	BV786	C398.4A	BD Biosciences	567922
Ki-67	Alexa Fluor 488	11F6	Biolegend	151204
FOXP3	PE-Cy7	FJK-16s	BD Biosciences	25-5773-82
LC3B	PE	D11	Biolegend	8899S
LAMP-1	PE-Cy7	1D4B	eBioscience	25-1071-82
Fixable Viability Dye eFluor506	N/A	N/A	eBioscience	65-0866-14
Cell Trace Violet	N/A	N/A	Invitrogen	C34571
Absolute counting beads	N/A	N/A	Invitrogen	C36950
Mitotracker Deep Red	N/A	N/A	Invitrogen	M22426
MitoView Green	N/A	N/A	Biotium	70054

Supplemental Table 2. Flow cytometry fluorochromes and reagents. Fixable viability dye eFluor 506, cell trace violet, and absolute counting beads were used according to manufacturer instructions. MitoTracker Deep Red staining: After surface marker fluorochrome stain, single-cell suspensions were stained with 50 nM MitoTracker Deep Red in complete RPMI for 30 minutes at 37 °C.

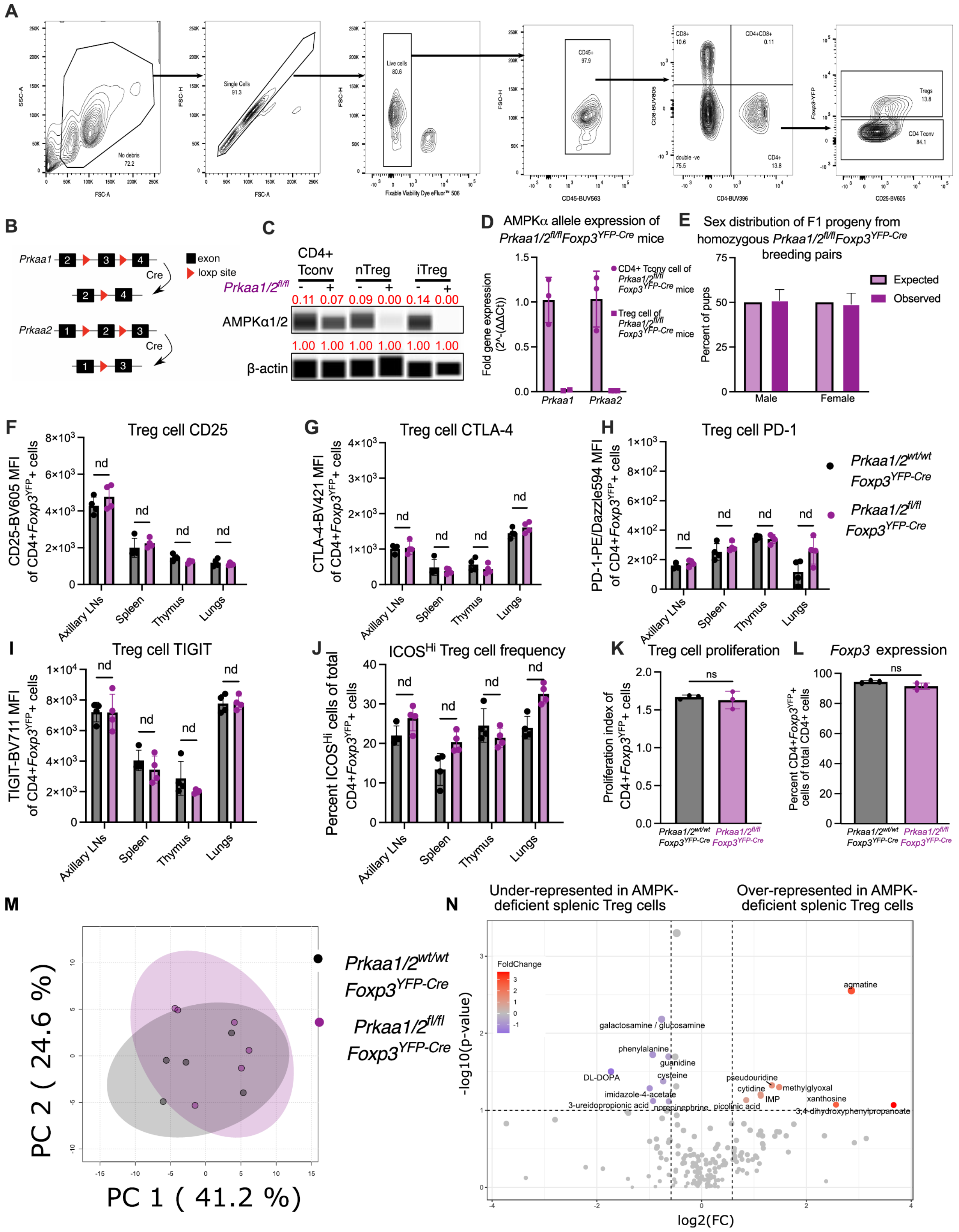

Supplemental Figure 1. Validation of AMPKα1/α2 conditional knockout and Treg cell and mouse phenotyping of *Prkaa1/2$^{fl/fl}$Foxp3$^{YFP-Cre}$* and control mice. (**A**) Flow cytometry gating strategy for

phenotyping of T cell populations, including CD4+*Foxp3*^{YFP}+ (Treg) cells. (**B**) Schematic of the *Foxp3*$^{YFP\text{-}Cre}$-mediated excision of loxP-flanked exons of *Prkaa1* and *Prkaa2* in Treg cells. (**C**) Validation of Treg cell-specific knockout of AMPKα1/α2 using the Simple Wes immunoassay system (*n*=3 replicates per group and cell type pooled and run as single well); signal intensities normalized to the corresponding lane's β-actin signal in red. (**D**) Relative expression of *Prkaa1* and *Prkaa2* by CD4+*Foxp3*^{YFP}- T (CD4+ Tconv) cells and Treg cells of *Prkaa1/2*$^{fl/fl}$*Foxp3*$^{YFP\text{-}Cre}$ mice (*n*=3 Tconv cells, *n*=2 Treg cells). (**E**) Observed and expected frequencies of F1 pups from crosses of male *Prkaa1/2*$^{fl/fl}$*Foxp3*$^{YFP\text{-}Cre/Y}$ mice with female *Prkaa1/2*$^{fl/fl}$*Foxp3*$^{YFP\text{-}Cre/YFP\text{-}Cre}$ mice. Chi-square test for goodness of fit p = 0.75. Chi-square = 0.1 with 1 degree of freedom (n=127 males and 122 females). (**F**-**I**) CD25-BV605 (F), CTLA-4-BV421 (G), PD-1-PE/Dazzle594 (H), and TIGIT-BV711 (I) mean fluorescence intensity (MFI) of Treg cells from *Prkaa1/2*$^{wt/wt}$*Foxp3*$^{YFP\text{-}Cre}$ (control) and *Prkaa1/2*$^{fl/fl}$*Foxp3*$^{YFP\text{-}Cre}$ mouse axillary lymph nodes (LN), spleen, thymus, and lung (*n*=4 control, *n*=4 *Prkaa1/2*$^{fl/fl}$*Foxp3*$^{YFP\text{-}Cre}$). (**J**) Frequency of ICOS^{Hi} CD4+*Foxp3*^{YFP}+ cells of total CD4+*Foxp3*^{YFP}+ cells from control and *Prkaa1/2*$^{fl/fl}$*Foxp3*$^{YFP\text{-}Cre}$ mouse LN, spleen, thymus, and lung (*n*=4 control, *n*=4 *Prkaa1/2*$^{fl/fl}$*Foxp3*$^{YFP\text{-}Cre}$). (**K**) Proliferation index of Cell Trace Violet+ CD4+*Foxp3*^{YFP}+ cells according to FlowJo 10.9.0's proliferation modeling tool (*n*=3 control, *n*=3 *Prkaa1/2*$^{fl/fl}$*Foxp3*$^{YFP\text{-}Cre}$). (**L**) Frequency of CD4+*Foxp3*^{YFP}+ cells of total CD4+ cells after in vitro treatment of CD4+Foxp3- splenocytes with Treg cell-polarizing conditions for 5 days (*n*=3 control, *n*=3 *Prkaa1/2*$^{fl/fl}$*Foxp3*$^{YFP\text{-}Cre}$). (**M**-**N**) Principal component (PC) analysis (M) and volcano plot (N) of LC-MS data generated with metabolites extracted from splenic Treg cells of control (*n*=5) and *Prkaa1/2*$^{fl/fl}$*Foxp3*$^{YFP\text{-}Cre}$ (*n*=6) mice. ns not significant, nd no discovery according to Mann-Whitney *U* test (K-L) with two-stage linear step-up procedure of Benjamini, Krieger, and Yekutieli with Q = 5% (F-J).

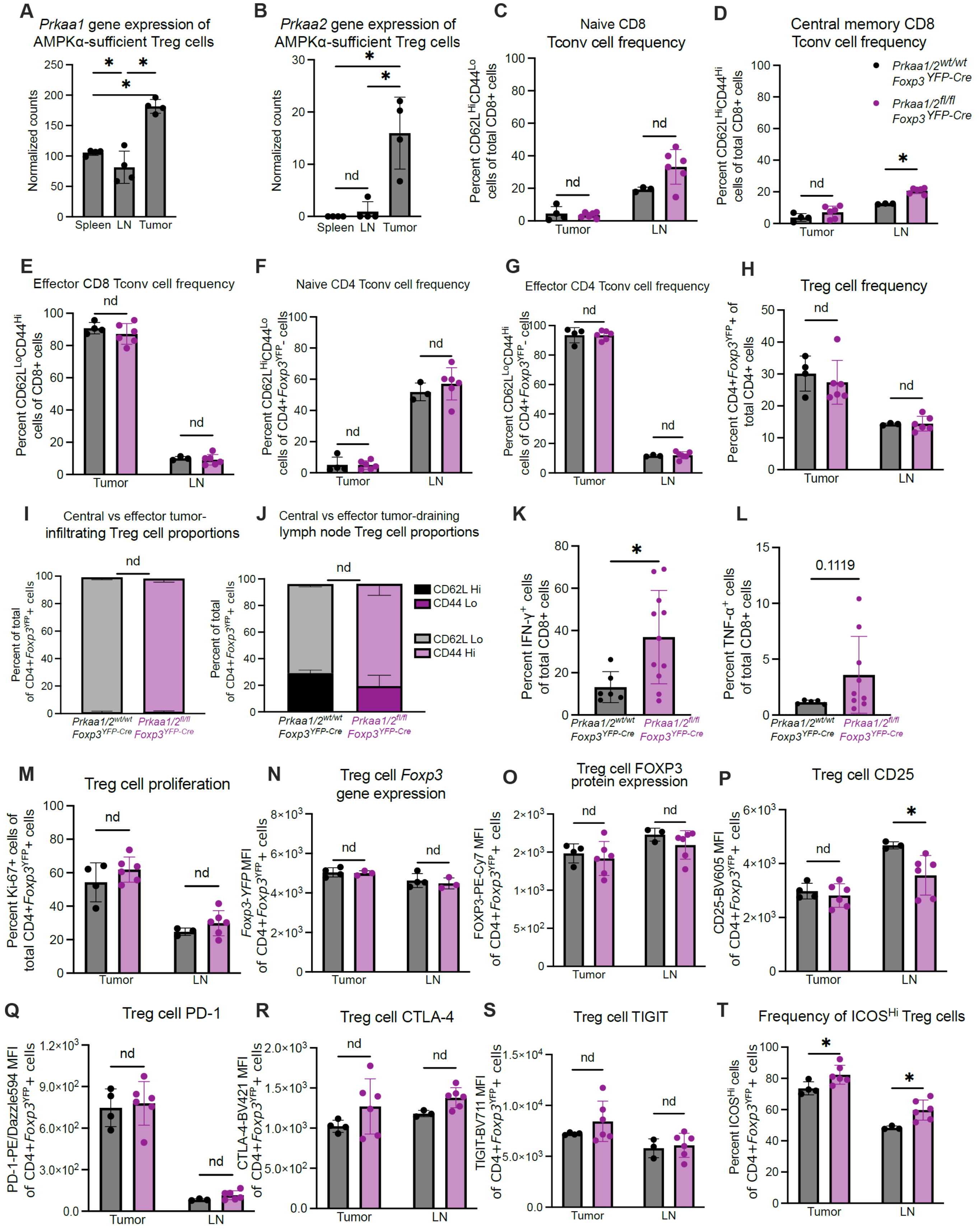

Supplemental Figure 2. Immune phenotyping of tumor-infiltrating T cell subsets of *Prkaa1/2*$^{fl/fl}$*Foxp3*$^{YFP\text{-}Cre}$ and control mice. (**A-B**) Normalized gene expression counts of *Prkaa1* (A)

and *Prkaa2* (B) by CD4+*Foxp3*^{YFP}+ cells sorted from tumors, spleens, and lymph nodes of tumor-bearing *Prkaa1/2*$^{wt/wt}$*Foxp3*$^{YFP\text{-}Cre}$ (control, *n*=4) mice. (**C**-**E**) Frequency of naive (CD62L^{Hi}CD44^{Lo}; C), central memory (CD62L^{Hi}CD44^{Hi}; D), and effector (CD62L^{Lo}CD44^{Hi}; E) CD8+ conventional T (Tconv) cells out of total CD8+ cells in tumors (*n*=4 control, *n*=6 *Prkaa1/2*$^{fl/fl}$*Foxp3*$^{YFP\text{-}Cre}$) and tumor-draining lymph nodes (LN; *n*=3 control, *n*=6 *Prkaa1/2*$^{fl/fl}$*Foxp3*$^{YFP\text{-}Cre}$ mice). (**F**-**G**) Frequency of naive (F) and effector (G) Effector Tconv cells out of total CD4+*Foxp3*^{YFP}- cells in tumors (*n*=4 control, *n*=6 *Prkaa1/2*$^{fl/fl}$*Foxp3*$^{YFP\text{-}Cre}$ mice) and LN (*n*=3 control, *n*=6 *Prkaa1/2*$^{fl/fl}$*Foxp3*$^{YFP\text{-}Cre}$ mice). (**H**) Percent of CD4+*Foxp3*^{YFP}+ cells out of total CD4+ cells in tumors (*n*=4 control, *n*=6 *Prkaa1/2*$^{fl/fl}$*Foxp3*$^{YFP\text{-}Cre}$ mice) and LN (*n*=3 control, *n*=6 *Prkaa1/2*$^{fl/fl}$*Foxp3*$^{YFP\text{-}Cre}$ mice). (**I**-**J**) Frequency of central (CD62L^{Hi}CD44^{Lo}) and effector (CD62L^{Lo}CD44^{Hi}) CD4+*Foxp3*^{YFP}+ cells out of total CD4+*Foxp3*^{YFP}+ cells in tumors (I; *n*=4 control, *n*=6 *Prkaa1/2*$^{fl/fl}$*Foxp3*$^{YFP\text{-}Cre}$ mice) and LN (J; *n*=3 control, *n*=6 *Prkaa1/2*$^{fl/fl}$*Foxp3*$^{YFP\text{-}Cre}$ mice). (**K**-**L**) Frequency of IFN-γ+ (*n*=6 control, *n*=11 *Prkaa1/2*$^{fl/fl}$*Foxp3*$^{YFP\text{-}Cre}$ mice) (K) and TNF-α+ (*n*=5 control, *n*=9 *Prkaa1/2*$^{fl/fl}$*Foxp3*$^{YFP\text{-}Cre}$ mice) (L) cells of total CD8+ T cells (**M**) Frequency of Ki-67+CD4+*Foxp3*^{YFP}+ cells out of total CD4+*Foxp3*^{YFP}+ cells in tumors (*n*=4 control, *n*=6 *Prkaa1/2*$^{fl/fl}$*Foxp3*$^{YFP\text{-}Cre}$ mice) and LN (*n*=3 control, *n*=6 *Prkaa1/2*$^{fl/fl}$*Foxp3*$^{YFP\text{-}Cre}$ mice). (**N**) *Foxp3*^{YFP} mean fluorescence intensity (MFI) of CD4+*Foxp3*^{YFP}+ cells in tumors (*n*=4 control, *n*=6 *Prkaa1/2*$^{fl/fl}$*Foxp3*$^{YFP\text{-}Cre}$ mice) and LN (*n*=3 control, *n*=6 *Prkaa1/2*$^{fl/fl}$*Foxp3*$^{YFP\text{-}Cre}$ mice). (**O**-**S**) FOXP3-PE-Cy7 (O), CD25-BV605 (P), PD-1-PE/Dazzle594 (Q), CTLA-4-BV421 (R) and TIGIT-BV711 (S) MFI of CD4+*Foxp3*^{YFP}+ cells from tumors (*n*=4 control, *n*=6 *Prkaa1/2*$^{fl/fl}$*Foxp3*$^{YFP\text{-}Cre}$ mice) and LN (*n*=3 control, *n*=6 *Prkaa1/2*$^{fl/fl}$*Foxp3*$^{YFP\text{-}Cre}$ mice). (**T**) Frequency of ICOS^{Hi} CD4+*Foxp3*^{YFP}+ cells out of total CD4+*Foxp3*^{YFP}+ cells in tumors (*n*=4 control, *n*=6 *Prkaa1/2*$^{fl/fl}$*Foxp3*$^{YFP\text{-}Cre}$ mice) and LN (*n*=3 control, *n*=6 *Prkaa1/2*$^{fl/fl}$*Foxp3*$^{YFP\text{-}Cre}$ mice). * $q < 0.05$, nd no discovery according to Mann Whitney U test (K-L) with two-stage linear step-up procedure of Benjamini, Krieger, and Yekutieli with Q = 5% (A-J,M-T). Four outliers identified and excluded from (L) using the ROUT method (Q=1%).

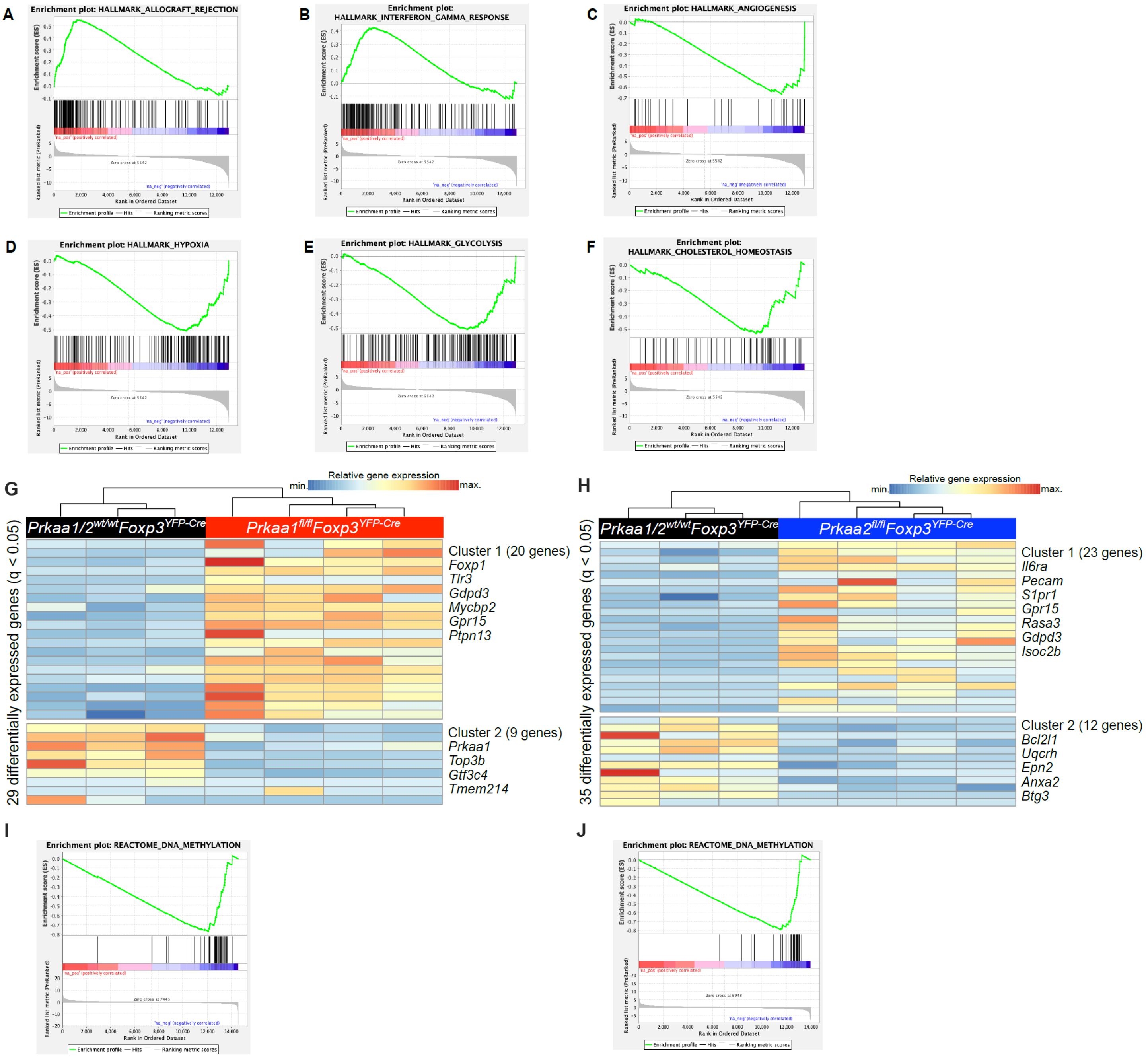

Supplemental Figure 3. Transcriptional phenotyping of tumor-infiltrating AMPKα1/α2-deficient, AMPKα1-deficient, AMPKα2-deficient, and control Treg cells at day 12 post-engraftment. (**A-F**) Enrichment plots ($p < 0.05$, FDR $q < 0.25$) of the HALLMARK_ALLOGRAFT_REJECTION (A), HALLMARK_INTERFERON_GAMMA_RESPONSE (B), HALLMARK_ANGIOGENESIS (C), HALLMARK_HYPOXIA (D), HALLMARK_GLYCOLYSIS (E), and HALLMARK_CHOLESTEROL HOMEOSTASIS (F) gene sets. Enrichment plots were generated through GSEA preranked testing of the expressed genes of tumor-infiltrating *Prkaa1/2*$^{fl/fl}$*Foxp3*$^{YFP\text{-}Cre}$ and control Treg cells identified by RNA-sequencing at day 15 post-engraftment. (**G-H**) *K*-means clustering of significant differentially expressed genes (FDR $q < 0.05$) identified between Treg cells sorted from B16 melanoma tumors of *Prkaa1*$^{fl/fl}$*Foxp3*$^{YFP\text{-}Cre}$ (n=4) mice versus controls (G) and *Prkaa2*$^{fl/fl}$*Foxp3*$^{YFP\text{-}Cre}$ (n=4) mice versus controls (H) at day 12 post-engraftment, with k=2 and scaled as Z-scores across rows. (**I-J**) Enrichment plots ($p < 0.05$, FDR $q < 0.25$) of the REACTOME_DNA_METHYLATION gene set generated through

GSEA preranked testing of the expressed genes of tumor-infiltrating *Prkaa1*$^{fl/fl}$*Foxp3*$^{YFP\text{-}Cre}$ and control Treg cells (G) as well as *Prkaa2*$^{fl/fl}$*Foxp3*$^{YFP\text{-}Cre}$ and control Treg cells (H) identified on day 12 post-engraftment RNA-sequencing.

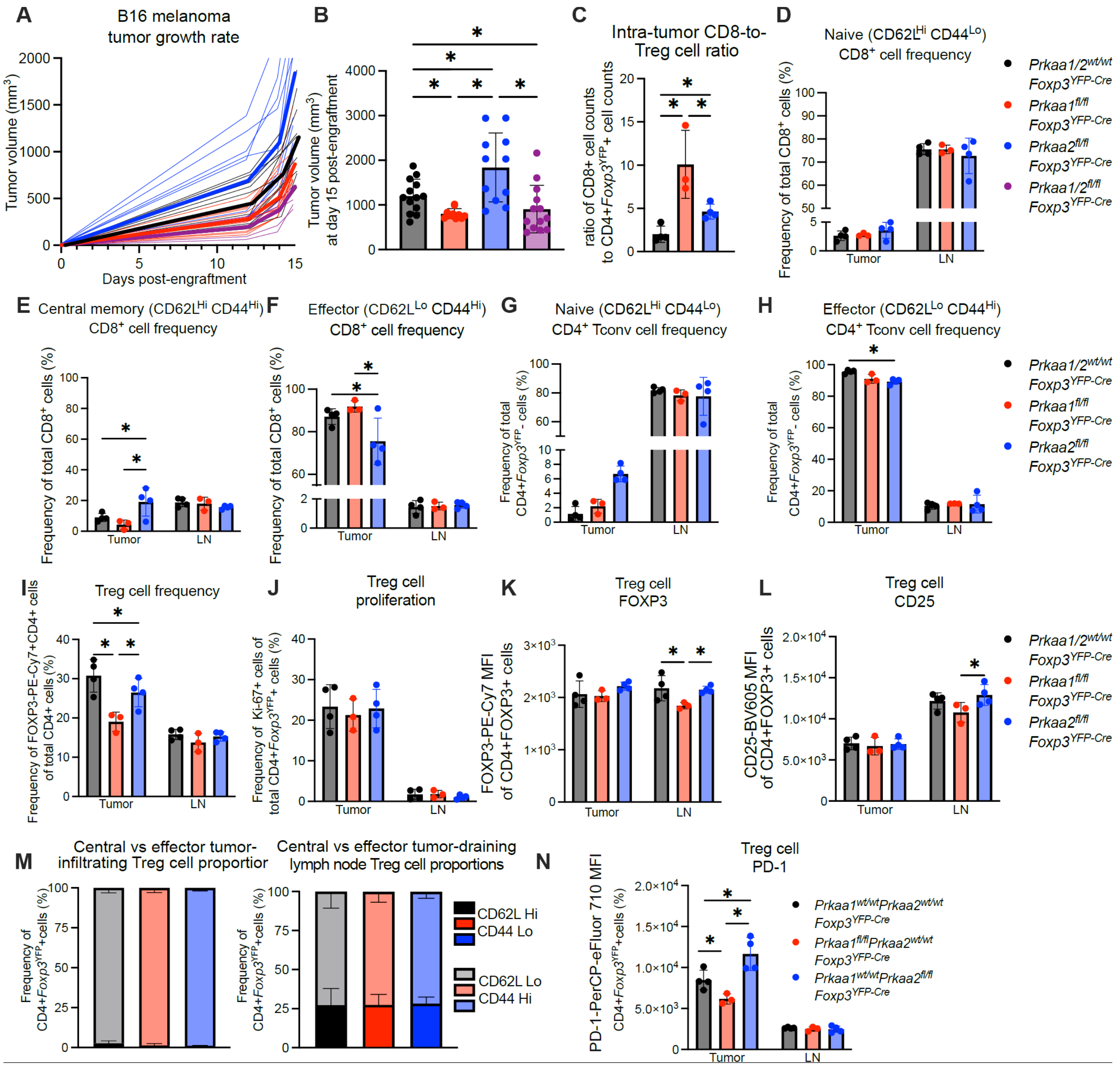

Supplemental Figure 4. The AMPKα1 and AMPKα2 isoforms have differential contribution to Treg cell suppressive function in the TME. (**A-B**)Growth of B16 melanoma tumors (A) and tumor volume at day 15 post-engraftment (B) of $Prkaa1/2^{wt/wt}Foxp3^{YFP\text{-}Cre}$ mice (control, *n*=20; includes replicates from Figure 2A-B), $Prkaa1^{fl/fl}Foxp3^{YFP\text{-}Cre}$ mice (*n*=10), $Prkaa2^{fl/fl}Foxp3^{YFP\text{-}Cre}$ mice (*n*=11), and $Prkaa1/2^{fl/fl}Foxp3^{YFP\text{-}Cre}$ mice (*n*=17; includes replicates from Figure 2A-B). (**C**) Ratio of live CD8+ cell counts to live CD4+$Foxp3^{YFP}$+ cell counts in single-cell suspensions of B16 melanoma tumors harvested from the flanks of control (*n*=4), $Prkaa1^{fl/fl}Foxp3^{YFP\text{-}Cre}$ mice (*n*=3), and $Prkaa2^{fl/fl}Foxp3^{YFP\text{-}Cre}$ mice (*n*=4) at day 15 post-engraftment. (**D-F**) Frequency of naive ($CD62L^{Hi}CD44^{Lo}$; D), central memory ($CD62L^{Hi}CD44^{Hi}$; E), and effector ($CD62L^{Lo}CD44^{Hi}$; F) CD8+ conventional T (Tconv) cells out of total CD8+ cells in tumors and tumor-draining lymph nodes (LN; *n*=4 control, *n*=3 $Prkaa1^{fl/fl}Foxp3^{YFP\text{-}Cre}$, *n*=4 $Prkaa2^{fl/fl}Foxp3^{YFP\text{-}Cre}$). (**G-H**) Frequency of naïve (G) and effector (H) CD4+$Foxp3^{YFP}$- cells out of total

CD4+*Foxp3*^{YFP}- cells in tumors and LN (*n*=4 control, *n*=3 *Prkaa1*$^{fl/fl}$*Foxp3*$^{YFP-Cre}$, *n*=4 *Prkaa2*$^{fl/fl}$*Foxp3*$^{YFP-Cre}$). (**I**) Frequency of CD4+*Foxp3*^{YFP}+ cells out of total CD4+ cells in tumors and LN (*n*=4 control, *n*=3 *Prkaa1*$^{fl/fl}$*Foxp3*$^{YFP-Cre}$, *n*=4 *Prkaa2*$^{fl/fl}$*Foxp3*$^{YFP-Cre}$). (**J**) Frequency of Ki-67+CD4+*Foxp3*^{YFP}+ cells out of total CD4+*Foxp3*^{YFP}+ cells in tumors and LN (*n*=4 control, *n*=3 *Prkaa1*$^{fl/fl}$*Foxp3*$^{YFP-Cre}$, *n*=4 *Prkaa2*$^{fl/fl}$*Foxp3*$^{YFP-Cre}$). (**K**-**L**) FOXP3-PE-Cy7 (K) and CD25-BV605 (L) mean fluorescence intensity (MFI) of CD4+FOXP3+ cells from tumors and LN (*n*=4 control, *n*=3 *Prkaa1*$^{fl/fl}$*Foxp3*$^{YFP-Cre}$, *n*=4 *Prkaa2*$^{fl/fl}$*Foxp3*$^{YFP-Cre}$). (**M**) Frequency of central (CD62L^{Hi}CD44^{Lo}) and effector (CD62L^{Lo}CD44^{Hi}) CD4+*Foxp3*^{YFP}+ cells out of total CD4+*Foxp3*^{YFP}+ cells in tumors and LN (*n*=4 control, *n*=3 *Prkaa1*$^{fl/fl}$*Foxp3*$^{YFP-Cre}$, *n*=4 *Prkaa2*$^{fl/fl}$*Foxp3*$^{YFP-Cre}$). (**N**) PD-1-BB700 MFI of CD4+*Foxp3*^{YFP}+ cells in tumors and LN (*n*=4 control, *n*=3 *Prkaa1*$^{fl/fl}$*Foxp3*$^{YFP-Cre}$, *n*=4 *Prkaa2*$^{fl/fl}$*Foxp3*$^{YFP-Cre}$). * $q < 0.05$ according to two-way ANOVA of day 15 data from (A) using the two-stage linear step-up procedure of Benjamini, Krieger, and Yekutieli with Q = 5% (B). * $q < 0.05$ according to one-way ANOVA using the two-stage linear step-up procedure of Benjamini, Krieger, and Yekutieli with Q = 5% (C-N). Three outliers were identified and excluded from (B) using the ROUT method (Q=1%).

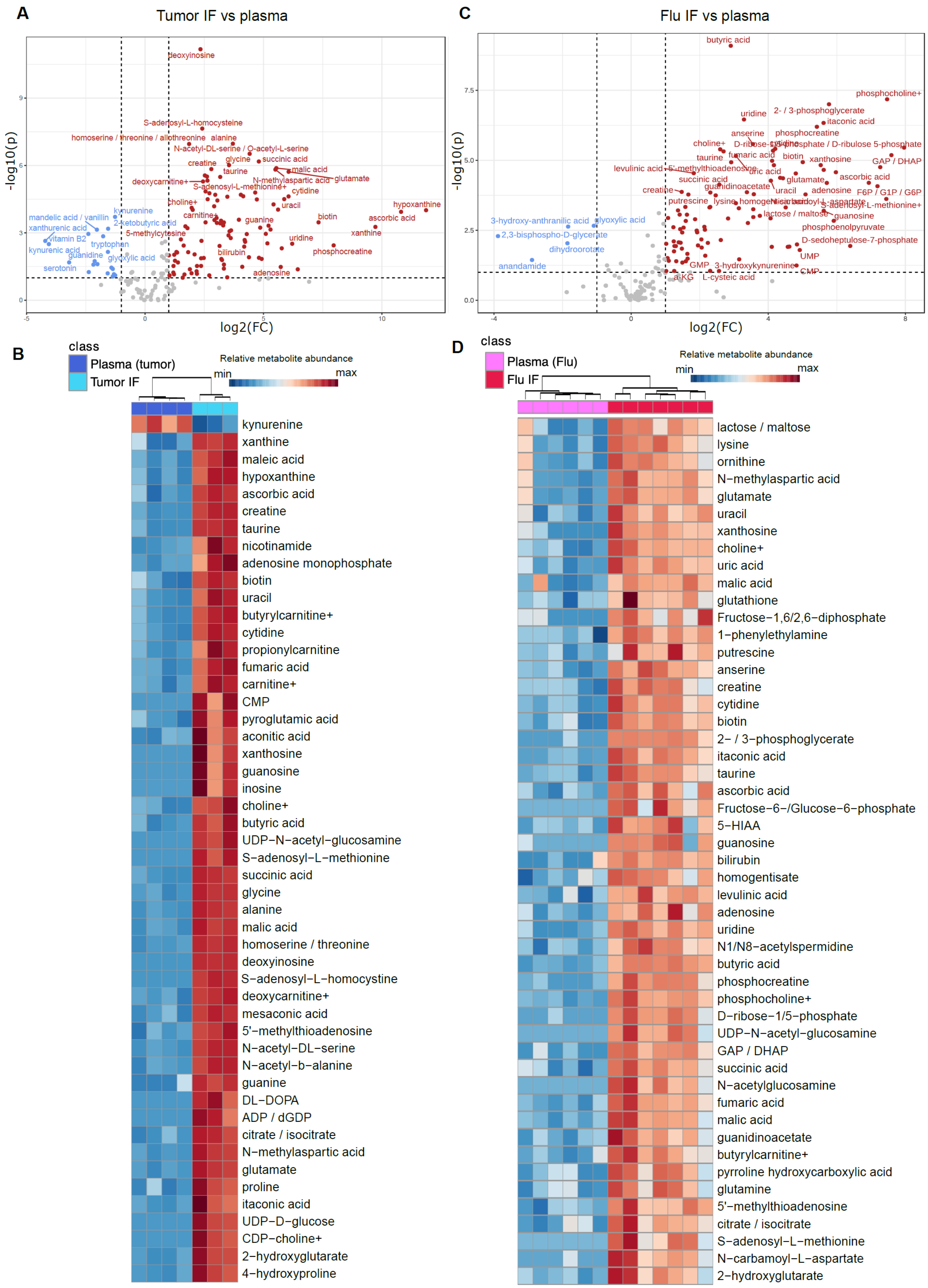

Supplemental Figure 5. Tumor and influenza virus-infected lung interstitial fluid metabolite abundance compared with plasma. (**A**) Volcano plot of abundance of metabolites detected in tumor

(n=3) interstitial fluid (IF) and paired plasma (n=4). Features with p < 0.1 were noted in red if $\log_2$(fold-change) ≥ 1.5 or blue if $\log_2$(fold-change) ≤ -1.5 when comparing tumor IF versus plasma. (**B**) Heatmap of top 50 differentially represented metabolites between tumor IF versus plasma. (**C**) Volcano plot of abundance of metabolites detected in influenza virus-infected lung (flu, n=7) IF and paired plasma (n=6). Features with p < 0.1 were noted in red if $\log_2$(fold-change) ≥ 1.5 or blue if $\log_2$(fold-change) ≤ -1.5 when comparing flu IF versus plasma. (**D**) Heatmap of top 50 differentially represented metabolites between flu IF versus plasma.

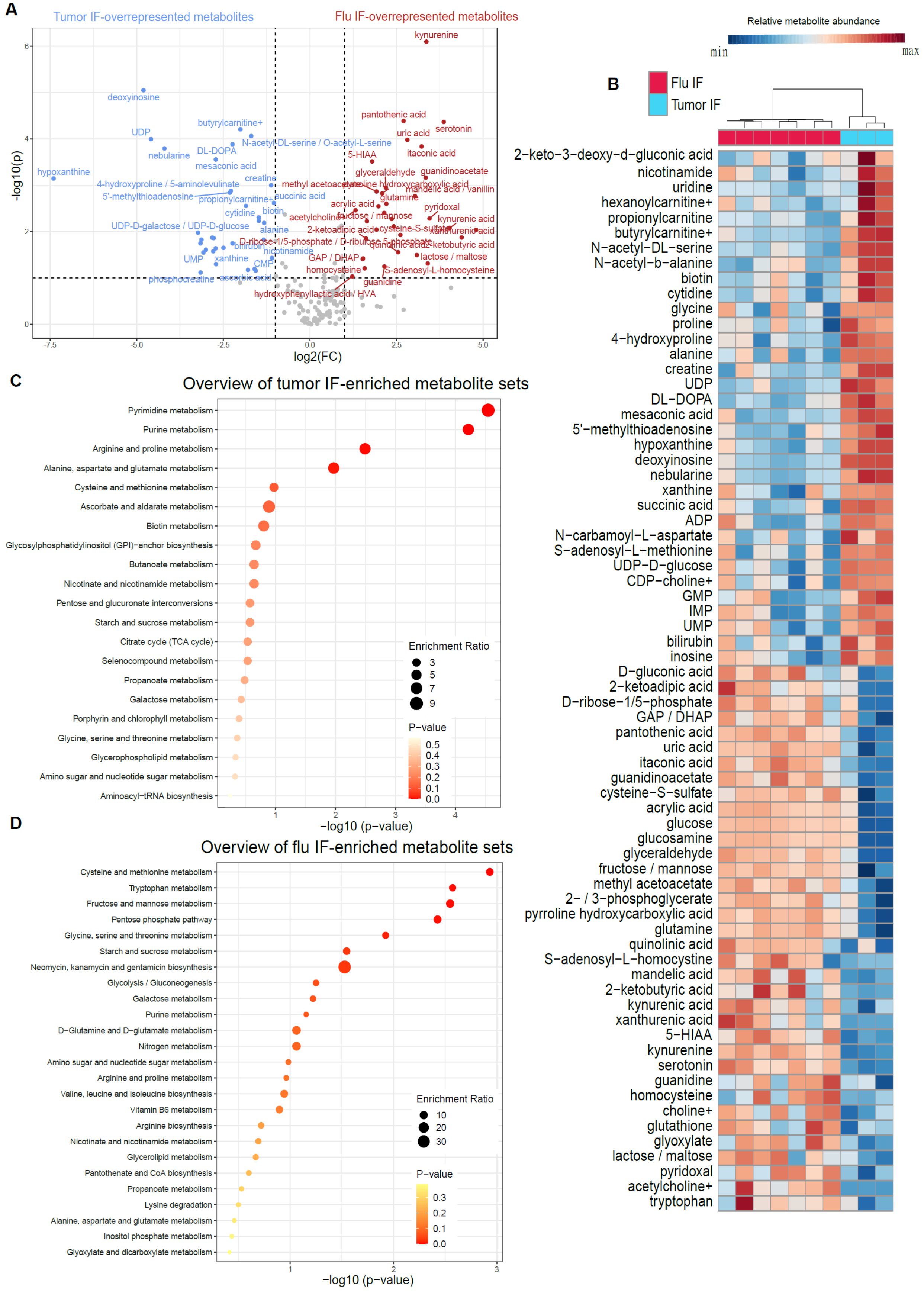

Supplemental Figure 6. Comparison of tumor interstitial fluid and influenza virus-infected lung interstitial fluid metabolite abundance. (**A**) Volcano plot of abundance of metabolites detected in

tumor (n=3) interstitial fluid (IF) and influenza virus-infected lung (flu, n=7) IF. Features with $p < 0.1$ were noted in red if $\log_2$(fold-change) ≥ 1.5 or blue if $\log_2$(fold-change) ≤ -1.5 when comparing flu IF versus tumor IF. (**B**) Heatmap of top 50 differentially represented metabolites between flu IF and tumor IF. (**C**) Overrepresentation analysis of significantly ($p < 0.1$) overrepresented metabolites in tumor IF relative to flu IF (**D**) Overrepresentation analysis of significantly ($p < 0.1$) overrepresented metabolites in flu IF relative to tumor IF.

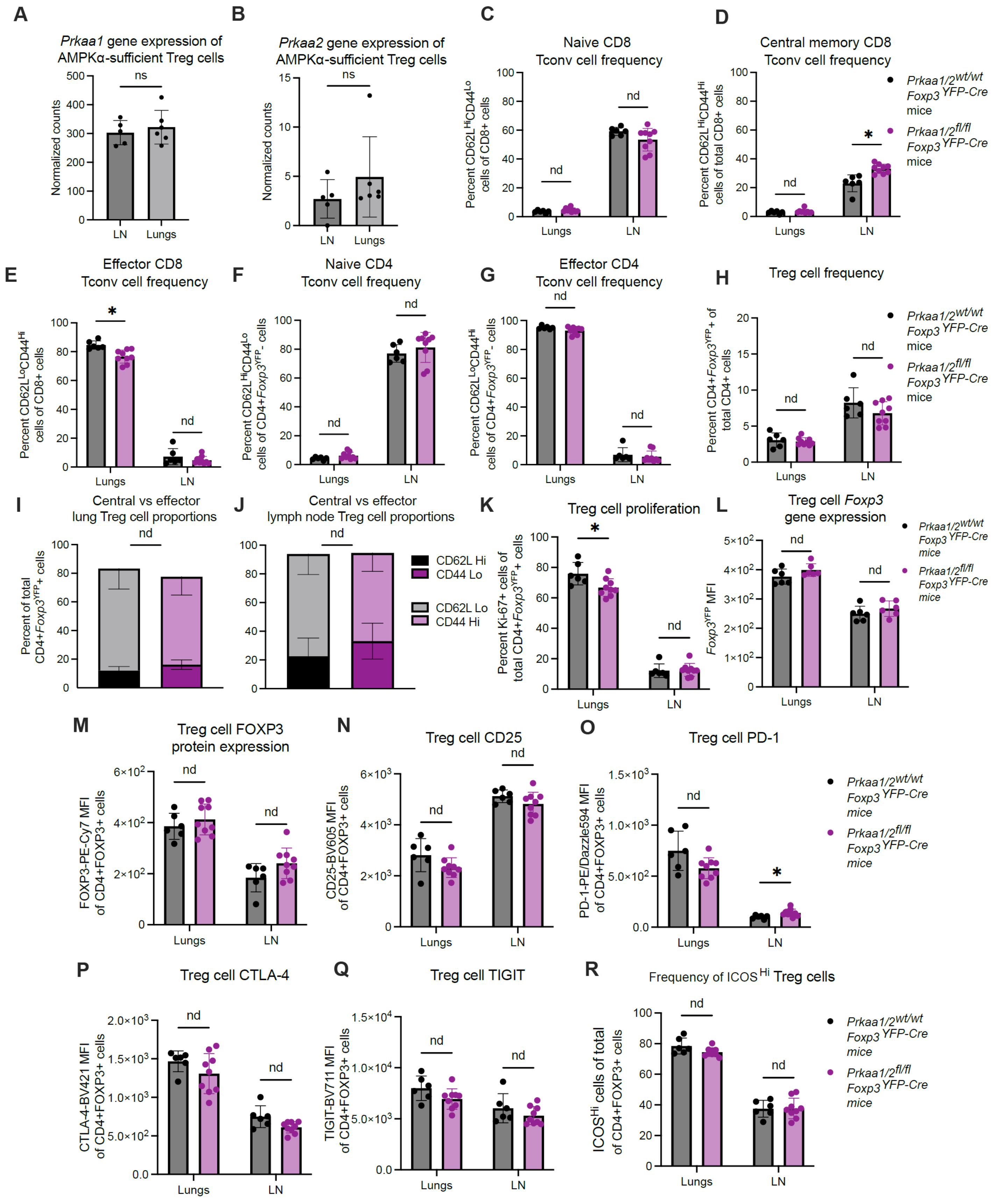

Supplemental Figure 7. Phenotyping of AMPKα-sufficient and -deficient Treg cells in the lung during viral pneumonia. (**A-B**) Normalized gene expression counts of *Prkaa1* (A) and *Prkaa2* (B) in

CD4+*Foxp3*^{YFP}+ cells sorted from axillary lymph nodes (LN; *n*=5) and lungs (*n*=6) of *Prkaa1/2*$^{wt/wt}$*Foxp3*$^{YFP-Cre}$ (control) mice at day 10 following intra-tracheal inoculation of influenza A/WSN/33 H1N1 (influenza) virus. (**C-E**) Frequency of naive (CD62L^{Hi}CD44^{Lo}; C), central memory (CD62L^{Hi}CD44^{Hi}; D), and effector (CD62L^{Lo}CD44^{Hi}; E) CD8+ conventional T (Tconv) cells out of total CD8+ cells in lungs and LN (*n*=6 control, *n*=9 *Prkaa1/2*$^{fl/fl}$*Foxp3*$^{YFP-Cre}$ mice) at day 10 post-influenza virus inoculation. (**F-G**) Frequency of naive (F) and effector (G) CD4+ Tconv cells out of total CD4+*Foxp3*^{YFP}- cells in lungs and LN (*n*=6 control, *n*=9 *Prkaa1/2*$^{fl/fl}$*Foxp3*$^{YFP-Cre}$ mice) at day 10 post-influenza virus inoculation. (**H**) Frequency of CD4+*Foxp3*^{YFP}+ cells out of total CD4+ cells in lungs and LN (*n*=6 control, *n*=9 *Prkaa1/2*$^{fl/fl}$*Foxp3*$^{YFP-Cre}$ mice) at day 10 post-influenza virus inoculation. (**I-J**) Frequency of central (CD62L^{Hi}CD44^{Lo}) and effector (CD62L^{Lo}CD44^{Hi}) CD4+*Foxp3*^{YFP}+ cells out of total CD4+Foxp3+ cells in lungs (I) and LN (J; *n*=6 control, *n*=9 *Prkaa1/2*$^{fl/fl}$*Foxp3*$^{YFP-Cre}$ mice) at day 10 post-influenza virus inoculation. (**K**) Frequency of Ki-67+CD4+*Foxp3*^{YFP}+ cells out of total CD4+*Foxp3*^{YFP}+ cells in lungs and LN (*n*=6 control, *n*=9 *Prkaa1/2*$^{fl/fl}$*Foxp3*$^{YFP-Cre}$ mice) at day 10 post-influenza virus inoculation. (**L**) *Foxp3*^{YFP} mean fluorescence intensity (MFI) of CD4+*Foxp3*^{YFP}+ cells in lungs and LN (*n*=6 control, *n*=6 *Prkaa1/2*$^{fl/fl}$*Foxp3*$^{YFP-Cre}$ mice) at day 10 post-influenza virus inoculation. (**M-Q**) FOXP3-PE-Cy7 (M), CD25-BV605 (N), PD-1-PE/Dazzle594 (O), CTLA-4-BV421 (P) and TIGIT-BV711 (Q) MFI of CD4+FOXP3+ cells from lungs and LN (*n*=6 control, *n*=9 *Prkaa1/2*$^{fl/fl}$*Foxp3*$^{YFP-Cre}$) at day 10 post-influenza virus inoculation. (**R**) Frequency of ICOS^{Hi} CD4+FOXP3+ cells of total CD4+FOXP3+ cells in lungs and LN (*n*=6 control, *n*=9 *Prkaa1/2*$^{fl/fl}$*Foxp3*$^{YFP-Cre}$) at day 10 post-influenza virus inoculation. * $q < 0.05$, ns not significant, nd no discovery according to Mann Whitney U test (A-B) with two-stage linear step-up procedure of Benjamini, Krieger, and Yekutieli with Q = 5% (C-R).

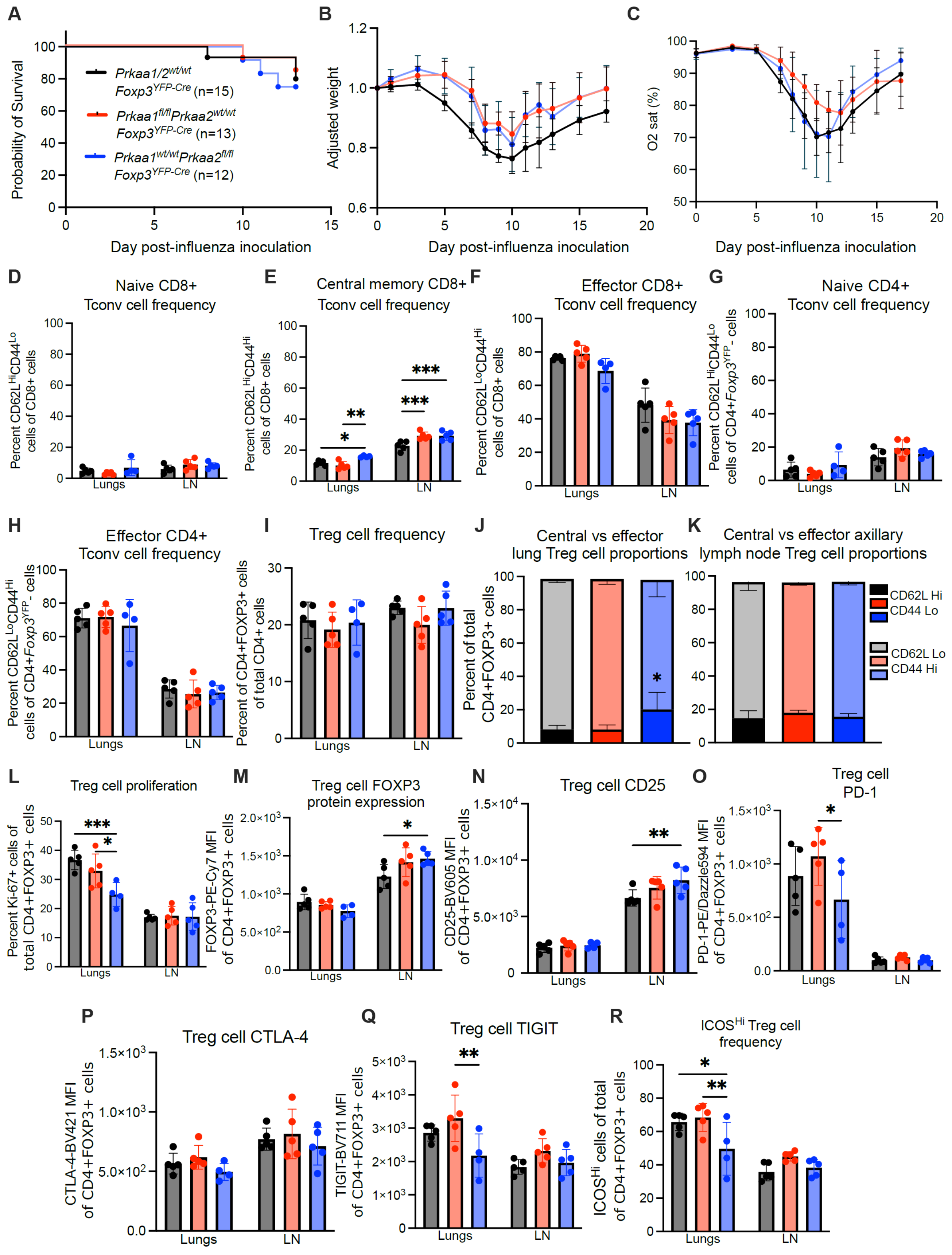

Supplemental Figure 8. Phenotyping of AMPKα1/2-sufficient, α1-deficient, and α2-deficient Treg cells in the lung during viral pneumonia. (**A**) Survival of control (*n*=8), *Prkaa1*$^{fl/fl}$*Foxp3*$^{YFP\text{-}Cre}$ (*n*=7),

and *Prkaa2*$^{fl/fl}$*Foxp3*$^{YFP\text{-}Cre}$ (*n*=6) mice following intra-tracheal inoculation of 12.5 plaque forming units (PFUs) of influenza A/WSN/33 H1N1 (influenza) virus. (**B**-**C**) Weight (B), and arterial oxyhemoglobin saturation (C) over time of control (*n*=6), *Prkaa1*$^{fl/fl}$*Foxp3*$^{YFP\text{-}Cre}$ (*n*=3), and *Prkaa2*$^{fl/fl}$*Foxp3*$^{YFP\text{-}Cre}$ (*n*=5) mice following intra-tracheal inoculation of 12.5 PFUs of influenza virus. (**D-F**) Frequency of naive (CD62L^{Hi}CD44^{Lo}; D), central memory (CD62L^{Hi}CD44^{Hi}; E), and effector (CD62L^{Lo}CD44^{Hi}; F) CD8+ conventional T (Tconv) cells out of total CD8+ cells in lungs and axillary lymph nodes (LN). (**G-H**) Frequency of naive (G) and effector (H) CD4+ Tconv cells out of total CD4+*Foxp3*^{YFP}- cells in lungs and LN. (**I**) Frequency of CD4+FOXP3+ cells out of total CD4+ cells in lungs and LN. (**J**-**K**) Frequency of central (CD62L^{Hi}CD44^{Lo}) and effector (CD62L^{Lo}CD44^{Hi}) CD4+FOXP3+ cells out of total CD4+FOXP3+ cells in lungs (J) and LN (K). * $p < 0.05$ versus control and *Prkaa1*$^{fl/fl}$*Foxp3*$^{YFP\text{-}Cre}$. (**L**) Frequency of Ki-67+CD4+FOXP3+ cells out of total CD4+FOXP3+ cells in lungs and LN. (**M**-**Q**) FOXP3-PE-Cy7 (M), CD25-BV605 (N), PD-1-PE/Dazzle594 (O), CTLA-4-BV421 (P) and TIGIT-BV711 (Q) MFI of CD4+FOXP3+ cells from lungs and LN. (**R**) Frequency of ICOS^{Hi} CD4+FOXP3+ cells of total CD4+FOXP3+ cells in lungs and LN. For D-R, *n*=5 lungs and LN for control mice, *n*=5 lungs and LN for *Prkaa1*$^{fl/fl}$*Foxp3*$^{YFP\text{-}Cre}$ mice, and *n*=4 lungs, *n*=5 LN for *Prkaa2*$^{fl/fl}$*Foxp3*$^{YFP\text{-}Cre}$ mice at day 10 post-influenza virus inoculation. Survival curve (A) *p* was determined using log-rank (Mantel-Cox) test. * $q < 0.05$, ** $q < 0.01$, *** $q < 0.001$, according to Mann Whitney U test with two-stage linear step-up procedure of Benjamini, Krieger, and Yekutieli with Q = 5% (B-R).

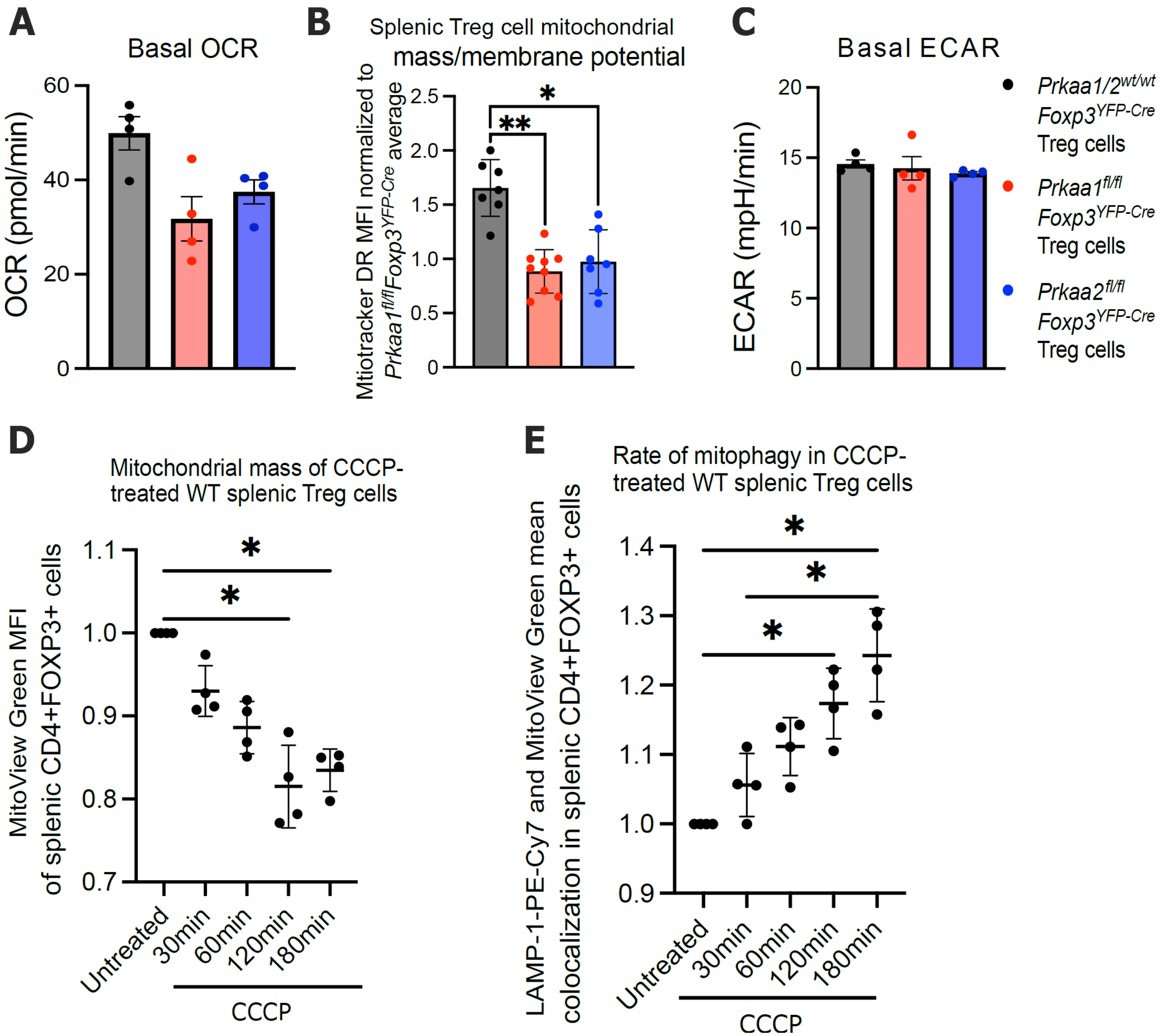

Supplemental Figure 9. Metabolic phenotyping of AMPKα1/2-sufficient, α1-deficient, and α2-deficient splenic Treg cells at homeostasis. (**A**) Basal oxygen consumption rate (OCR) of CD4+*Foxp3*^{YFP}+ splenocytes from control (*n*=4), *Prkaa1*$^{fl/fl}$*Foxp3*$^{YFP\text{-}Cre}$ (*n*=4), and *Prkaa2*$^{fl/fl}$*Foxp3*$^{YFP\text{-}Cre}$ (*n*=4) mice. (**B**) MitoTracker Deep Red (DR) mean fluorescence intensity (MFI) of CD4+*Foxp3*^{YFP}+ splenocytes at homeostasis (*n*=7 control, *n*=9 *Prkaa1*$^{fl/fl}$*Foxp3*$^{YFP\text{-}Cre}$, *n*=7 Prkaa2$^{fl/fl}$*Foxp3*$^{YFP\text{-}Cre}$ mice). Data normalized to *Prkaa1*$^{fl/fl}$*Foxp3*$^{YFP\text{-}Cre}$ mice average across three independent experiments. (**C**) Basal extracellular acidification rate (ECAR) of CD4+*Foxp3*^{YFP}+ splenocytes (*n*=4 control, *n*=4 *Prkaa1*$^{fl/fl}$*Foxp3*$^{YFP\text{-}Cre}$, *n*=4 Prkaa2$^{fl/fl}$*Foxp3*$^{YFP\text{-}Cre}$ mice). (**D-E**) MitoView Green MFI (D) and mean LAMP-1-PE-Cy-7/MitoView Green co-localization (E) over time of CD4+FOXP3+ splenocytes from control mice (*n*=4) after in vitro exposure to CCCP (10 μM); data were normalized to untreated condition. * $p < 0.05$, ** $p < 0.01$ according to Kruskal-Wallis test with two-stage linear step-up procedure of Benjamini, Krieger, and Yekutieli with Q = 5%.

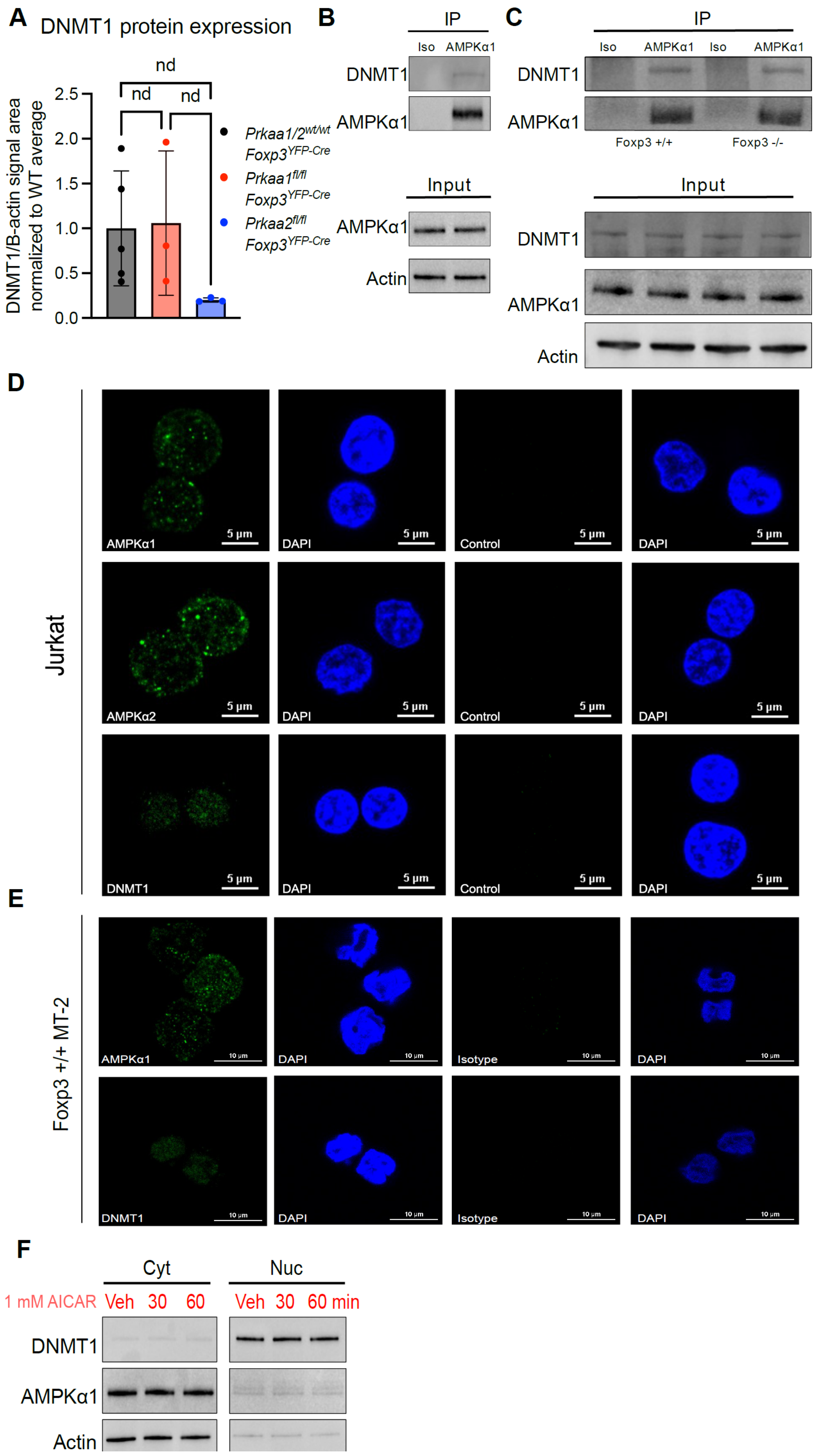

Supplemental Figure 10. AMPKα1 interacts with DNMT1 in Jurkat and MT-2 cells. (**A**) DNMT1 protein expression of splenic CD4+*Foxp3*^{YFP}+ (Treg) cells at homeostasis (*n*=5 control, *n*=3

Prkaa1$^{fl/fl}$*Foxp3*$^{YFP\text{-}Cre}$, *n*=3 *Prkaa2*$^{fl/fl}$*Foxp3*$^{YFP\text{-}Cre}$). Data from controls also shown in Figure 6D. DNMT1 peak intensity area was normalized to the corresponding sample's β-actin peak intensity area. (**B**-**C**) anti-AMPKα1 and isotype control immunoprecipitates from Jurkat cell (B) and MT-2 cell (C) lysates blotted for DNMT1 protein. (**D**-**E**) Representative microscopy images of Jurkat cells (D) and MT-2 cells (E) showing AMPKα1 and DNMT1 subcellular localization. (**F**) Immunoblots for DNMT1, AMPKα1, and β-actin on nuclear and cytoplasmic fractions of cell lysates obtained from AMPKα1/α2-sufficient ex vivo-induced (i)Treg cells treated with either vehicle, 30min AICAR 1mM, or 60min AICAR 1mM. nd no discovery according to Mann-Whitney *U* test with two-stage linear step-up procedure of Benjamini, Krieger, and Yekutieli with Q = 5% (A).

Supplemental data set 1. Peak intensity data of annotated metabolites detected in AMPKα1/α2-deficient and -sufficient splenic Treg cells of 12–15-week-old mice at homeostasis.

Supplemental data set 2. Differentially expressed genes detected when comparing AMPKα1/α2-deficient versus -sufficient splenic Treg cells of 12–15-week-old mice at homeostasis and their corresponding k-means cluster.

Supplemental data set 3. Differentially expressed genes detected when comparing AMPKα1/α2-deficient versus -sufficient B16 melanoma tumor-infiltrating Treg cells of 12–15-week-old mice (15 days after subcutaneous engraftment) and their corresponding k-means cluster.

Supplemental data set 4. Differentially expressed genes detected when comparing AMPKα1/α2-deficient versus -sufficient B16 melanoma tumor-infiltrating Treg cells of 12–15-week-old mice (12 days after subcutaneous engraftment) and their corresponding k-means cluster.

Supplemental data set 5. Differentially expressed genes detected when comparing AMPKα1-deficient versus -sufficient B16 melanoma tumor-infiltrating Treg cells of 12–15-week-old mice (12 days after subcutaneous engraftment) and their corresponding k-means cluster.

Supplemental data set 6. Differentially expressed genes detected when comparing AMPKα2-deficient versus -sufficient B16 melanoma tumor-infiltrating Treg cells of 12–15-week-old mice (12 days after subcutaneous engraftment) and their corresponding k-means cluster.

Supplemental data set 7. Peak intensity data of annotated metabolites detected in the interstitial fluid of lungs from influenza virus-infected mice (10 days post-inoculation), the interstitial fluid of B16 melanoma tumors (15 days after subcutaneous engraftment), and paired plasma samples.

Supplemental data set 8. Peak intensity data of annotated metabolites detected in AMPKα1/α2-deficient and -sufficient lung Treg cells from 12–15-week-old influenza virus-infected mice (10 days post-inoculation).
