# Supplementary material for: AMPK is necessary for Treg functional adaptation to microenvironmental stress during malignancy and viral pneumonia
Source: J Clin Invest. 2025 Mar 18;135(9):e179572. doi: 10.1172/JCI179572 (PMC12043082; doi:10.1172/JCI179572)

Full unedited blots

MATA et al 2023

JCI

Figure 6D: DNMT1 quantification nTreg WT vs AMPKα1/α2 double KO (DKO):

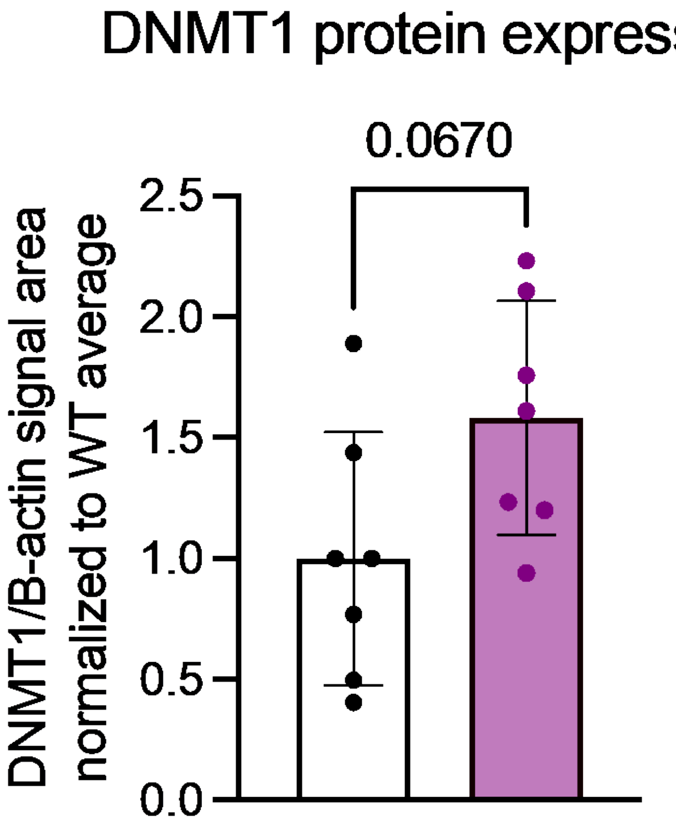

Three combined independent experiments:

- 01-24-2025 (*n*= 5 per group)
- 07-21-2022 (*n*=1 per group)
- 05-23-2022 (*n*=1 per group)

Figure 6D: DNMT1 quantification nTreg WT vs AMPKα1/α2 double KO (DKO): 01-24-2025 experiment

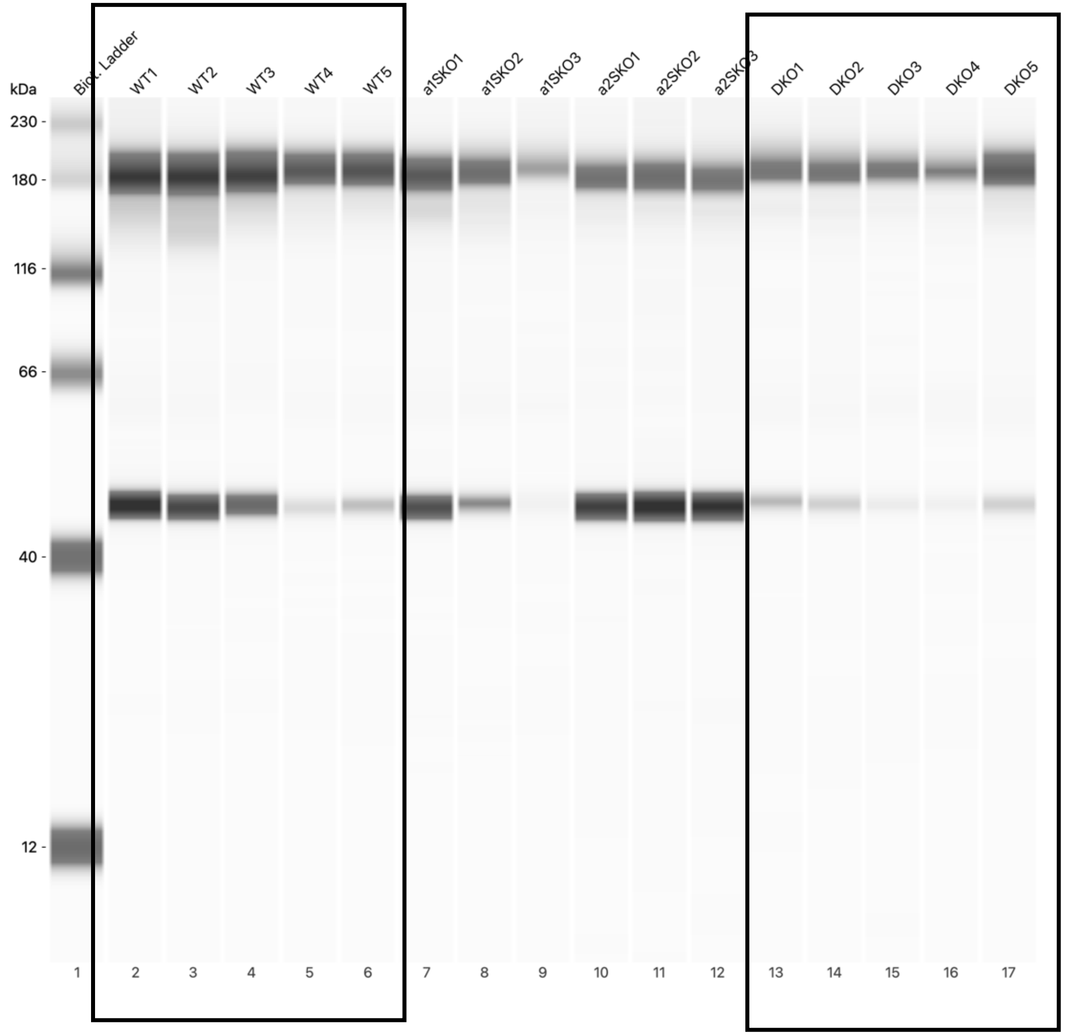

WT1-5 also shown in Supplemental Figure 10A

a1SKO1-3 and a2SKO1-3 are shown in Supplemental Figure 10A

Figure 6D: DNMT1 quantification nTreg WT vs AMPKα1/α2 double KO (DKO): 01-24-2025 experiment

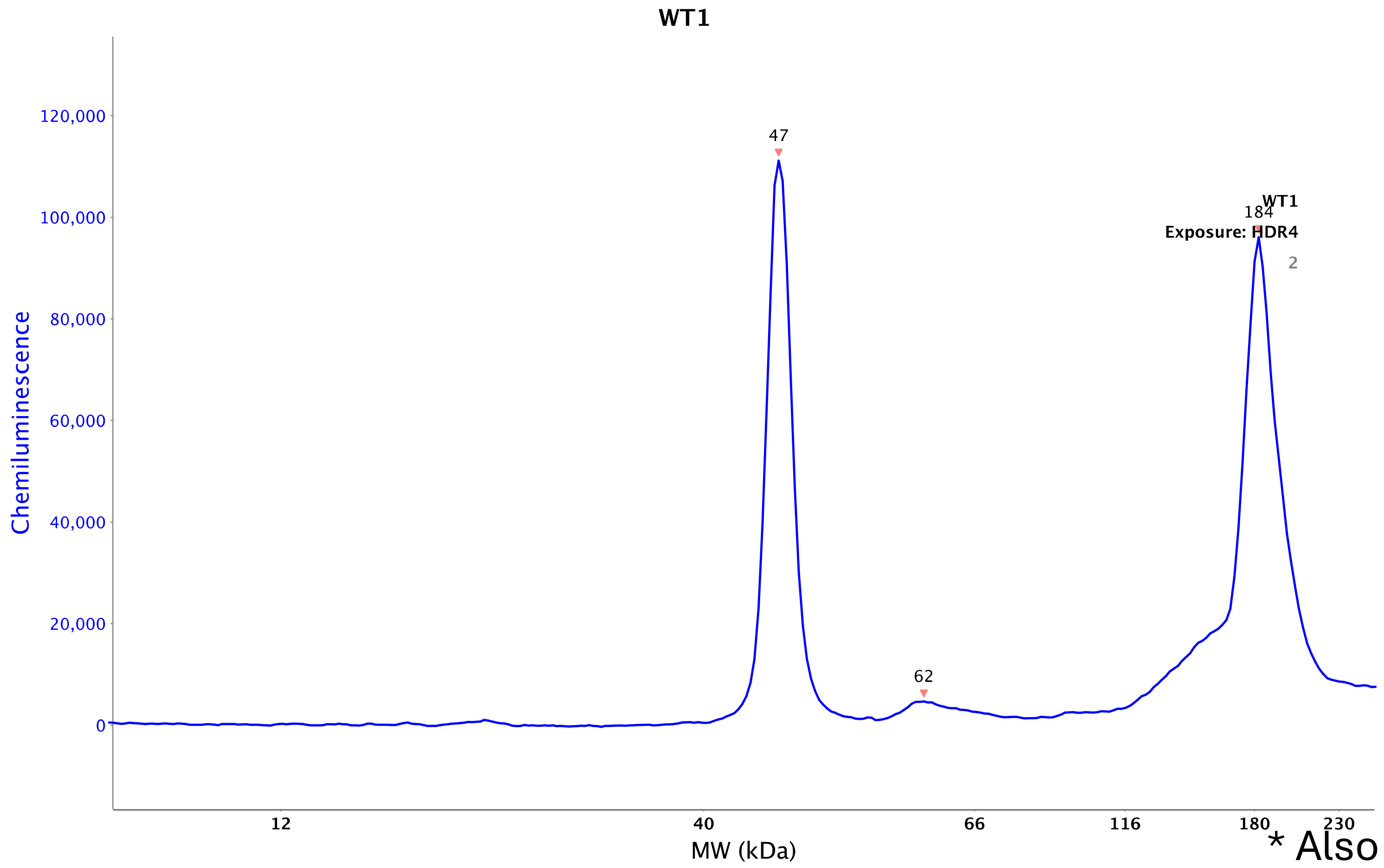

* Also shown in Supplemental Figure 10A

Figure 6D: DNMT1 quantification nTreg WT vs AMPKα1/α2 double KO (DKO): 01-24-2025 experiment

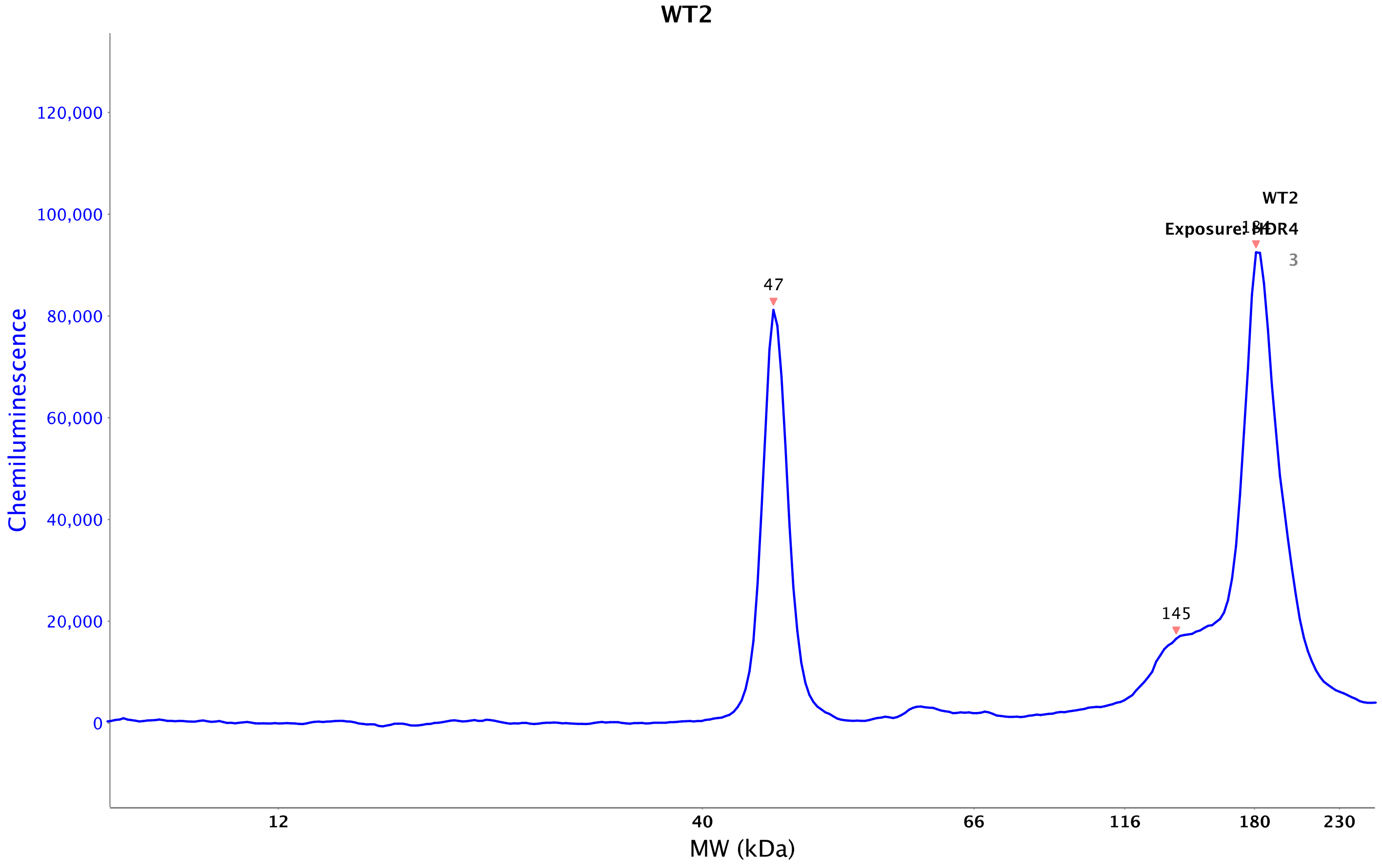

* Also shown in Supplemental Figure 10A

Figure 6D: DNMT1 quantification nTreg WT vs AMPKα1/α2 double KO (DKO): 01-24-2025 experiment

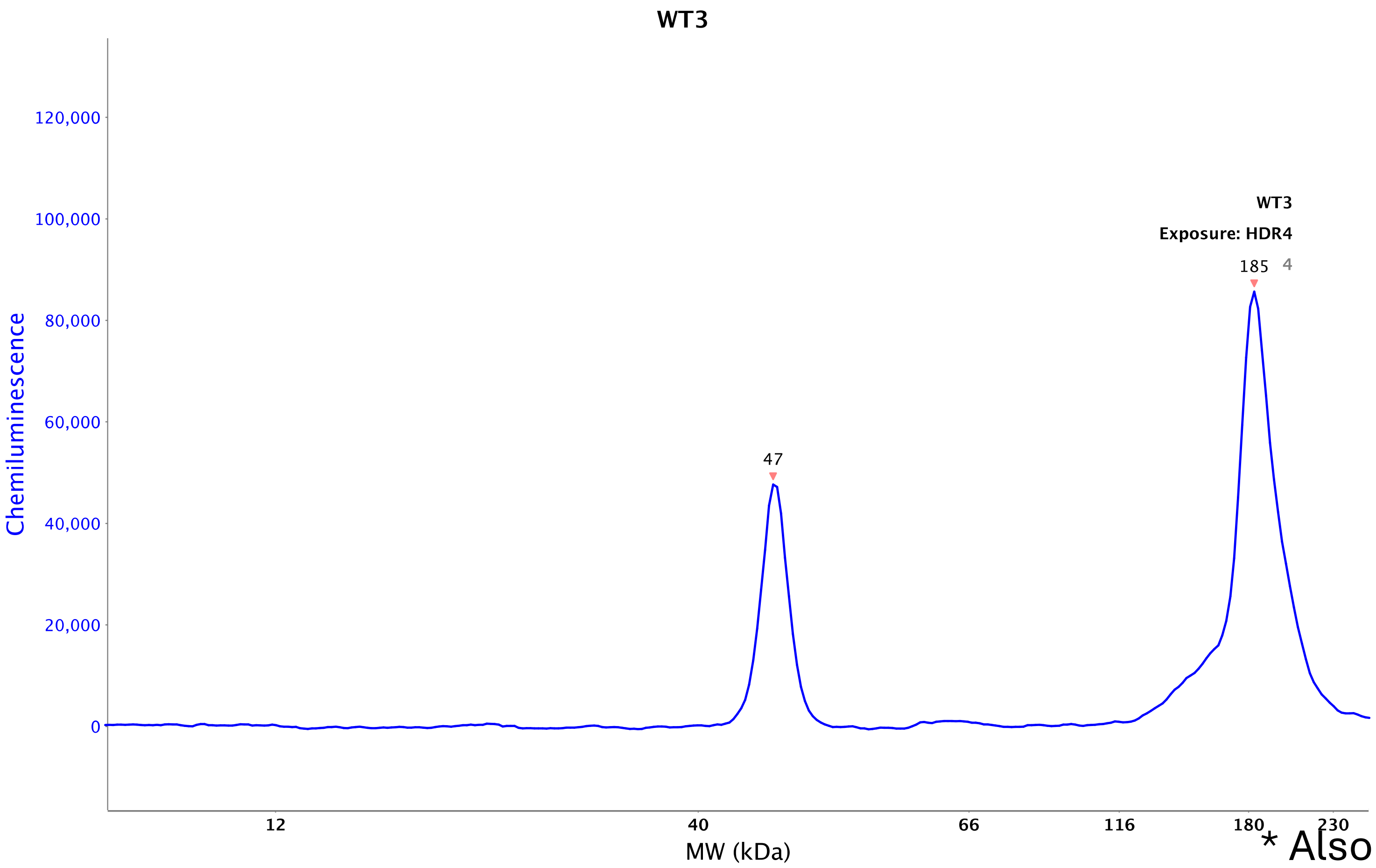

* Also shown in Supplemental Figure 10A

Figure 6D: DNMT1 quantification nTreg WT vs AMPKα1/α2 double KO (DKO): 01-24-2025 experiment

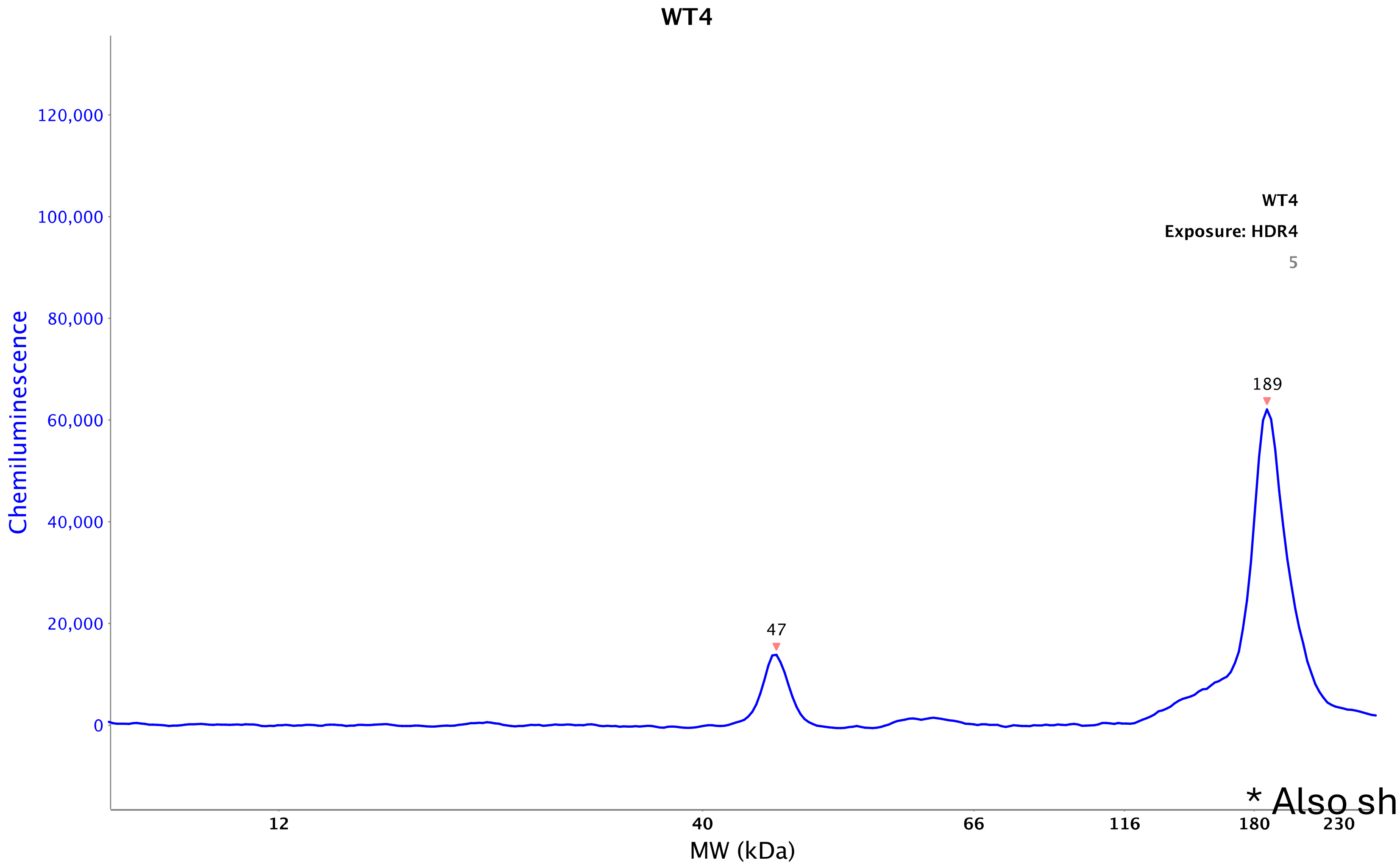

* Also shown in Supplemental Figure 10

Figure 6D: DNMT1 quantification nTreg WT vs AMPKα1/α2 double KO (DKO): 01-24-2025 experiment

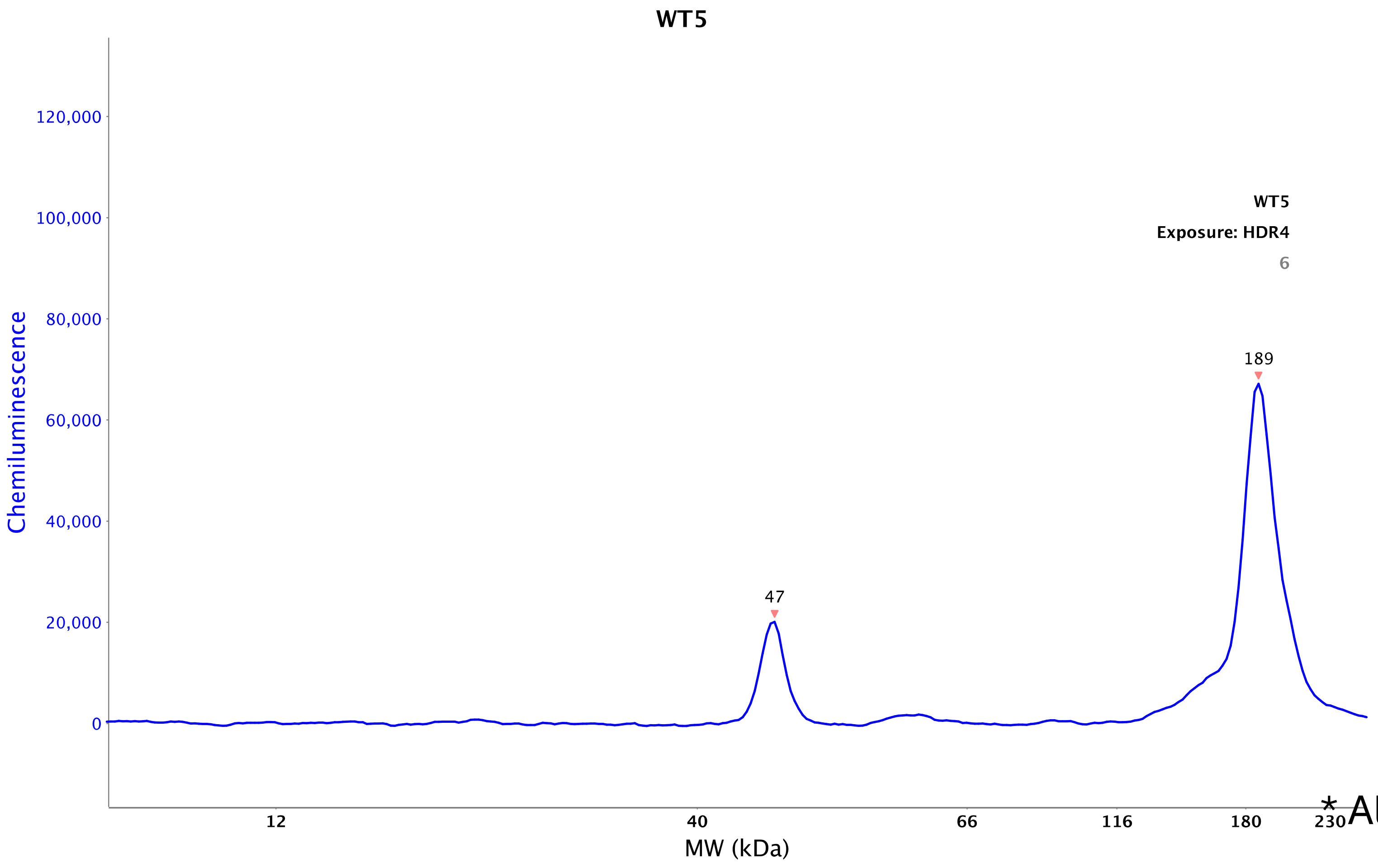

* Also shown in Supplemental Figure 10

Figure 6D: DNMT1 quantification nTreg WT vs AMPKα1/α2 double KO (DKO): 01-24-2025 experiment

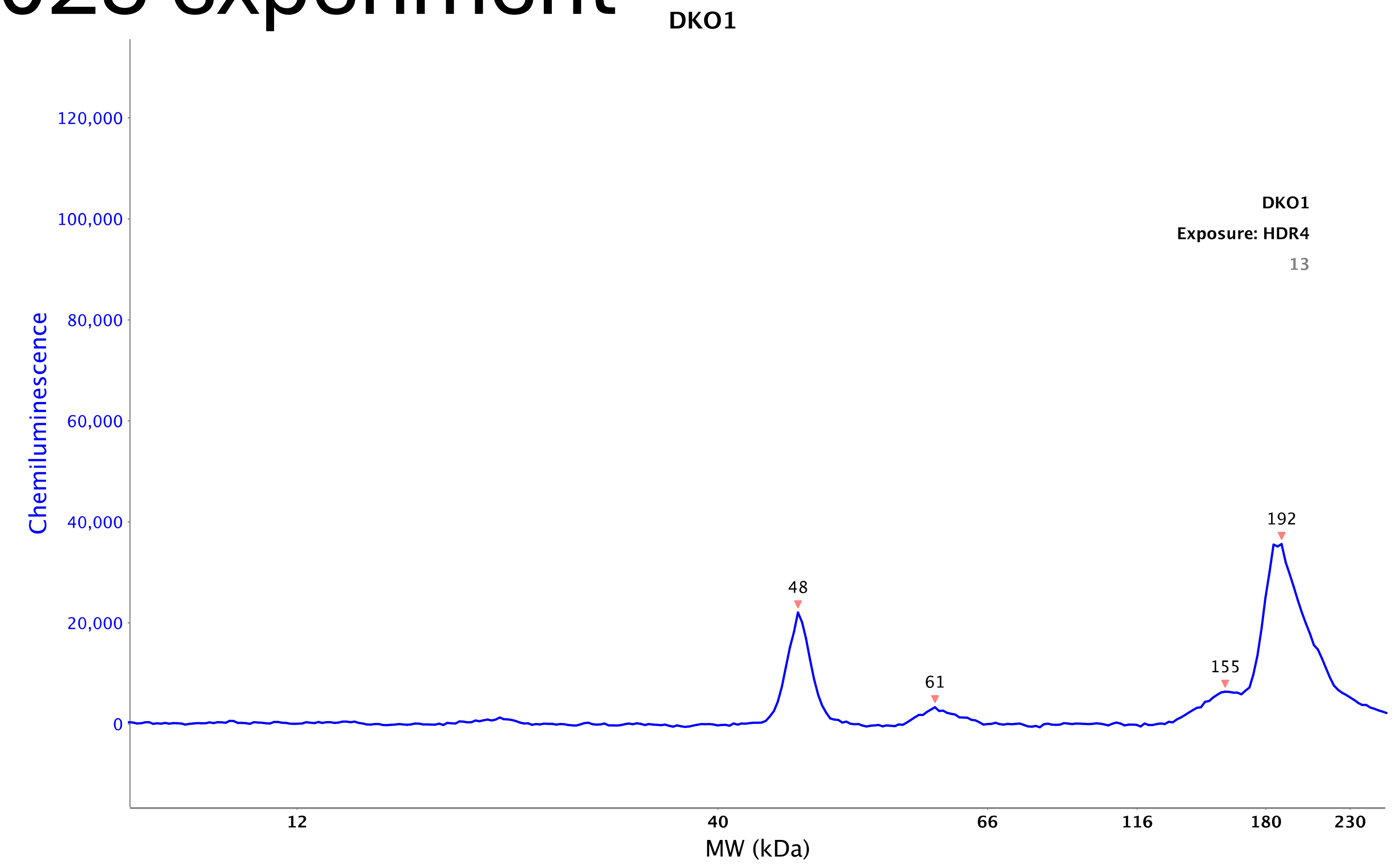

Figure 6D: DNMT1 quantification nTreg WT vs AMPKα1/α2 double KO (DKO): 01-24-2025 experiment

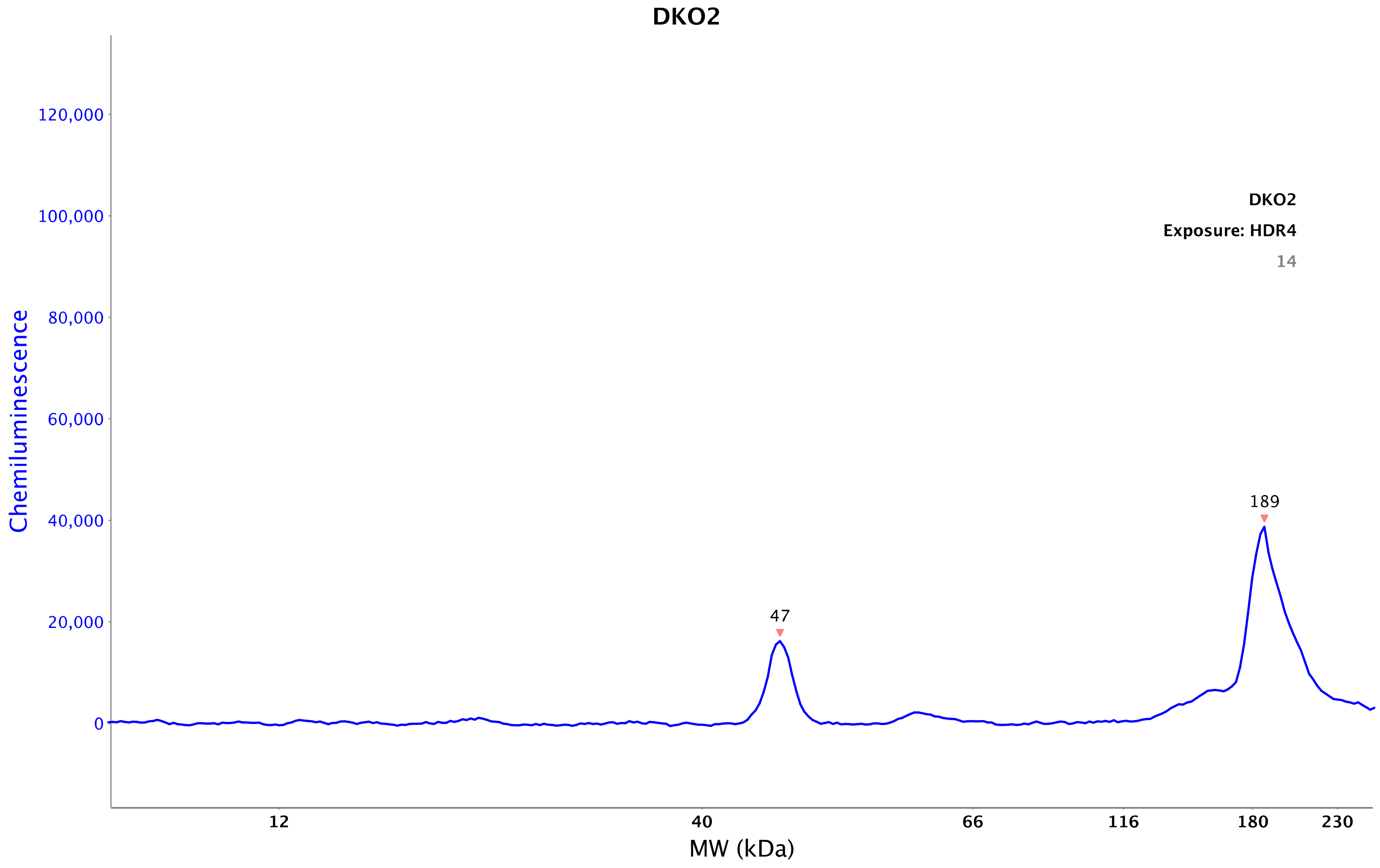

Figure 6D: DNMT1 quantification nTreg WT vs AMPKα1/α2 double KO (DKO): 01-24-2025 experiment

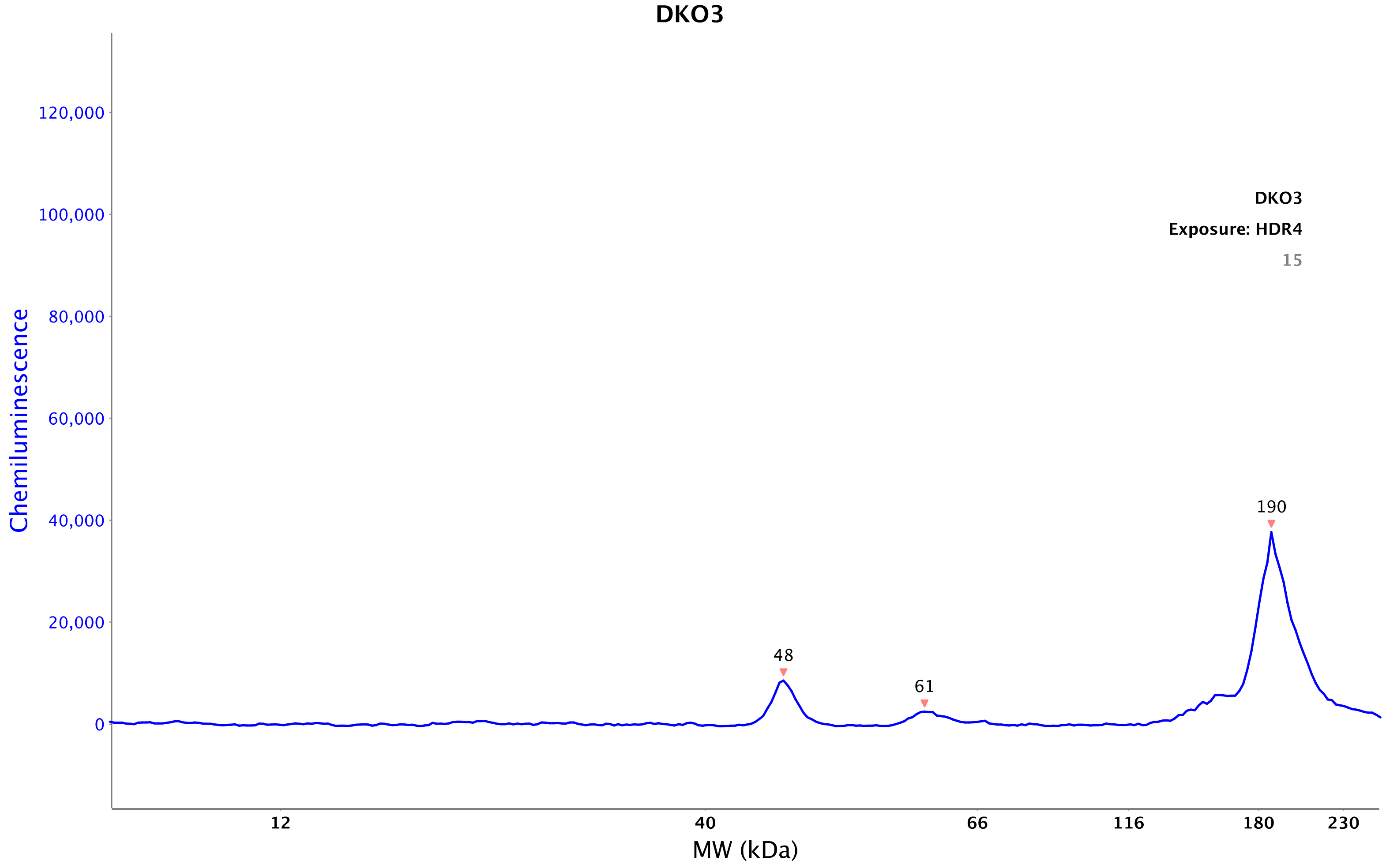

Figure 6D: DNMT1 quantification nTreg WT vs AMPKα1/α2 double KO (DKO): 01-24-2025 experiment

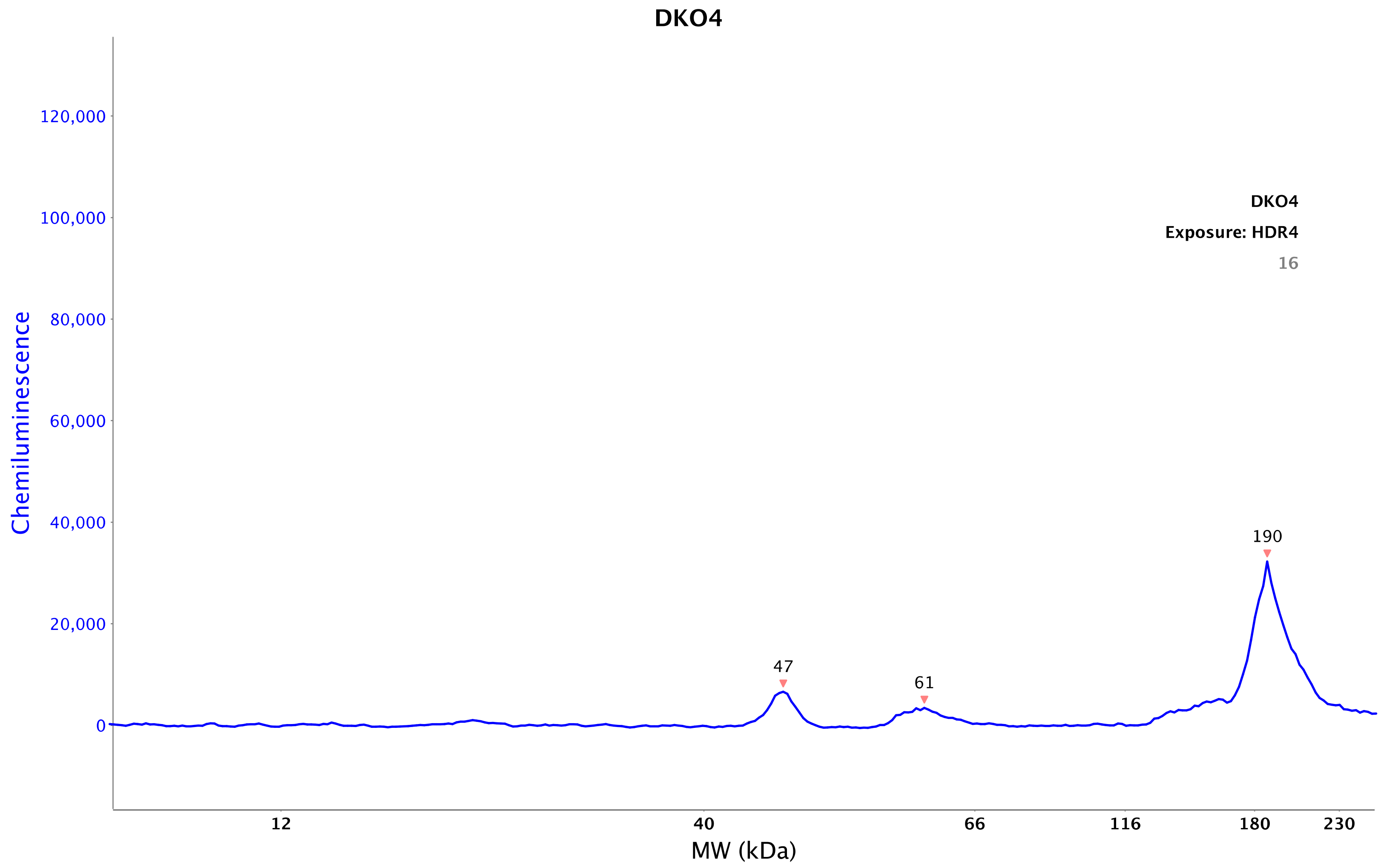

Figure 6D: DNMT1 quantification nTreg WT vs AMPKα1/α2 double KO (DKO): 01-24-2025 experiment

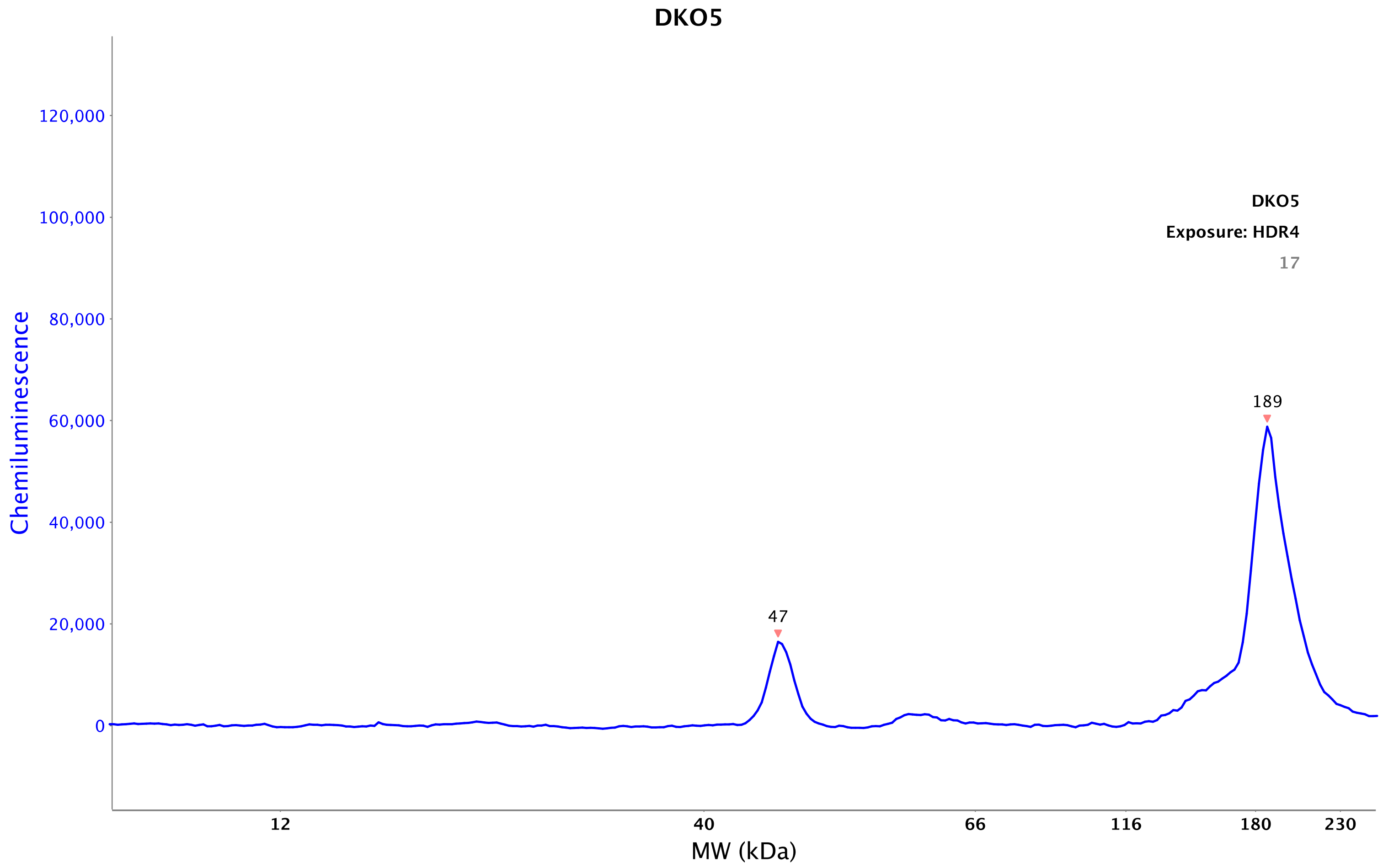

Figure 6D: DNMT1 quantification nTreg WT vs AMPKα1/α2 double KO (DKO): 07-21-2022 experiment

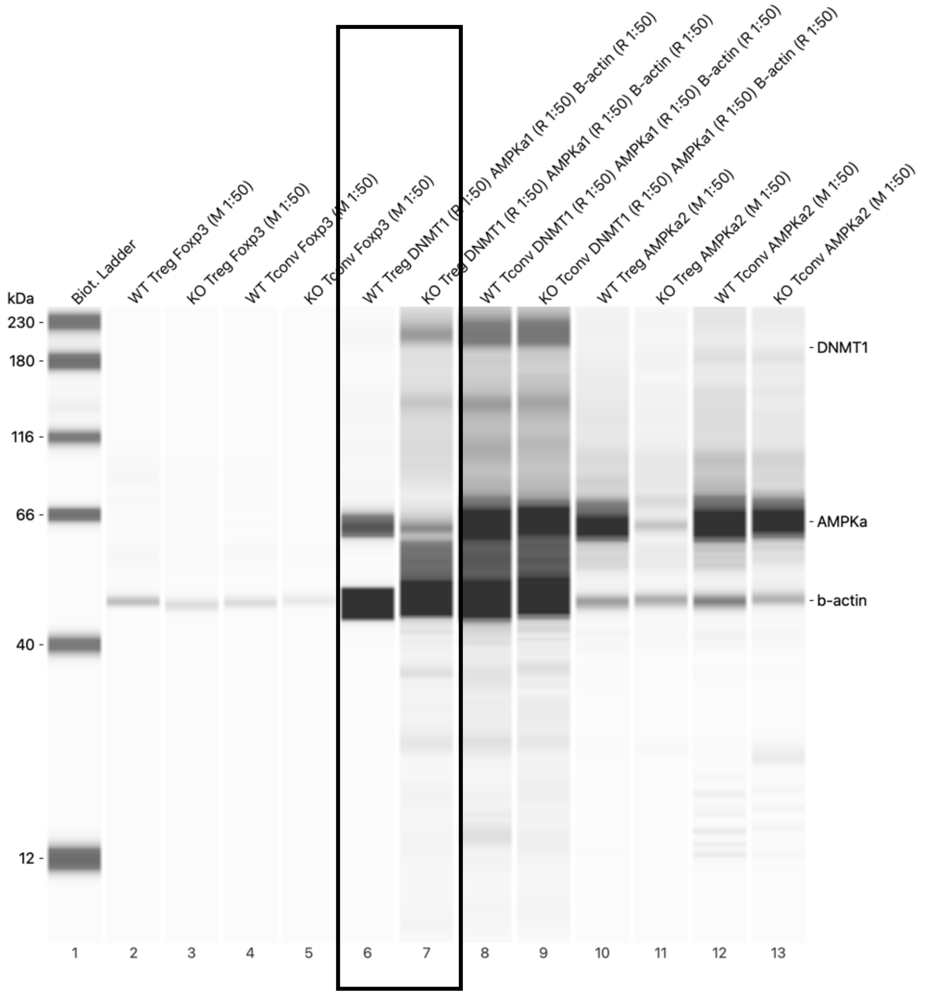

Only data from lanes 6 and 7 are included in Figure 6C

Figure 6D: DNMT1 quantification nTreg WT vs AMPKα1/α2 double KO (DKO): 07-21-2022 experiment

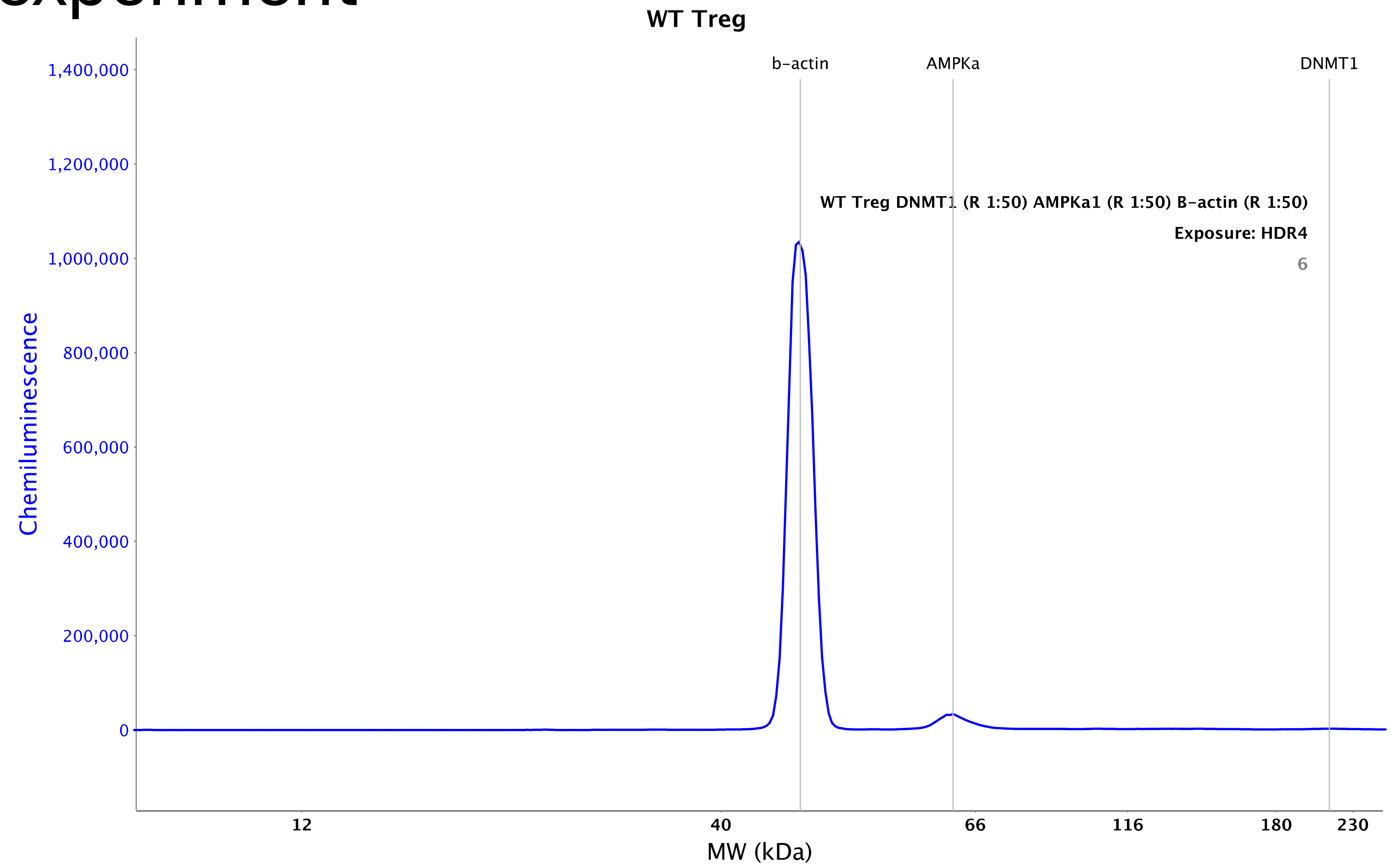

Figure 6D: DNMT1 quantification nTreg WT vs AMPKα1/α2 double KO (DKO): 07-21-2022 experiment

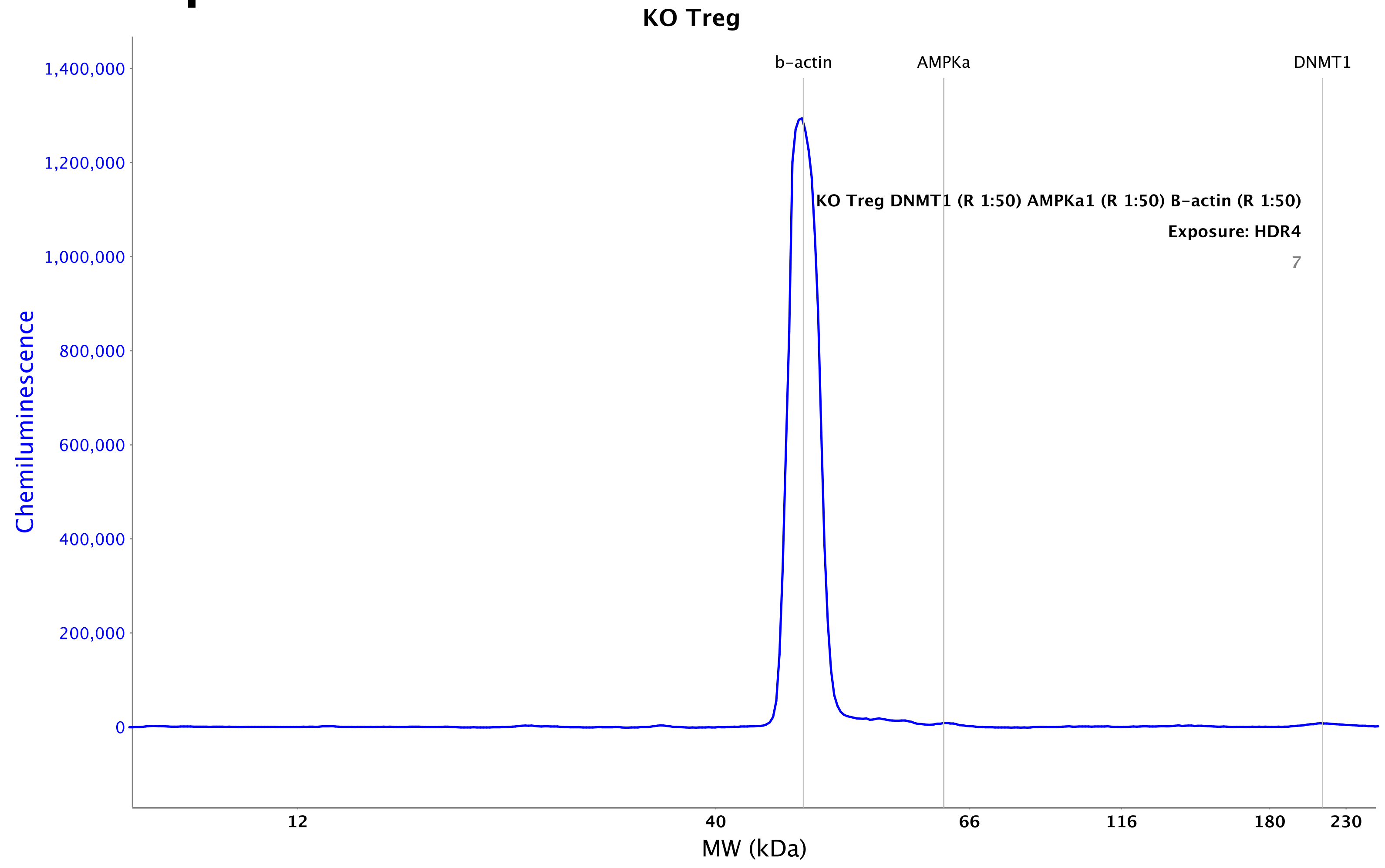

Figure 6D: DNMT1 quantification nTreg WT vs AMPKα1/α2 double KO (DKO): 05-23-2022 experiment

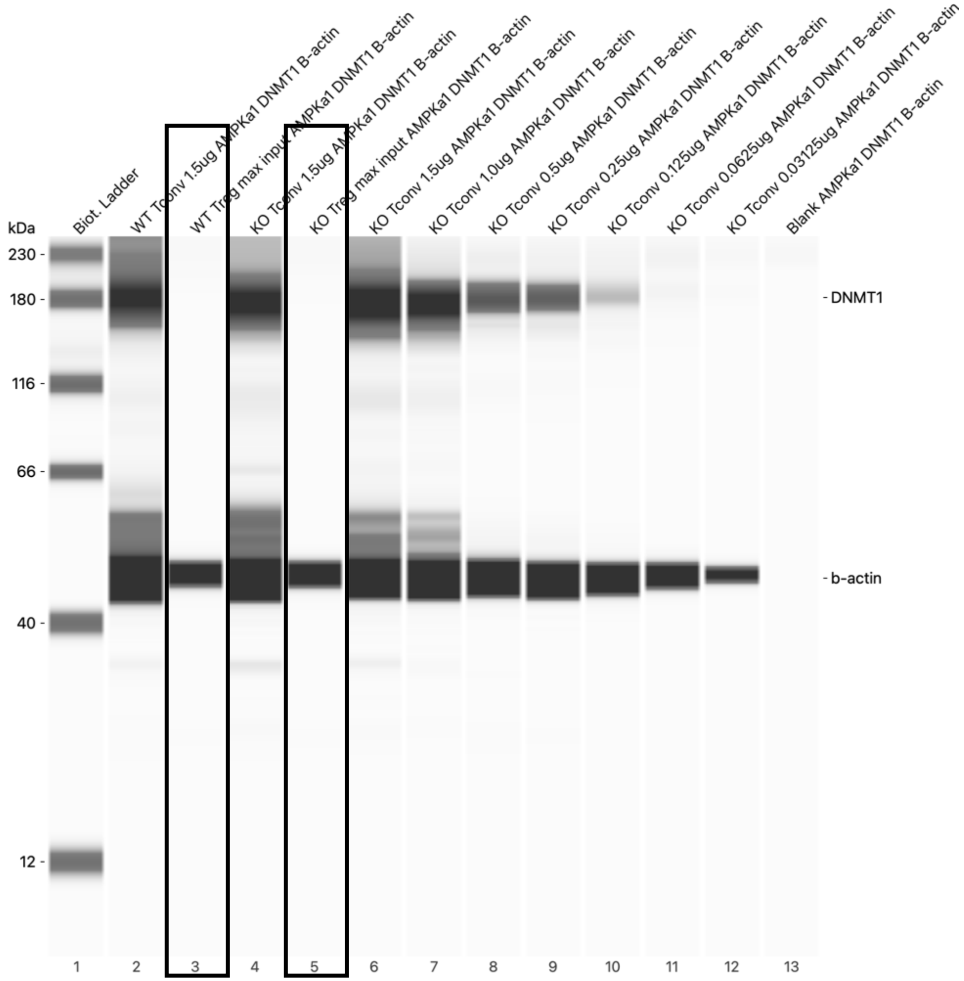

Only data from lanes 2 and 4 are included in Figure 6C

Figure 6D: DNMT1 quantification nTreg WT vs AMPKα1/α2 double KO (DKO): 05-23-2022 experiment

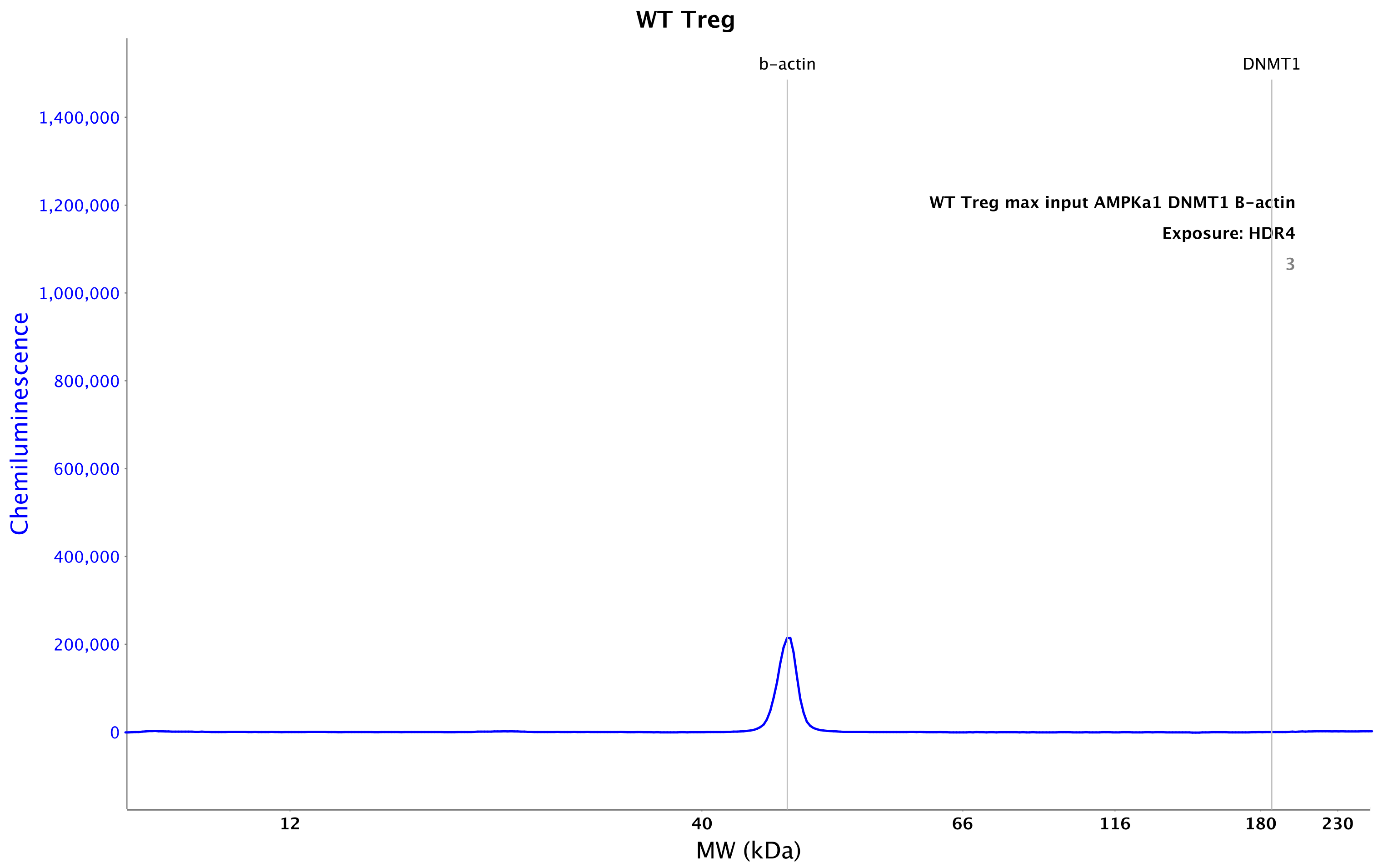

Figure 6D: DNMT1 quantification nTreg WT vs AMPKα1/α2 double KO (DKO): 05-23-2022 experiment

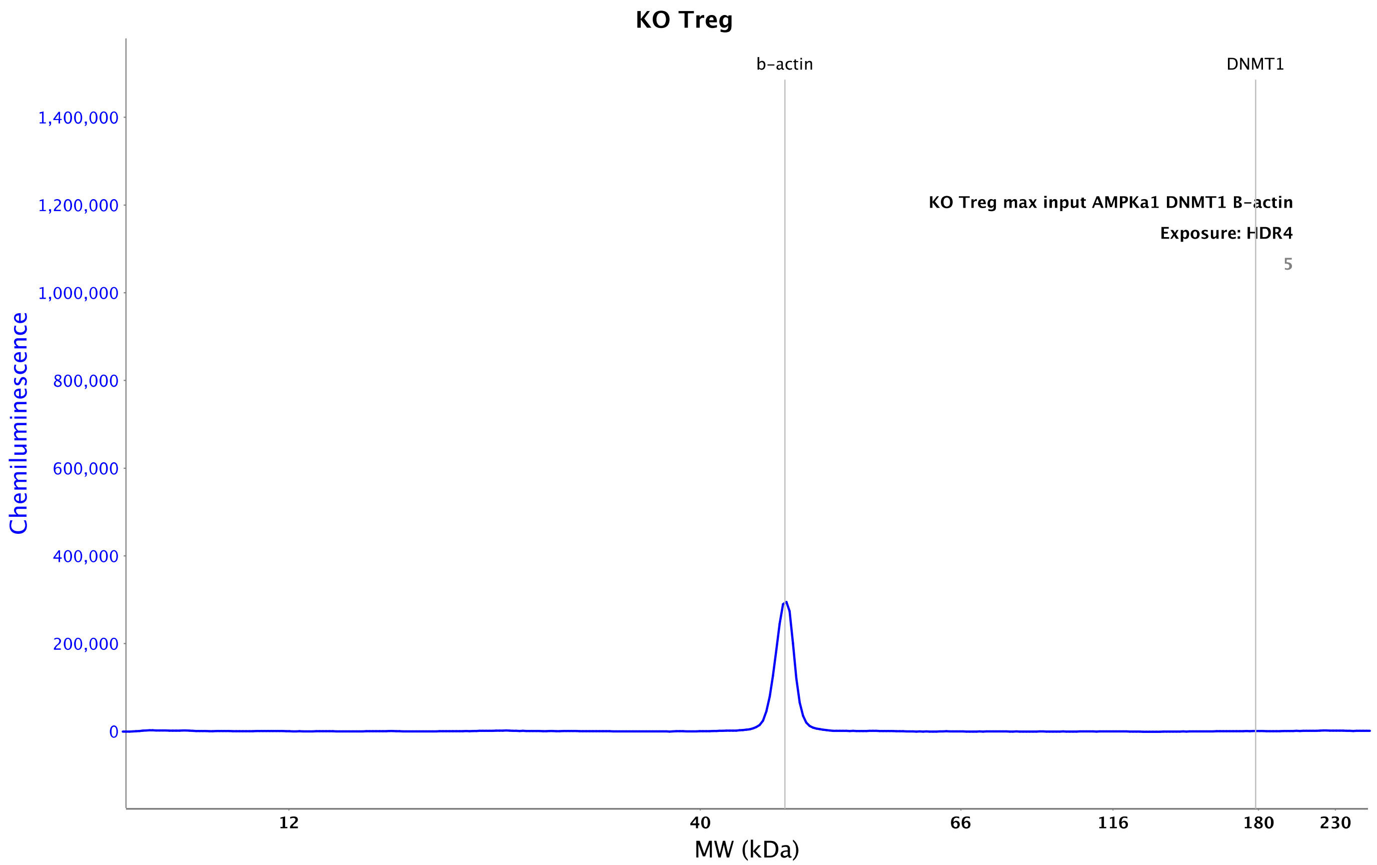

Figure 6F: DNMT1 IP iTreg

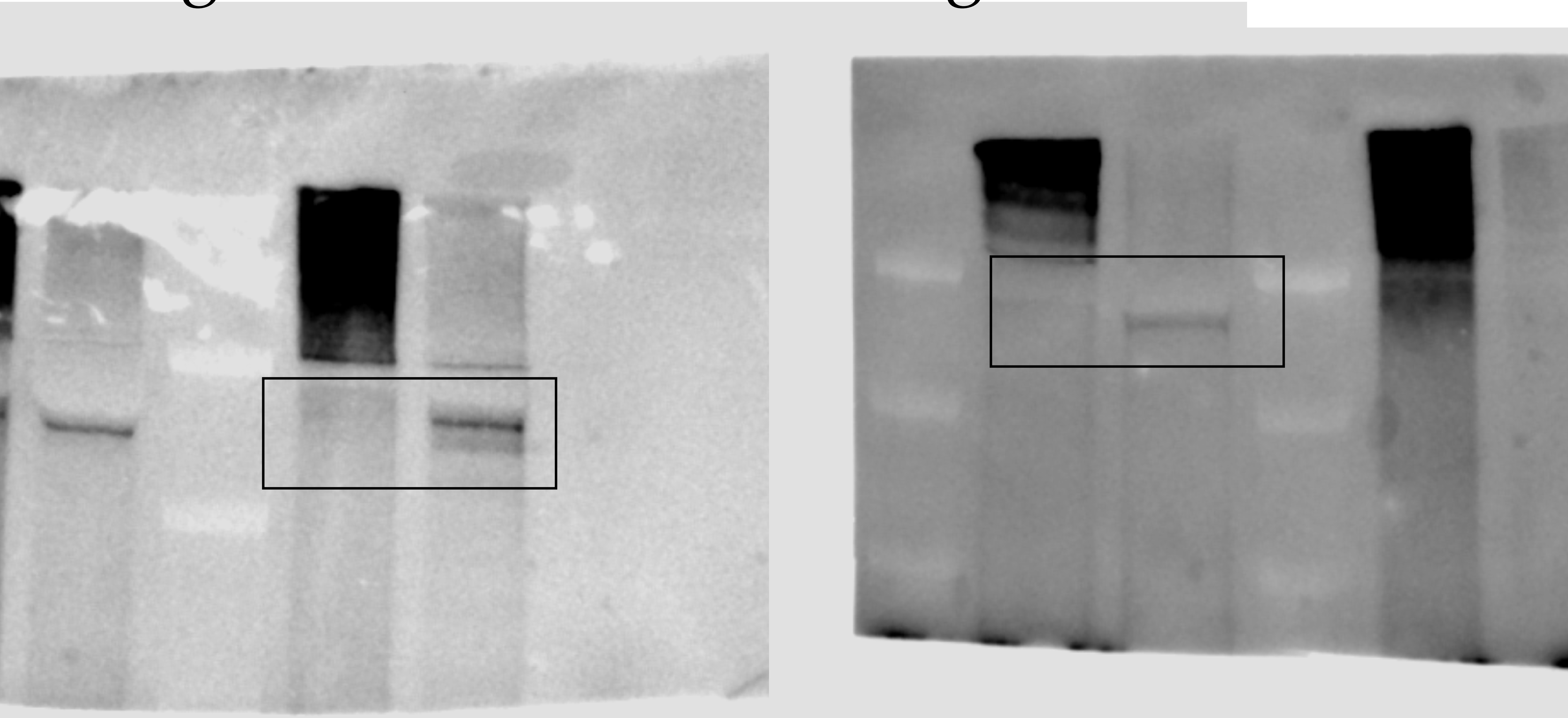

Figure 6F: AMPKa1 IP iTreg

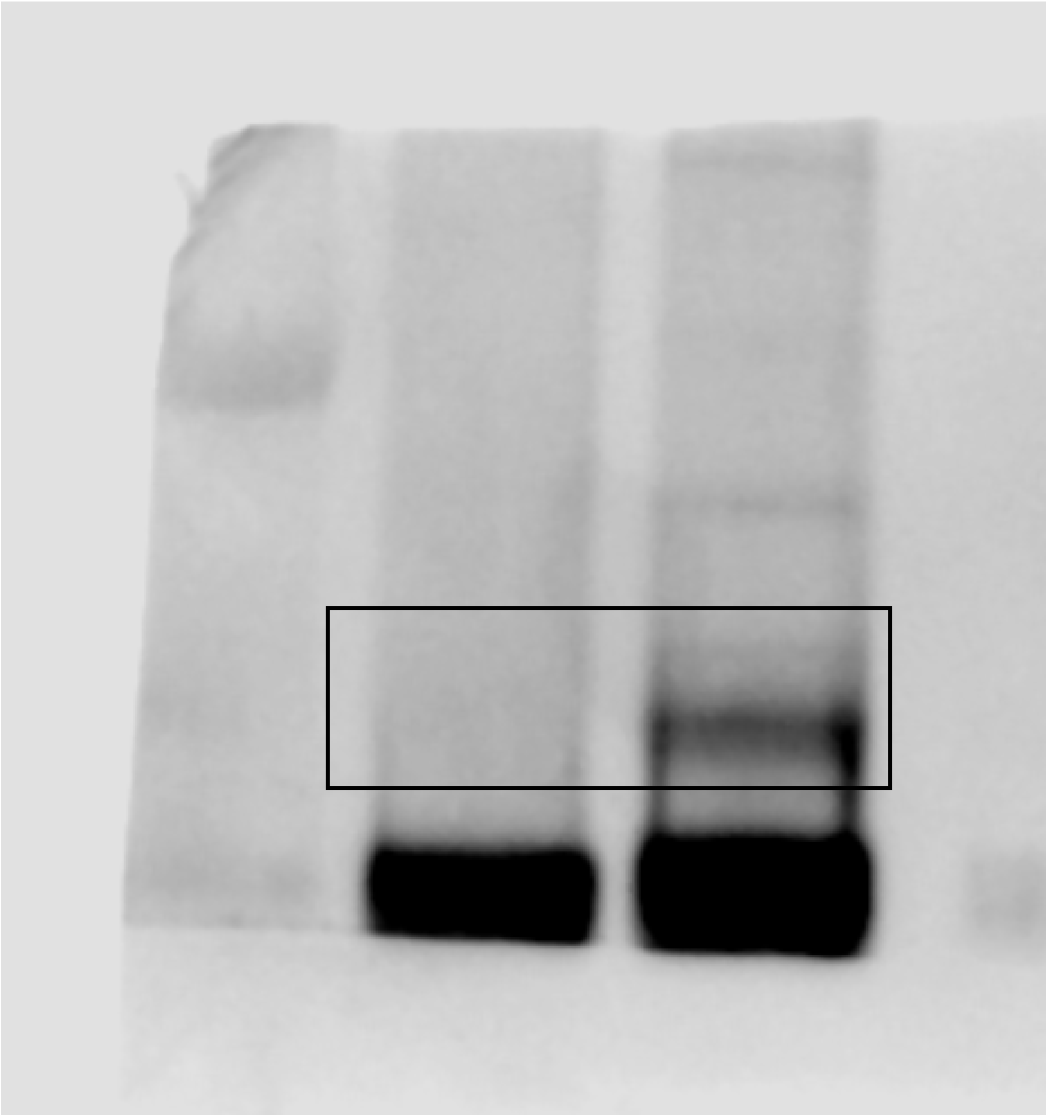

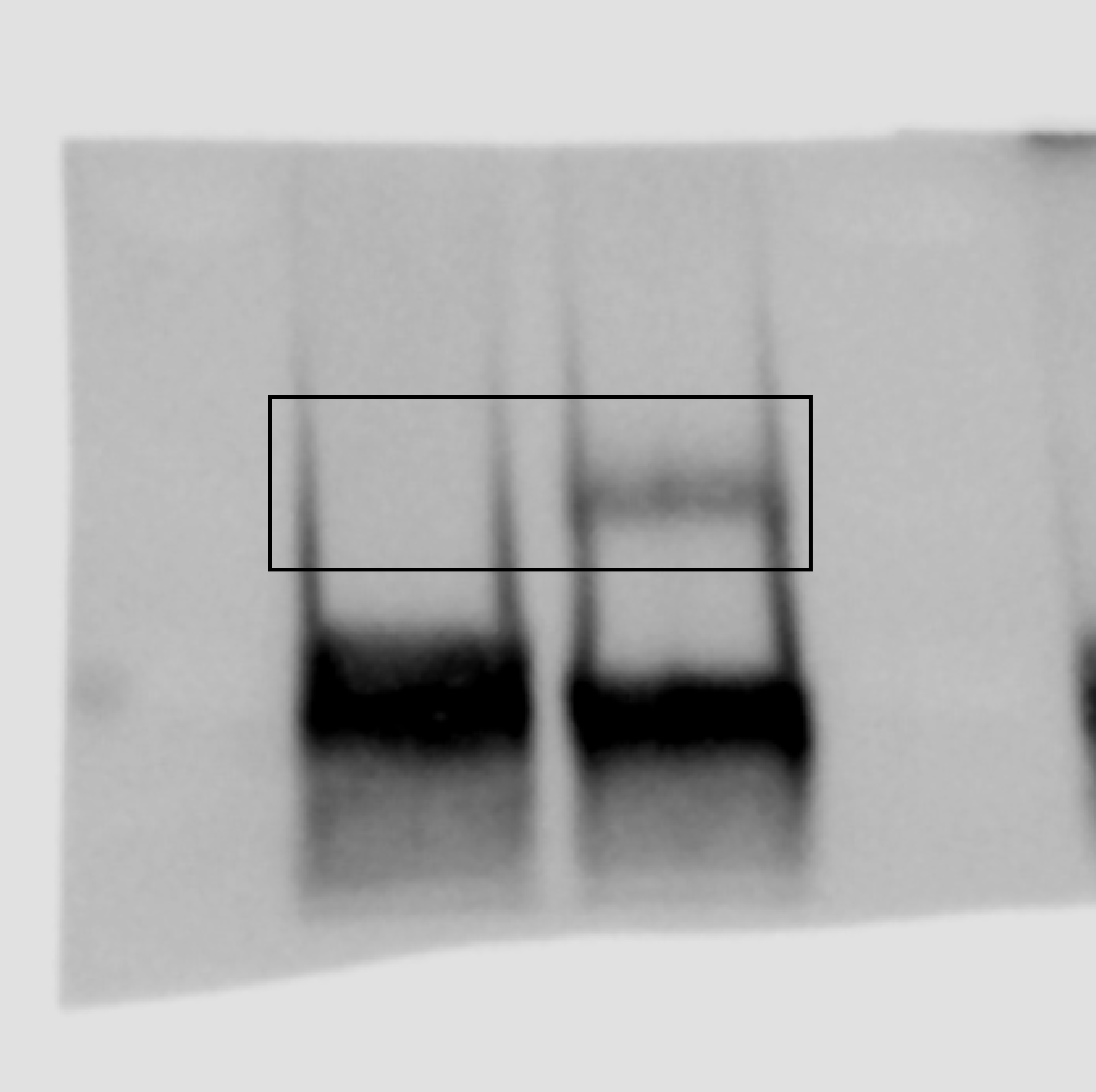

Supplemental Figure 1C: AMPK KO validation

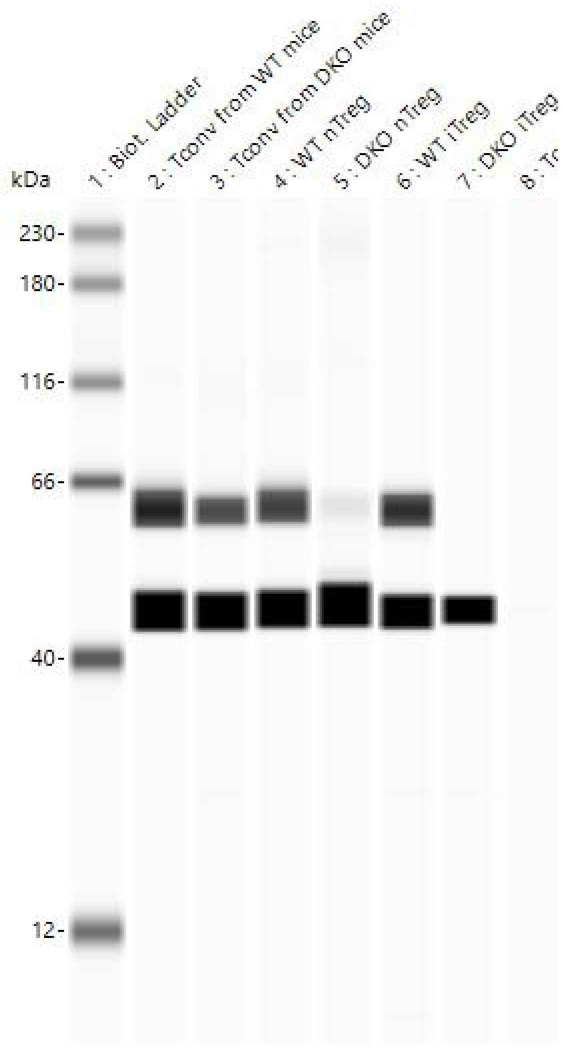

Supplemental Figure 1C: AMPK KO validation

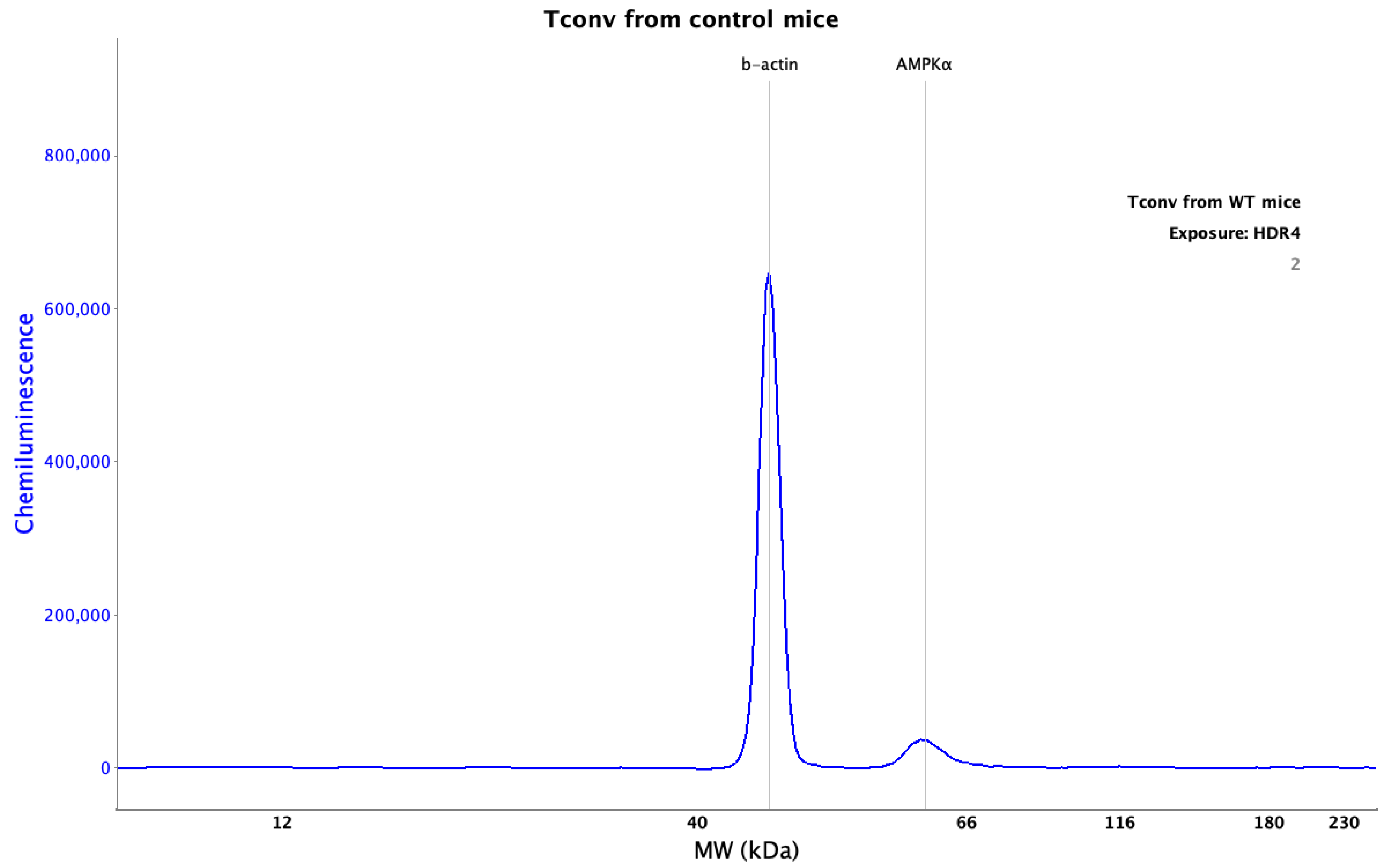

Supplemental Figure 1C: AMPK KO validation

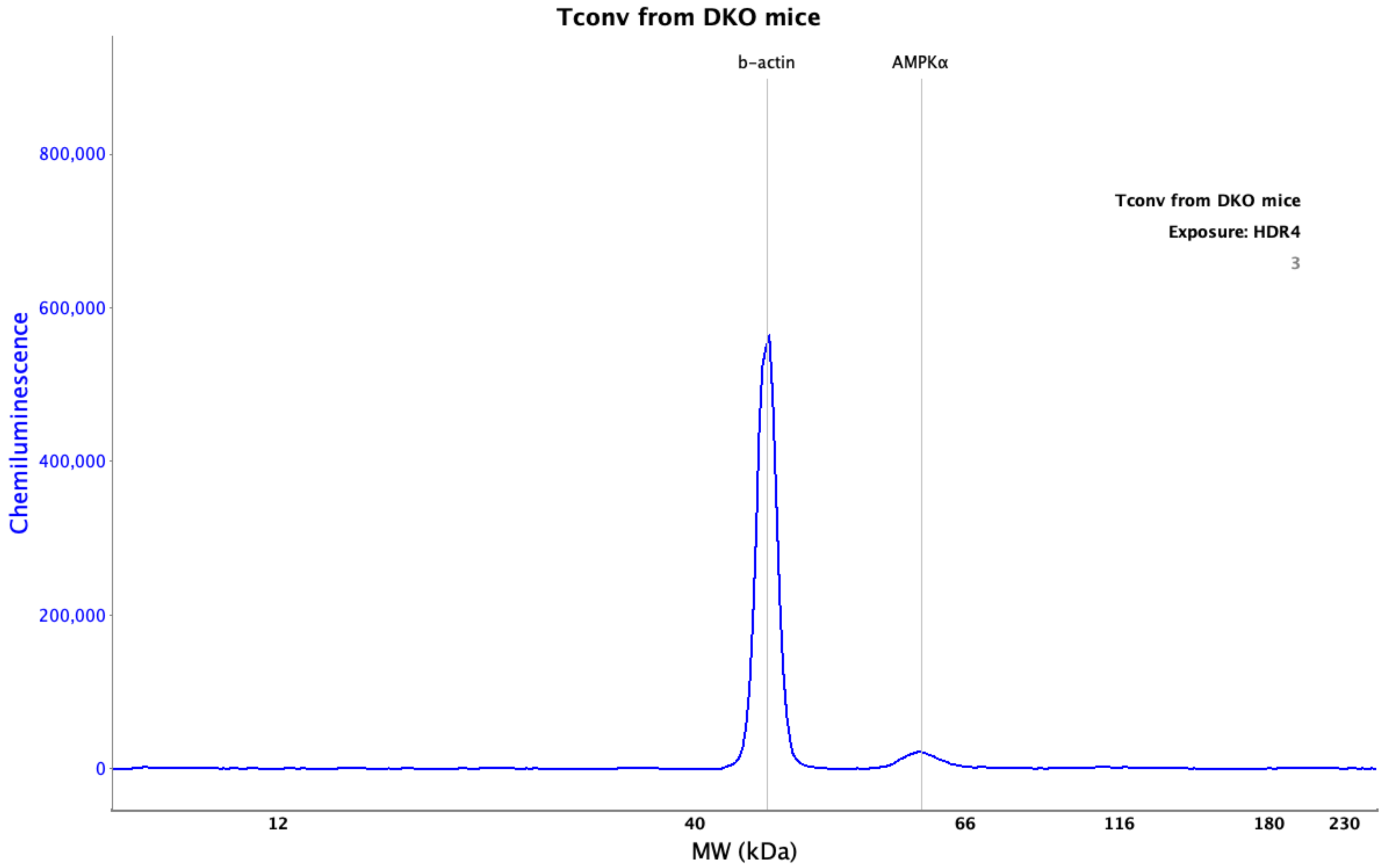

Supplemental Figure 1C: AMPK KO validation

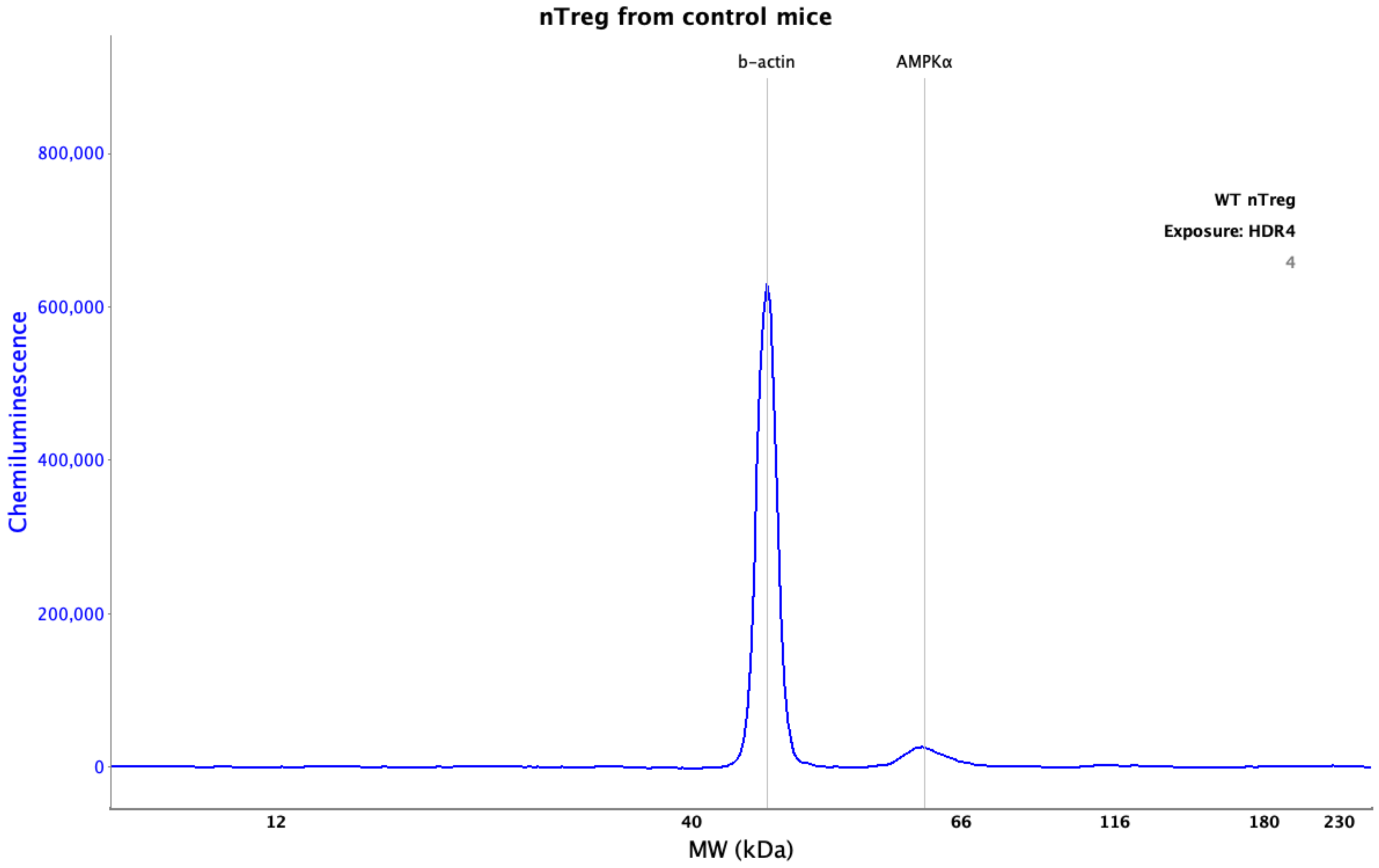

Supplemental Figure 1C: AMPK KO validation

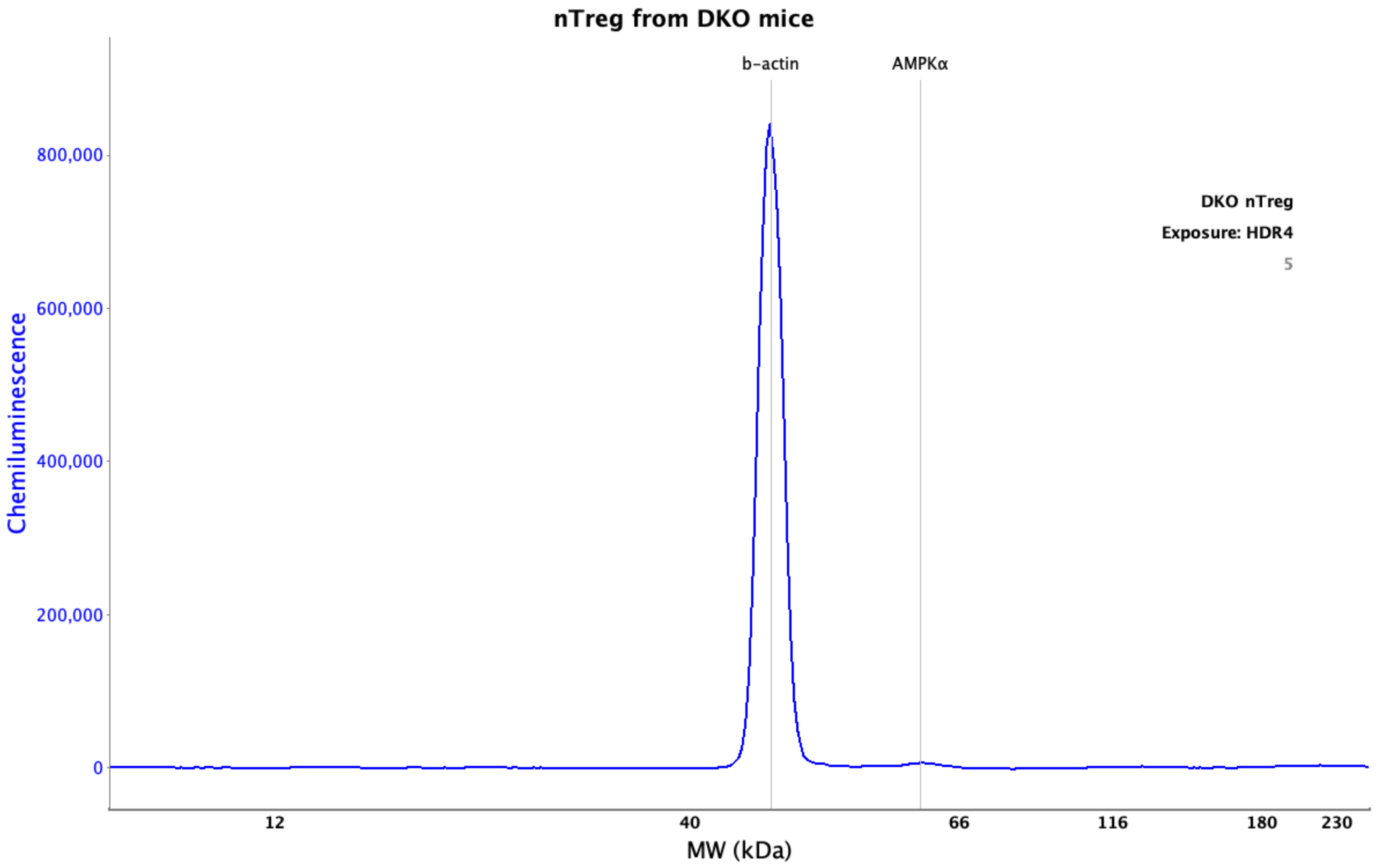

Supplemental Figure 1C: AMPK KO validation

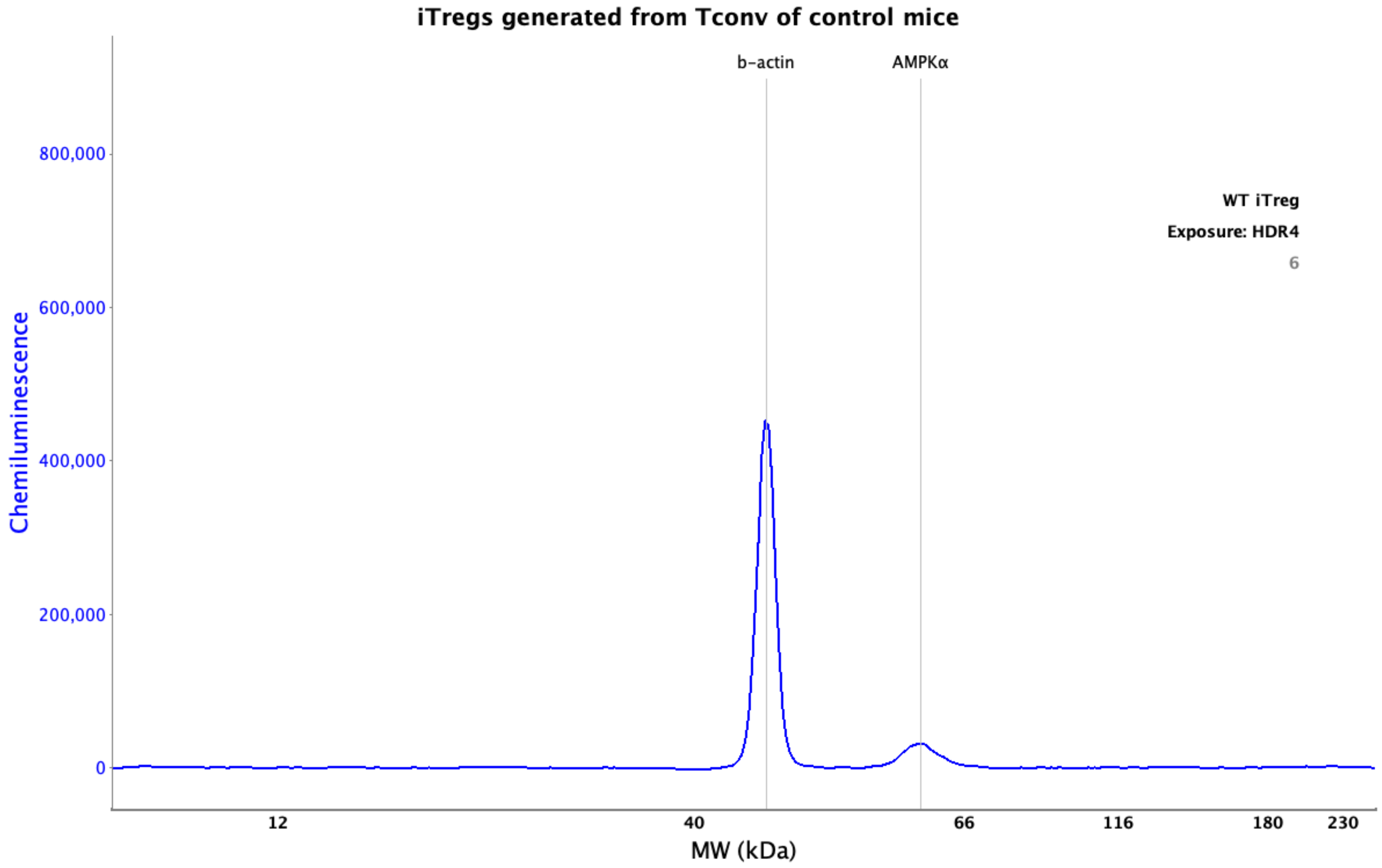

Supplemental Figure 1C: AMPK KO validation

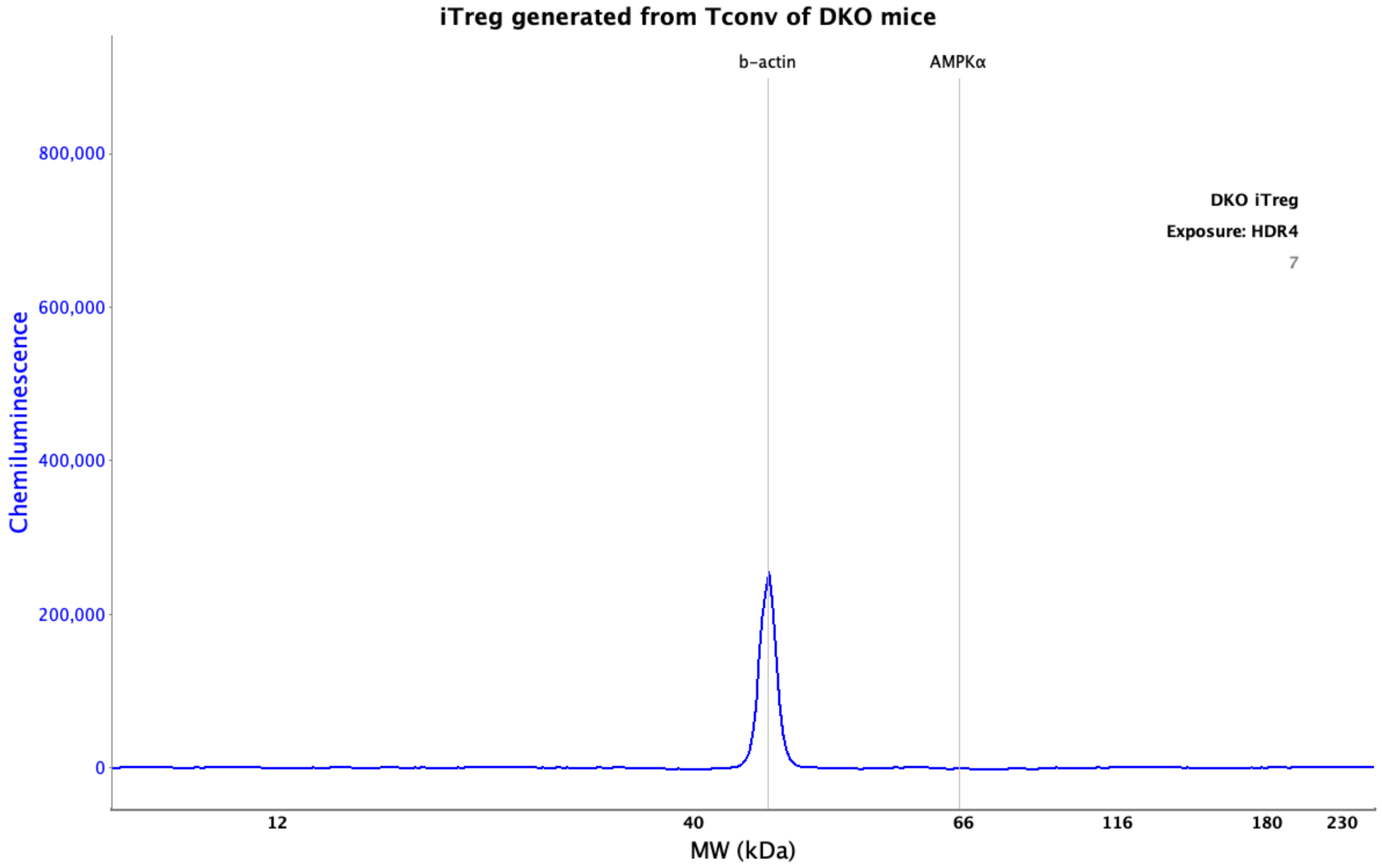

Supplemental Figure 10A: DNMT1 protein quantification n Treg WT vs AMPKα single knockouts

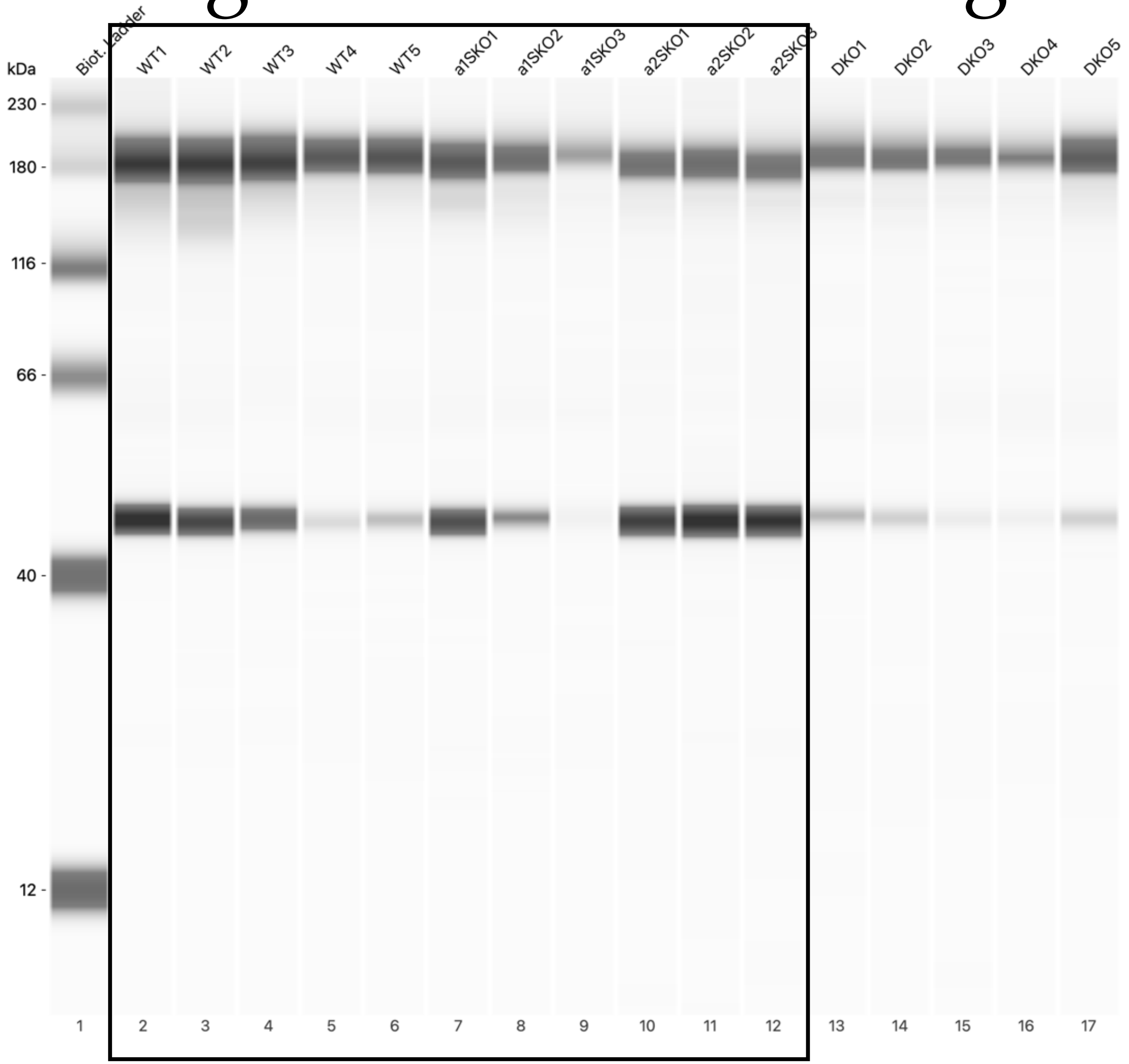

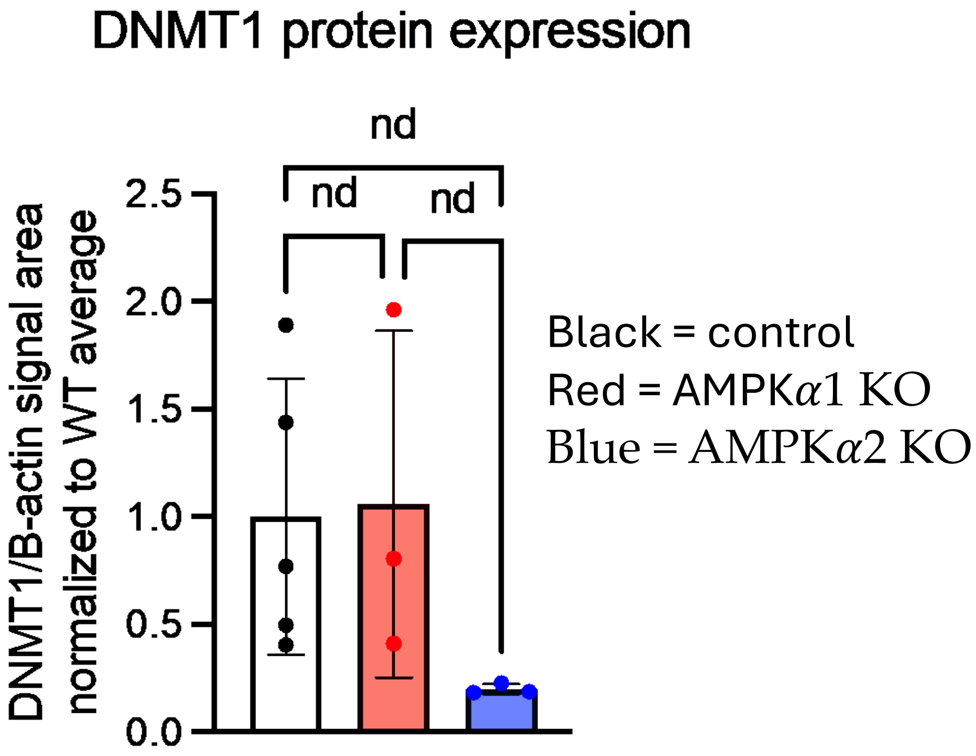

WT1-5 also shown in Figure 6D

DKO1-5 shown in Figure 6D

Supplemental Figure 10A: DNMT1 protein quantification n Treg WT vs AMPKα single knockouts

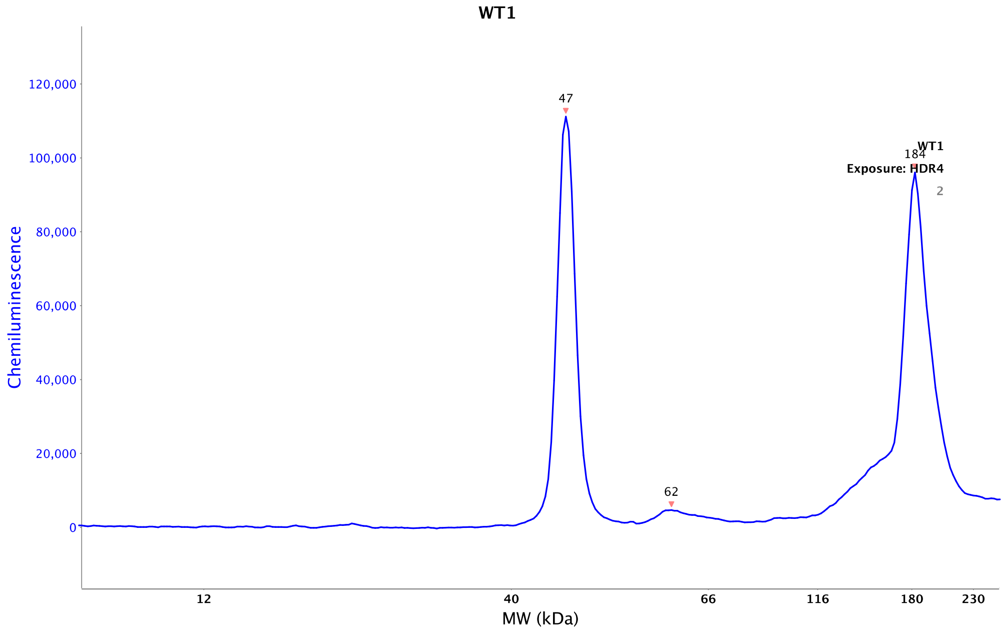

* Also shown in Figure 6D

Supplemental Figure 10A: DNMT1 protein quantification n Treg WT vs AMPKα single knockouts

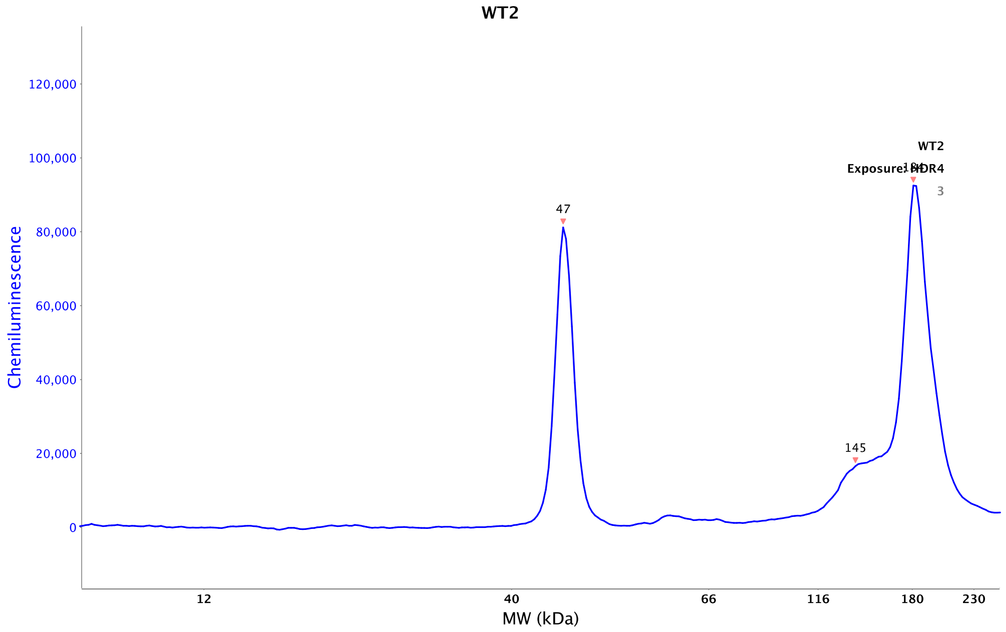

* Also shown in Figure 6D

Supplemental Figure 10A: DNMT1 protein quantification n Treg WT vs AMPKα single knockouts

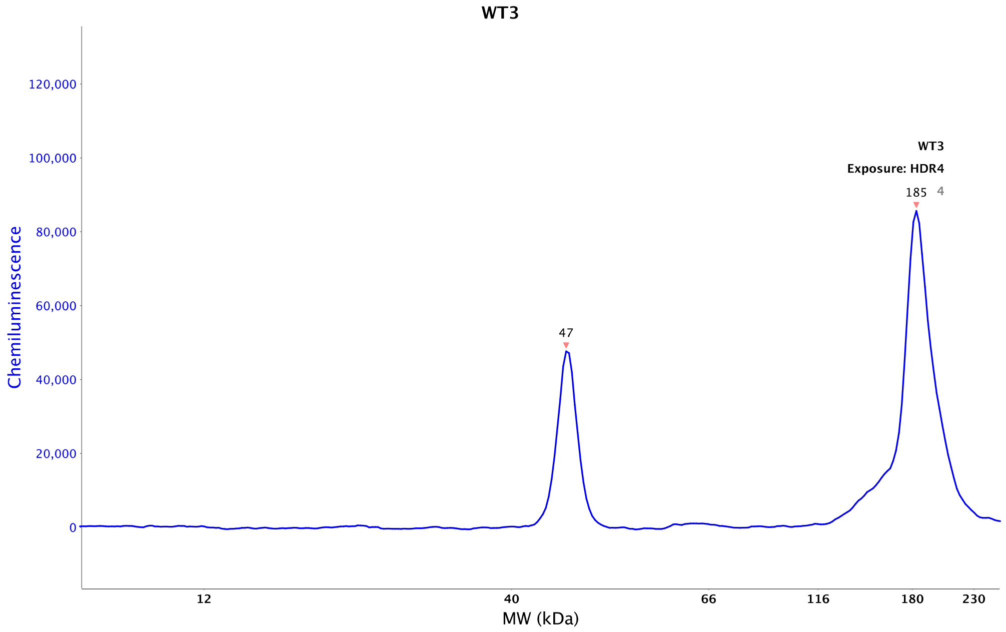

* Also shown in Figure 6D

Supplemental Figure 10A: DNMT1 protein quantification n Treg WT vs AMPKα single knockouts

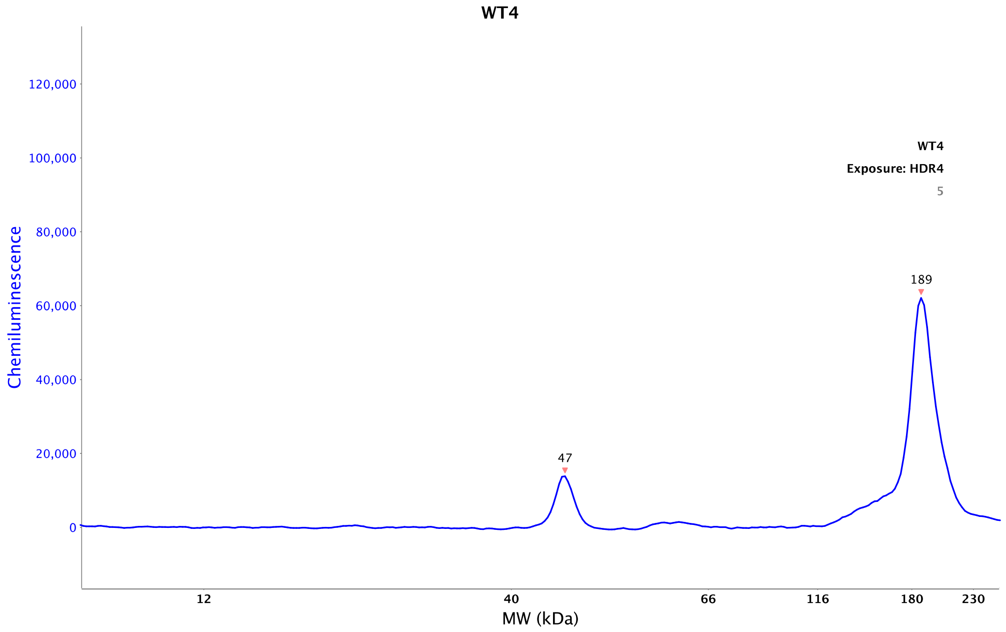

* Also shown in Figure 6D

Supplemental Figure 10A: DNMT1 protein quantification n Treg WT vs AMPKα single knockouts

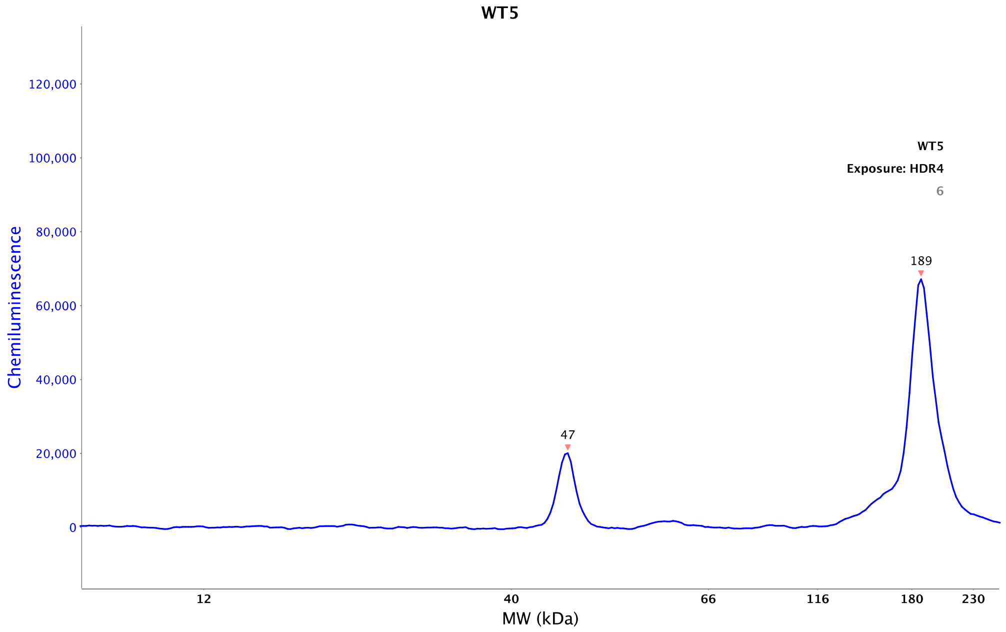

* Also shown in Figure 6D

Supplemental Figure 10A: DNMT1 protein quantification n Treg WT vs AMPKα single knockouts

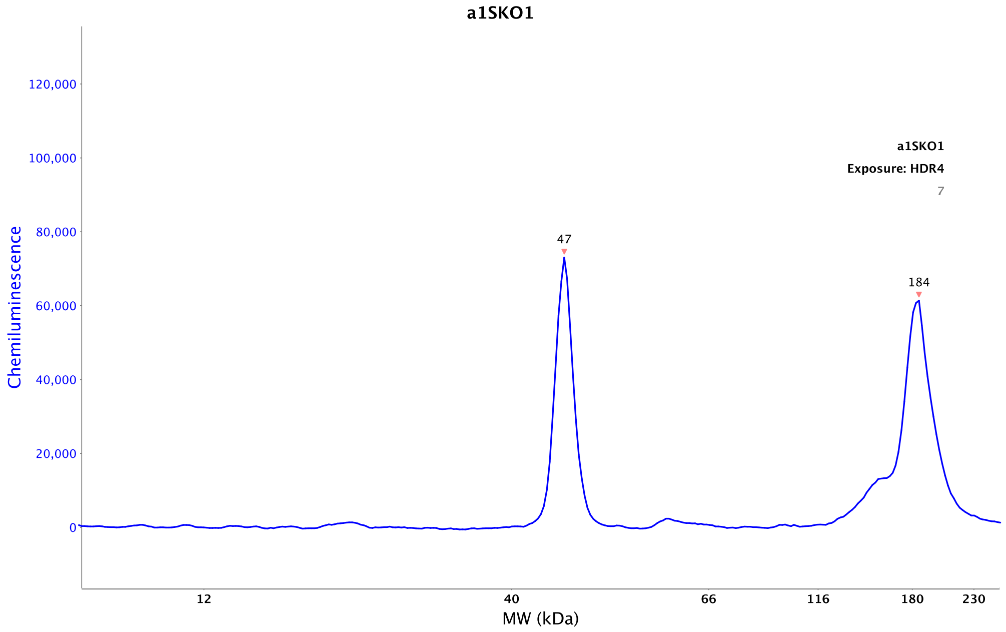

Supplemental Figure 10A: DNMT1 protein quantification n Treg WT vs AMPKα single knockouts

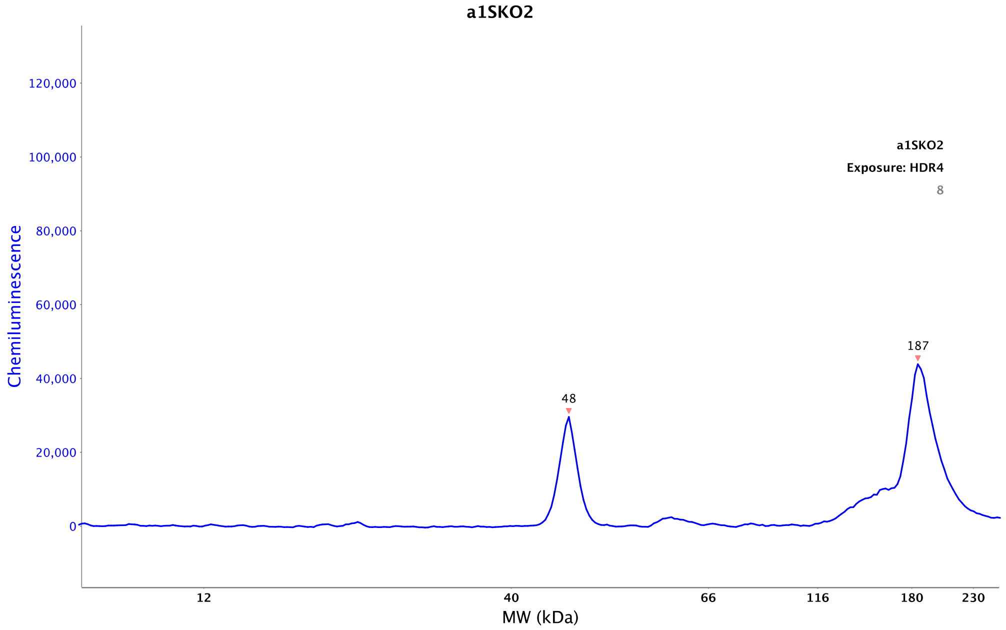

Supplemental Figure 10A: DNMT1 protein quantification n Treg WT vs AMPKα single knockouts

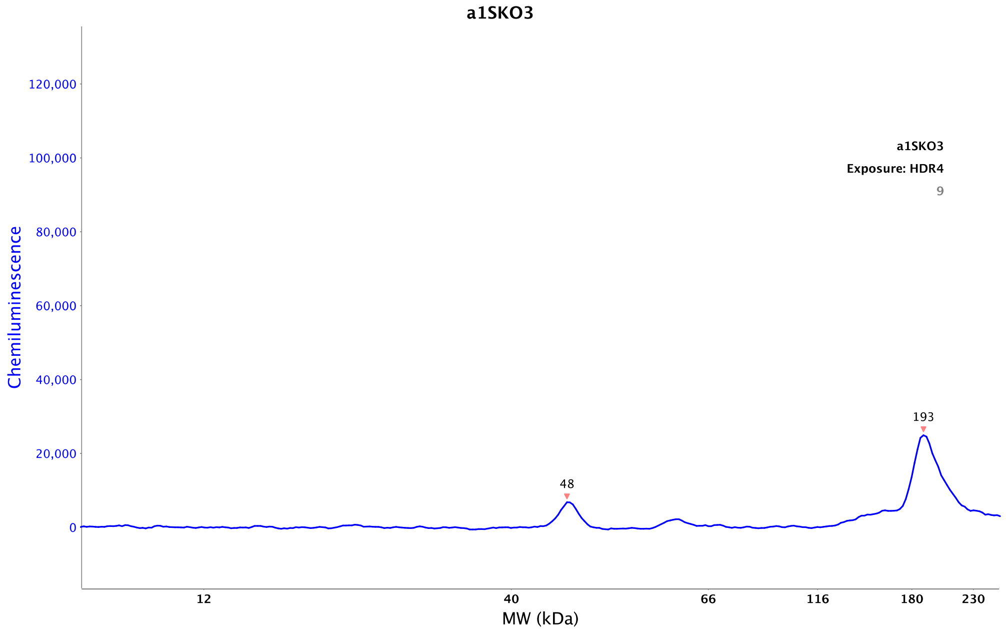

Supplemental Figure 10A: DNMT1 protein quantification n Treg WT vs AMPKα single knockouts

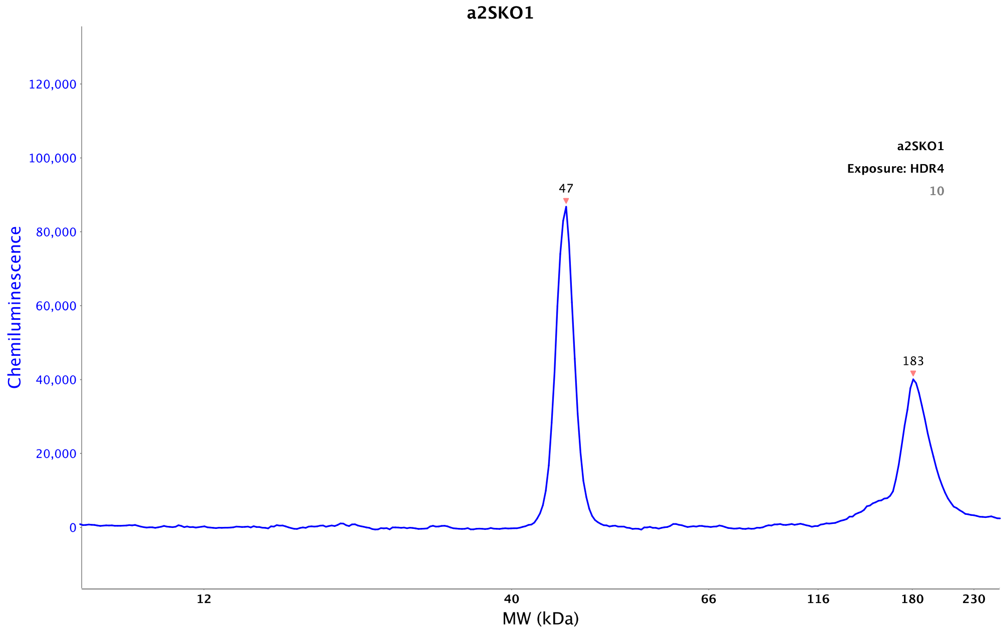

Supplemental Figure 10A: DNMT1 protein quantification n Treg WT vs AMPKα single knockouts

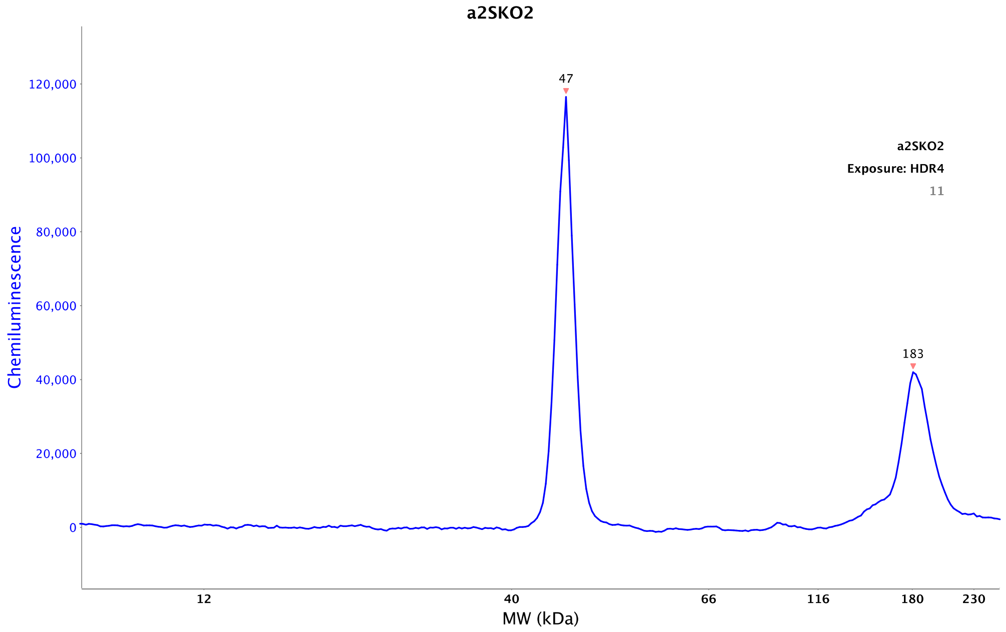

Supplemental Figure 10A: DNMT1 protein quantification n Treg WT vs AMPKα single knockouts

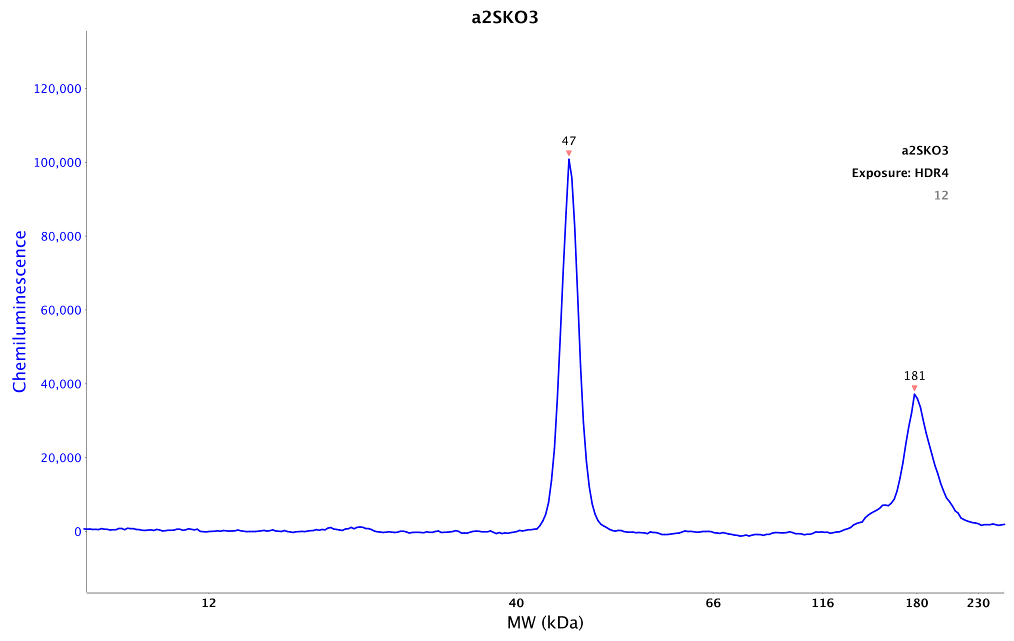

Supplemental Figure 10B: DNMT1 IP Jurkat

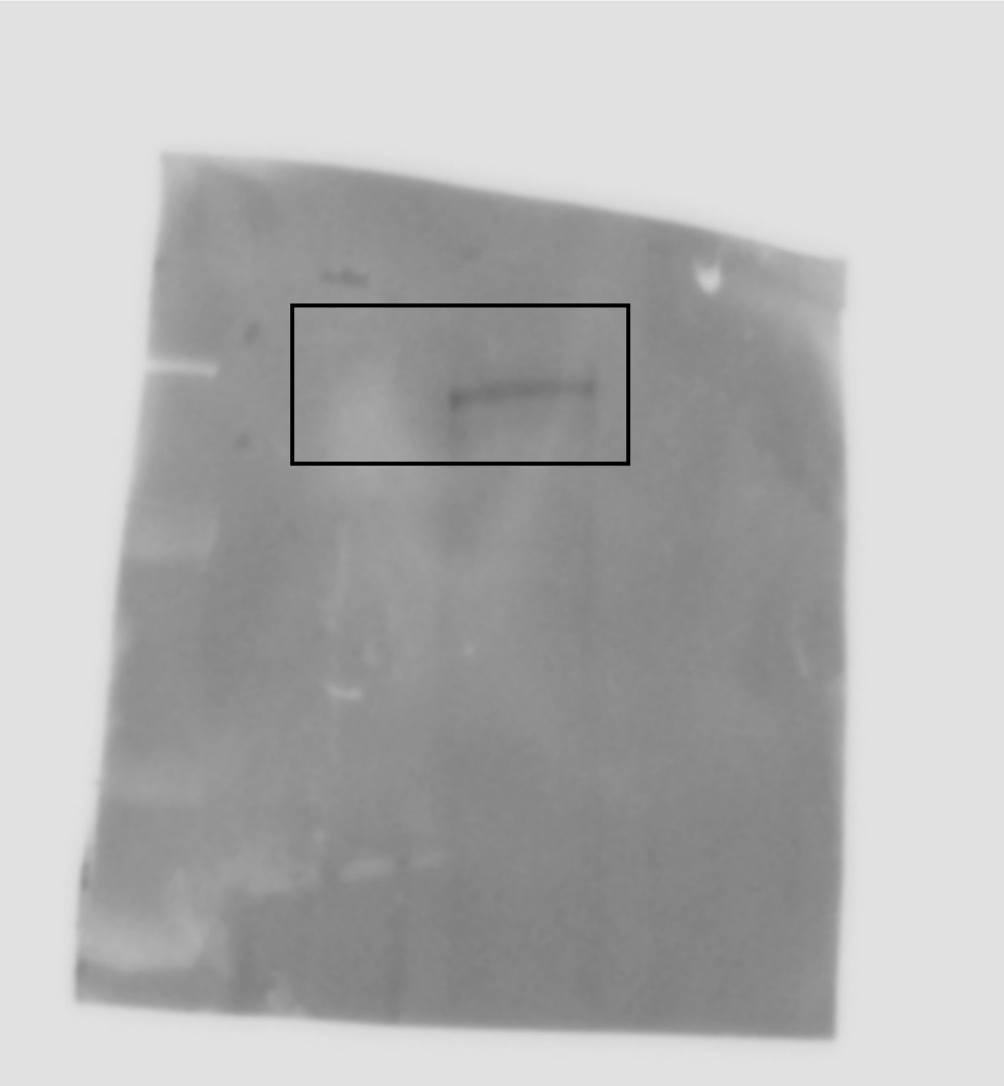

Supplemental Figure 10B: AMPKα1 IP Jurkat

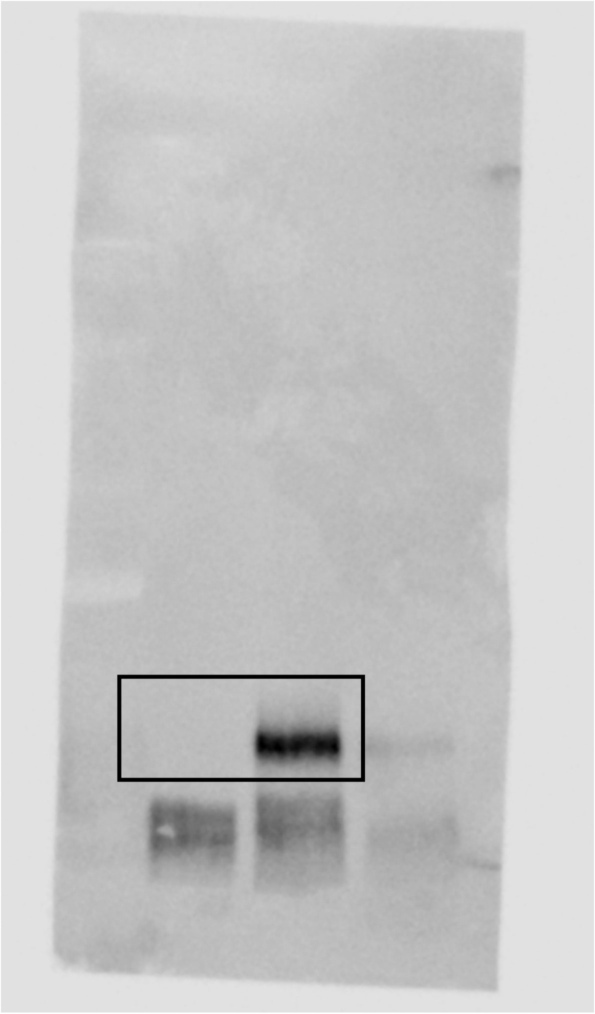

Supplemental Figure 10B: AMPKα1 input Jurkat

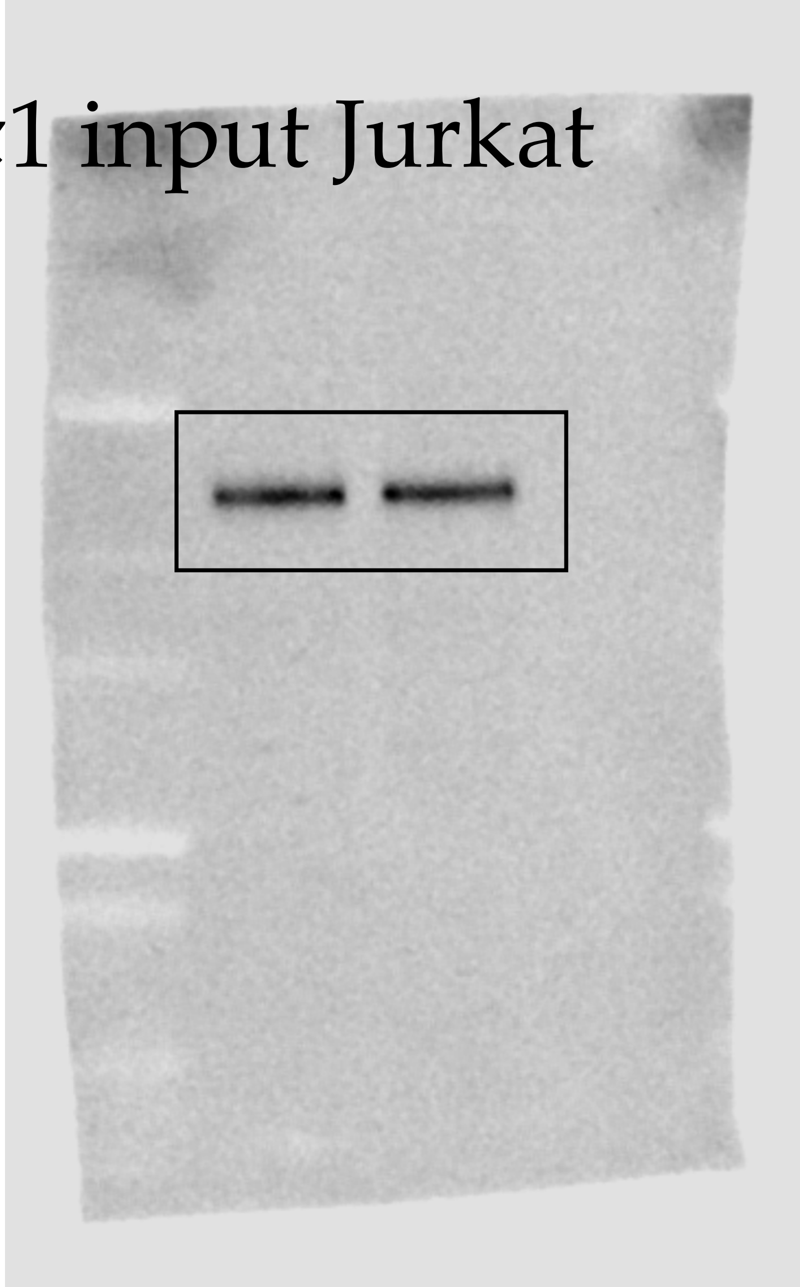

Supplemental Figure 10B: Actin input Jurkat

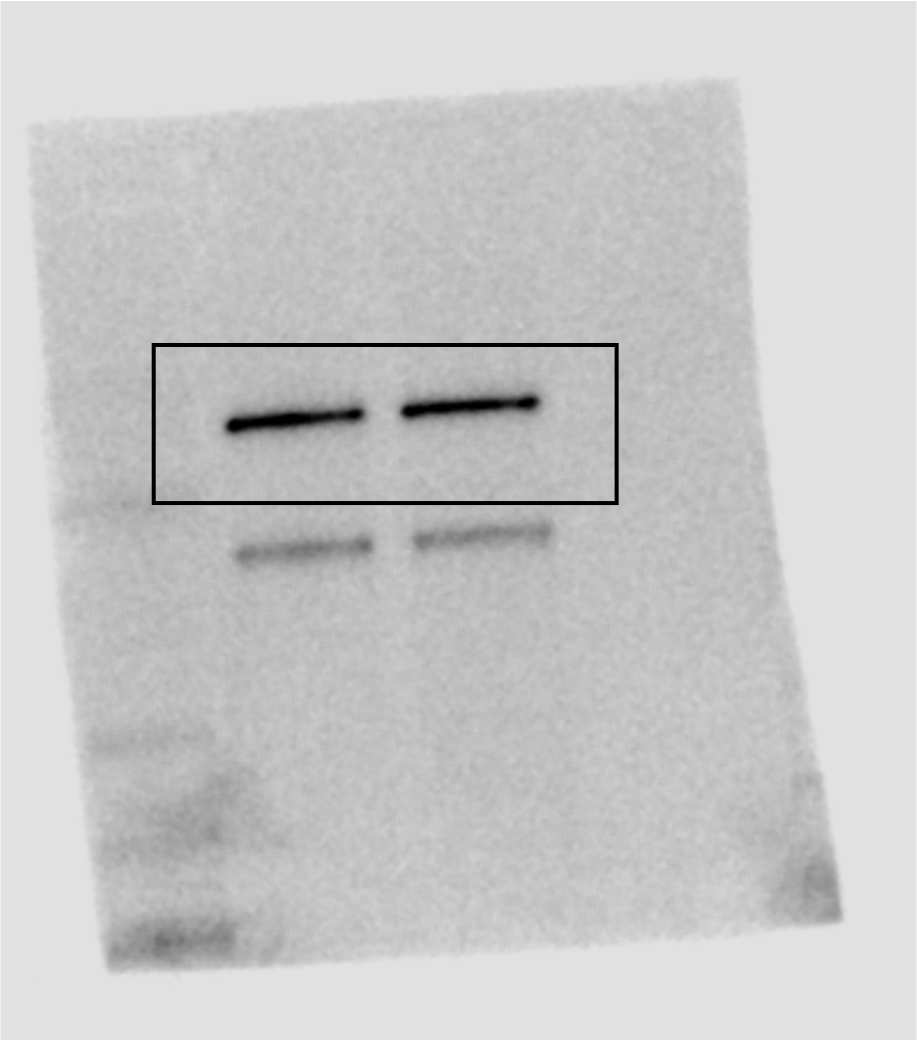

Supplemental Figure 10C: DNMT1 IP MT-2

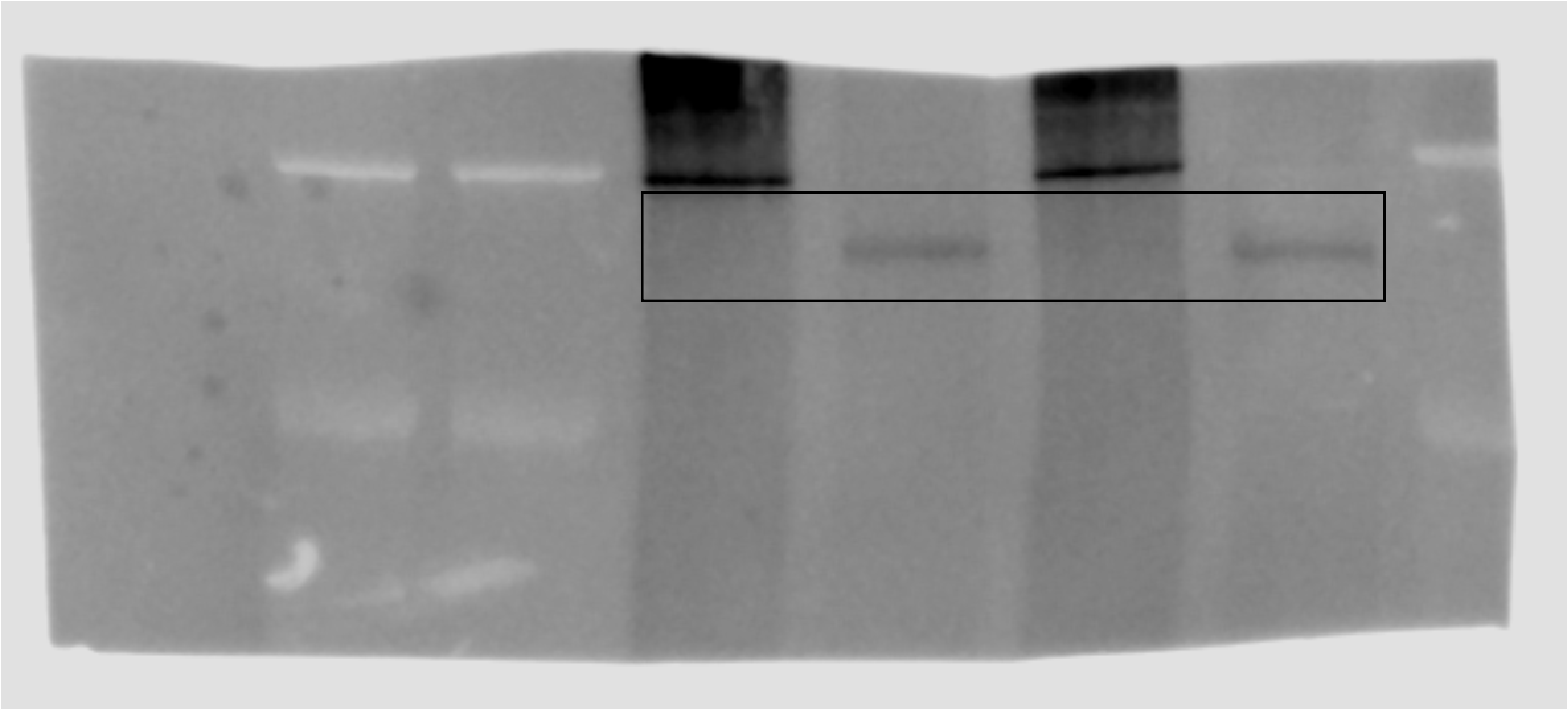

Supplemental Figure 10C: AMPKα1 IP MT-2

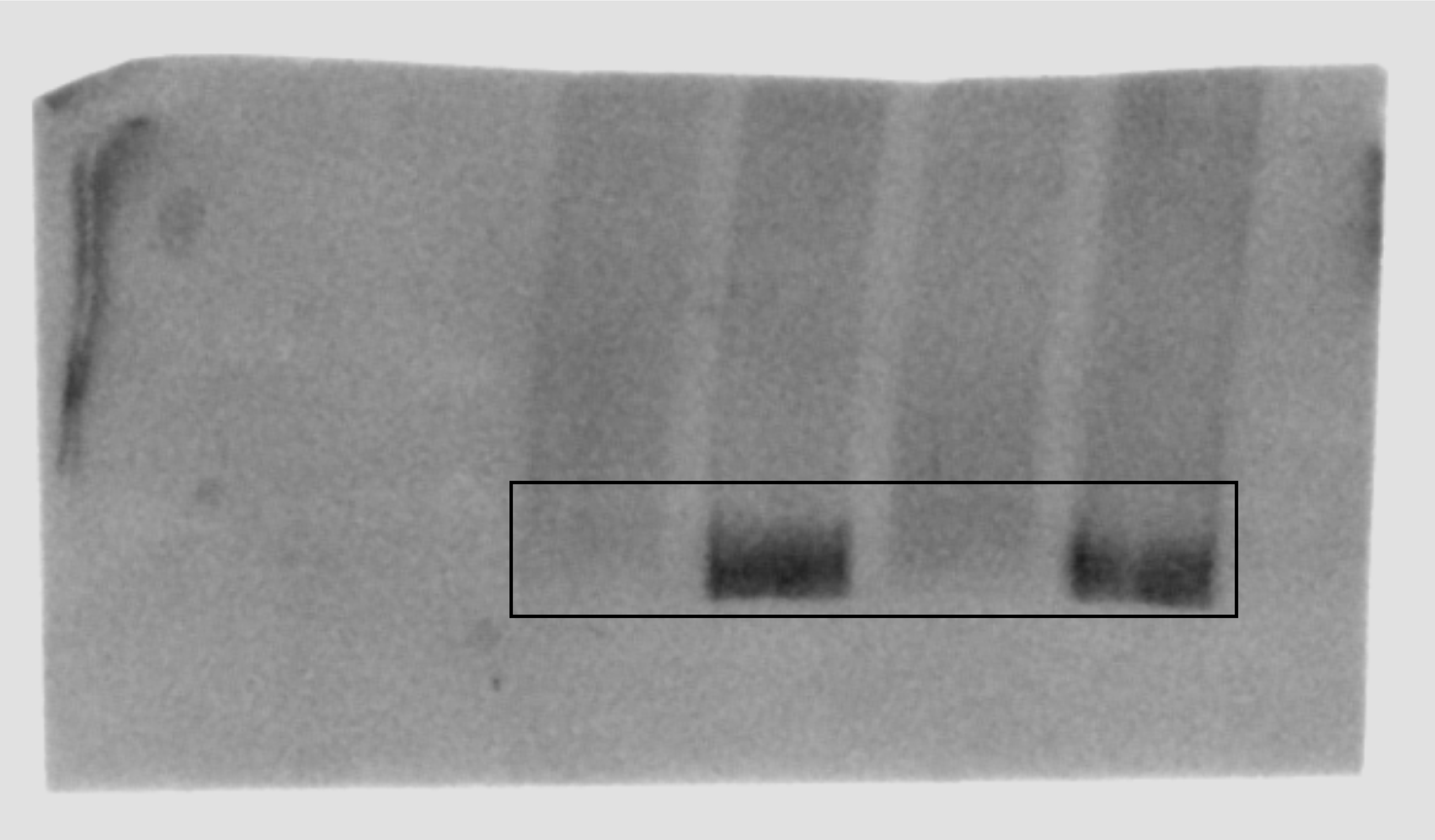

Supplemental Figure 10C: DNMT1 input MT-2

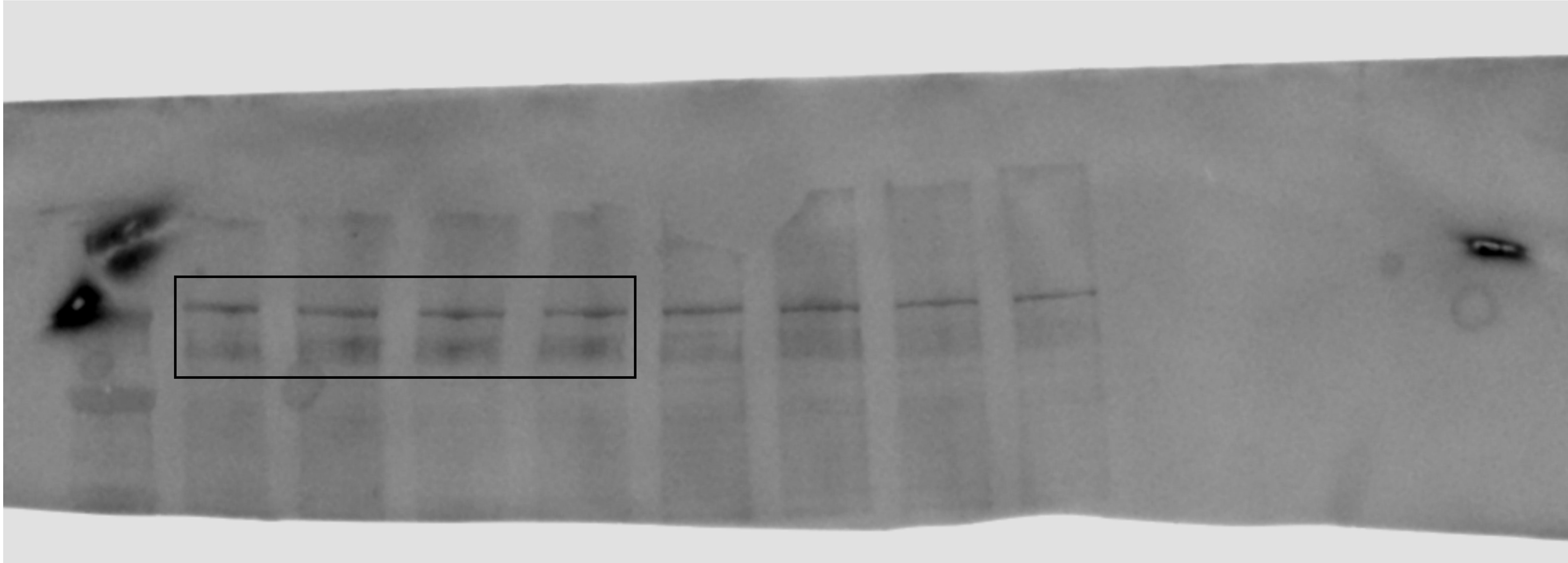

Supplemental Figure 10C: AMPKα1 input MT-2

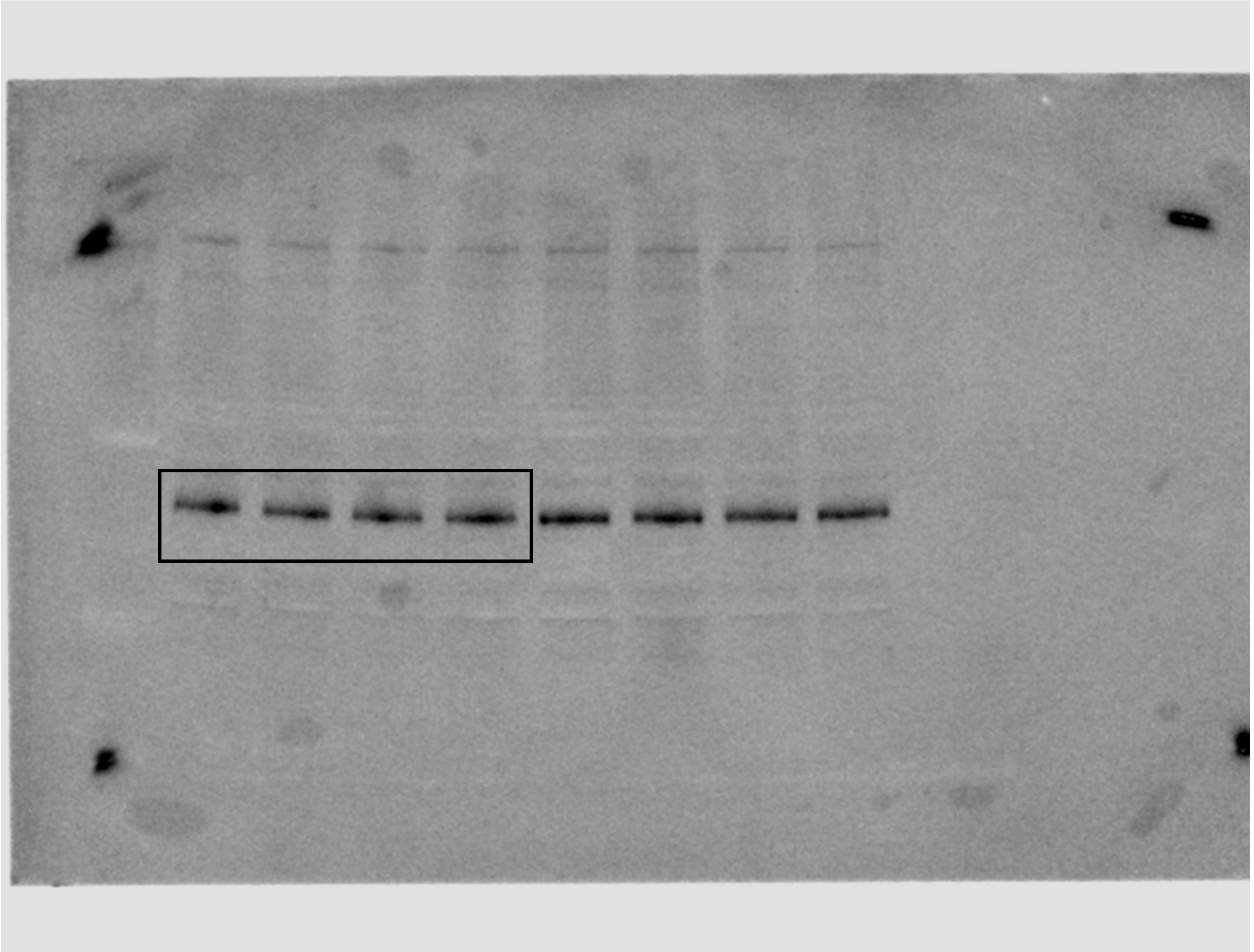

Supplemental Figure 10C: Actin input MT-2

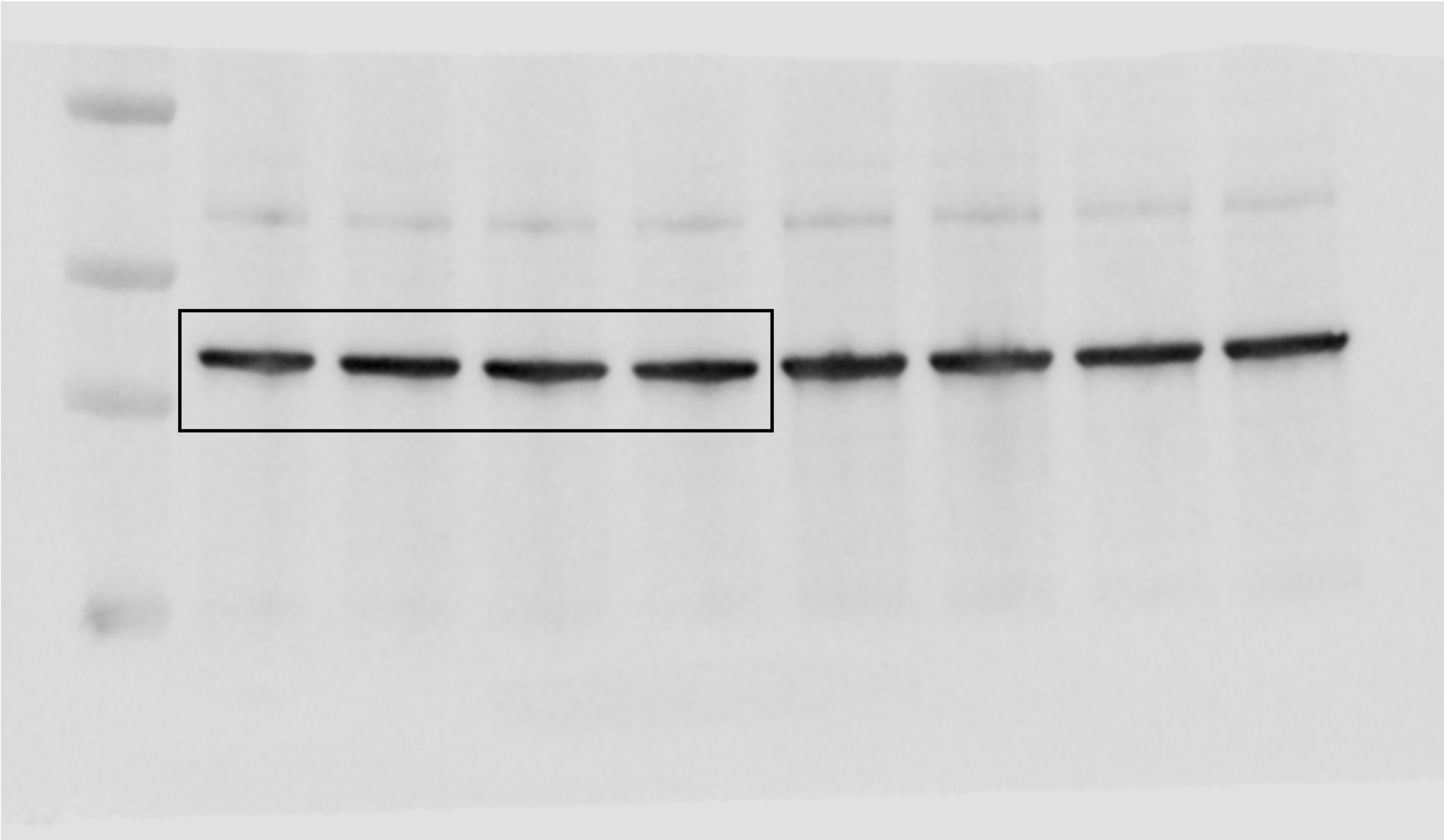

Supplemental Figure 10F: DNMT1 MT-2 fractionation

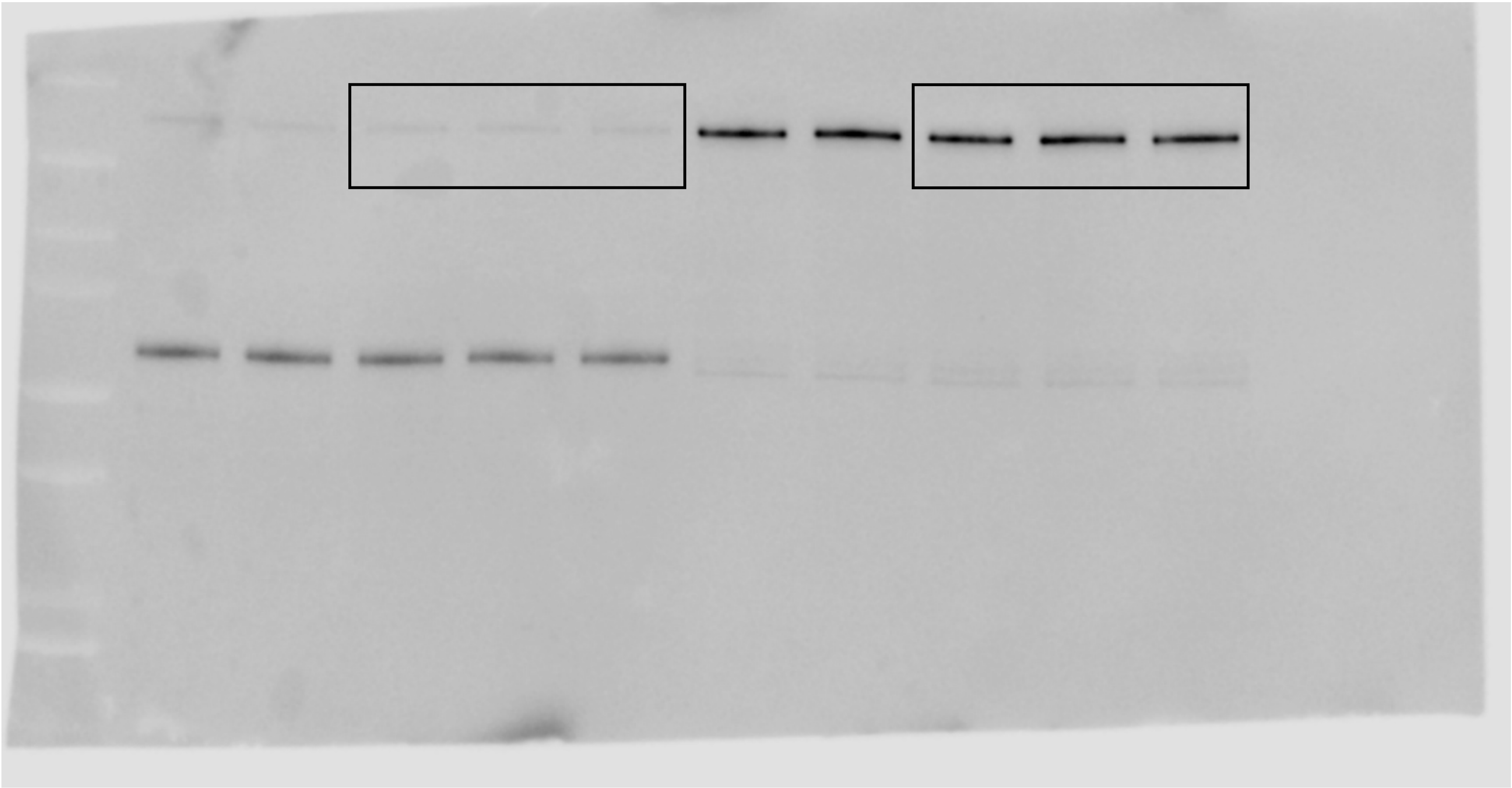

Supplemental Figure 10F: AMPKα1 MT-2 fractionation

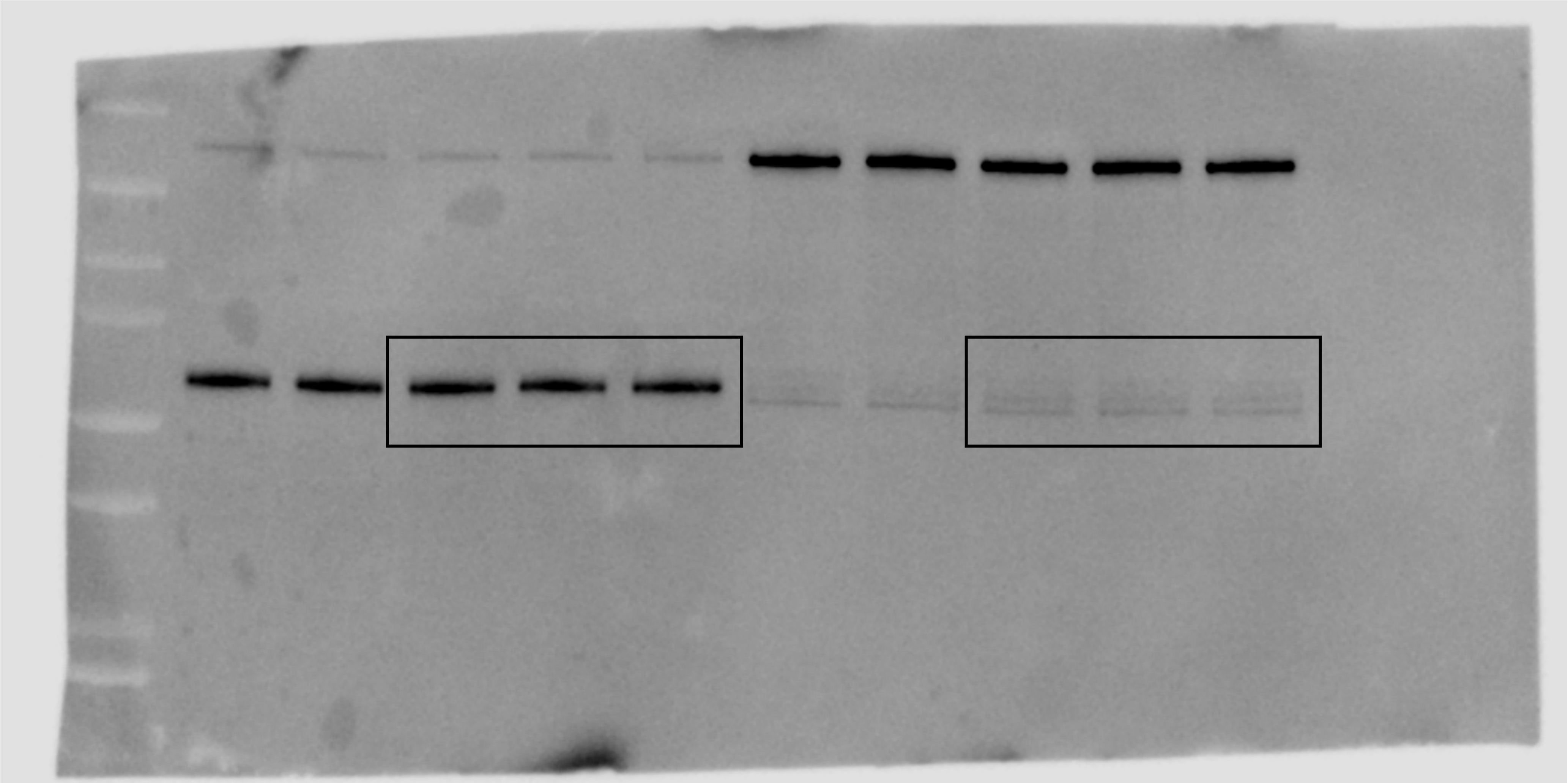

Supplemental Figure 10F: Actin MT-2 fractionation

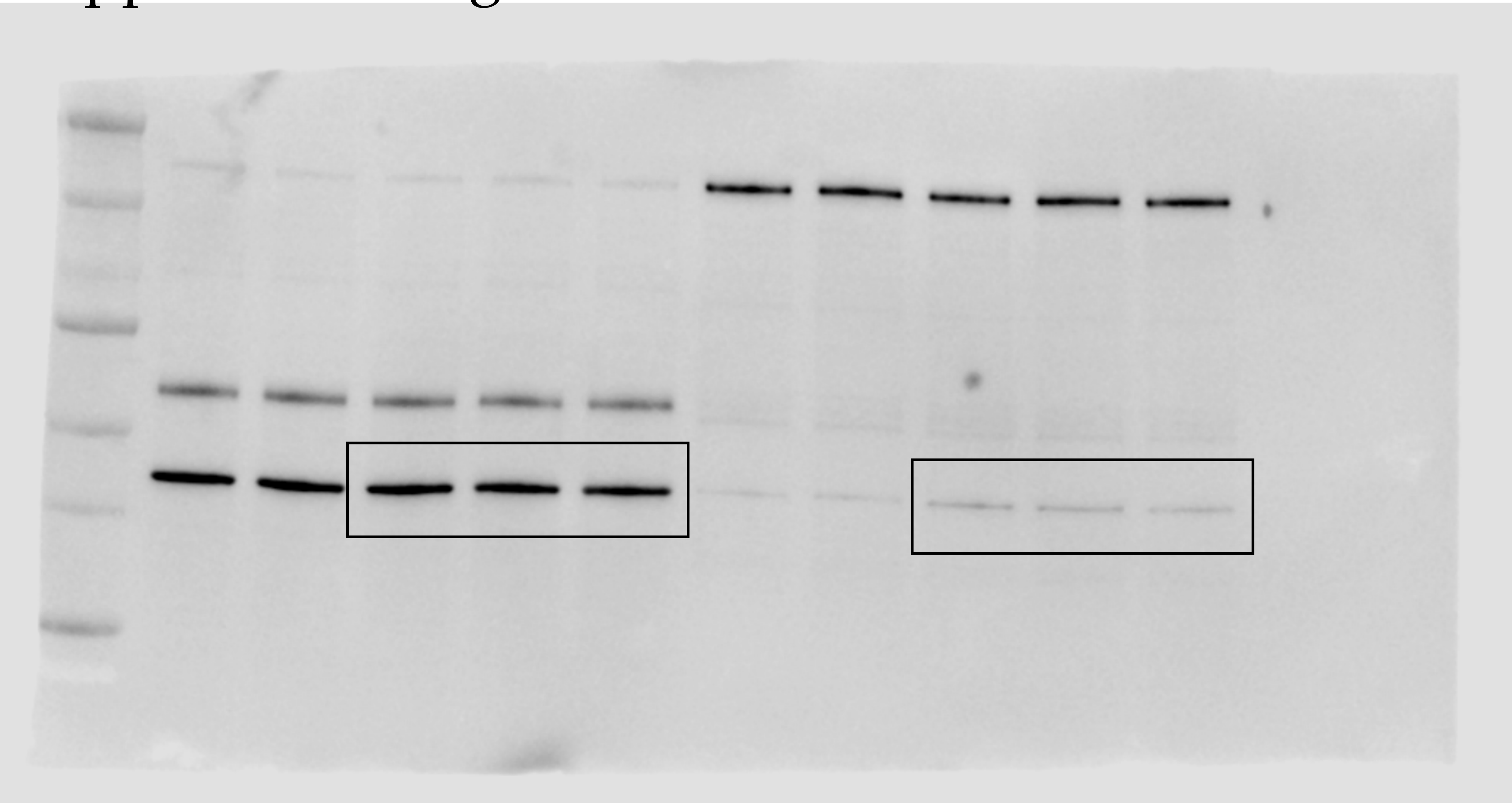

Supplement: Unedited blot and gel images [file jci-135-179572-s016.pdf]
